# *PiggyBac* mutagenesis and exome sequencing identify genetic driver landscapes and potential therapeutic targets of *EGFR*-mutant gliomas

**DOI:** 10.1186/s13059-020-02092-2

**Published:** 2020-07-30

**Authors:** Imran Noorani, Jorge de la Rosa, Yoonha Choi, Alexander Strong, Hannes Ponstingl, M. S. Vijayabaskar, Jusung Lee, Eunmin Lee, Angela Richard-Londt, Mathias Friedrich, Federica Furlanetto, Rocio Fuente, Ruby Banerjee, Fengtang Yang, Frances Law, Colin Watts, Roland Rad, George Vassiliou, Jong Kyoung Kim, Thomas Santarius, Sebastian Brandner, Allan Bradley

**Affiliations:** 1 The Wellcome Trust Sanger Institute, Wellcome Trust Genome Campus, Hinxton, Cambridgeshire, CB10 1SA UK; 2 Department of Neurosurgery, Addenbrookes Hospital, University of Cambridge, Hills Road, Cambridge, CB2 0QQ UK; 3 Department of New Biology, DGIST, 333, Techno Jungang Daero, Hyeonpung-Myeon, Dalseong-Gun, Daegu, 42988 South Korea; 4 Division of Neuropathology and Department of Neurodegenerative Disease, UCL Institute of Neurology, Queen Square, Mailbox 126, London, WC1N 3BG UK; 5 Department of Internal Medicine II, Klinikum rechts der Isar, Technische Universität München, Ismaninger Strasse 22, 81675 Munich, Germany; 6 Birmingham Brain Cancer Program, Institute of Cancer and Genomic Sciences, College of Medical and Dental Sciences, University of Birmingham, Edgbaston, Birmingham, B15 2TT UK

## Abstract

**Background:**

Glioma is the most common intrinsic brain tumor and also occurs in the spinal cord. Activating *EGFR* mutations are common in *IDH1* wild-type gliomas. However, the cooperative partners of *EGFR* driving gliomagenesis remain poorly understood.

**Results:**

We explore *EGFR*-mutant glioma evolution in conditional mutant mice by whole-exome sequencing, transposon mutagenesis forward genetic screening, and transcriptomics. We show mutant *EGFR* is sufficient to initiate gliomagenesis in vivo, both in the brain and spinal cord. We identify significantly recurrent somatic alterations in these gliomas including mutant *EGFR* amplifications and *Sub1*, *Trp53*, and *Tead2* loss-of-function mutations. Comprehensive functional characterization of 96 gliomas by genome-wide *piggyBac* insertional mutagenesis in vivo identifies 281 known and novel *EGFR*-cooperating driver genes, including *Cdkn2a*, *Nf1*, *Spred1*, and *Nav3*. Transcriptomics confirms transposon-mediated effects on expression of these genes. We validate the clinical relevance of new putative tumor suppressors by showing these are frequently altered in patients’ gliomas, with prognostic implications. We discover shared and distinct driver mutations in brain and spinal gliomas and confirm in vivo differential tumor suppressive effects of *Pten* between these tumors. Functional validation with CRISPR-Cas9-induced mutations in novel genes *Tead2*, *Spred1*, and *Nav3* demonstrates heightened *EGFRvIII*-glioma cell proliferation. Chemogenomic analysis of mutated glioma genes reveals potential drug targets, with several investigational drugs showing efficacy in vitro.

**Conclusion:**

Our work elucidates functional driver landscapes of *EGFR*-mutant gliomas, uncovering potential therapeutic strategies, and provides new tools for functional interrogation of gliomagenesis.

## Introduction

Gliomas constitute 30% of all brain tumors. Their high-grade form, glioblastoma (GBM), accounts for 80% of malignant brain tumors and is one of the most lethal cancers, with median survival of 12.2 to 18.2 months [1–3]. Spinal gliomas (astrocytomas) form 60% of all spinal tumors in children and adolescents, with higher-grade lesions having a mean survival of 15.5 months. Surgical resection of spinal gliomas is challenging due to their infiltration of the spinal cord, presenting a barrier to molecular studies, and there is a lack of animal models.

Whole-genome sequence analysis of human brain gliomas has shed some light on the genetic and epigenetic landscapes of this disease [4–8]. Aside from mutations, driver genes may also be altered through transcriptional, methylation, or large-scale copy number changes making the affected genes more difficult to pinpoint as drivers. A further complicating issue is that mutations in individual gliomas can affect different genes in various combinations. This can alter prognosis and response to therapy [9, 10] and poses a challenge to confidently identify genes which are truly collaborating with one another. Understanding the functional genomic landscapes of gliomas is therefore of central importance. Genetic screens in mice offer a way to pinpoint functional drivers. Challenges to genetic analysis in normal mouse brains include efficiencies of genome manipulation, cell delivery, and tumor production as well as generating both activating and loss-of-function mutations in a single screen. Conditional *piggybac* mutagenesis is a powerful cancer screening platform that has not previously been applied to central nervous system tumors.

Activating mutations in the epidermal growth factor receptor (*EGFR*) occur in up to 60% of *IDH1* wild-type GBMs [5] of which *EGFRvIII* is the most common (an in-frame deletion of exon 2 to 7 in the extracellular domain leading to constitutive receptor activation [11, 12]). *EGFR* alterations, including amplification, point mutations, and *vIII*, confer similar drug sensitivities to EGFR inhibitors in patient-derived GBM cells [13], and *EGFR* amplification and *EGFRvIII* are retained in most recurrent GBMs (when present in primary tumors) [14], suggesting these alterations have similar functional driving effects in these tumors. Frequent driver mutations and amplifications of *EGFR*, including extrachromosomal ones, have also been detected in *IDH1* wild-type, histologically low-grade gliomas (LGGs) [15, 16]. Some evidence suggests *EGFRvIII* is a late event human GBM: its expression is heterogeneous, and it is found on double minute chromosomes with EGFR inhibitors causing selective pressure to drive its disappearance yet unable to elicit a cure [17]; however, the *EGFRvIII* mutation has also been detected throughout GBMs, including regions with and without its expression, suggesting *EGFRvIII* may be an early event in some cases [18]. Challenges for translational studies include lack of understanding of the cooperative drivers of EGFR and the paucity of *EGFRvIII*-GBM cell lines due to loss of *EGFRvIII* during in vitro culture of primary cells, highlighting the need for relevant models [19].

*EGFR* has been shown to initiate brain gliomas with short latency in mice only when combined with multiple tumor suppressor losses, such as *Cdkn2a* [20–22] and *Pten* [12, 23]. Moreover, *EGFR* amplification and expression have been identified in a human spinal glioma subset (leptomeningeal-disseminated pediatric LGGs [24, 25]). However, the role of *EGFR* mutations in spinal gliomas and their cooperative genetic drivers in brain and spinal tumors remain largely unknown [26].

Here, we show *EGFRvIII* is sufficient to initiate gliomagenesis from the normal mouse brain and spinal cord with long latency. We hypothesized that conditional genome-wide *piggyBac* mutagenesis in the presence of a strong initiating *EGFR* mutation may be a fruitful approach for mapping cooperative glioma driver landscapes in vivo. This novel approach in combination with genomic sequence analysis sheds light on the nature of tumor-genome evolution in genetically engineered mouse models (GEMMs) of glioma. We show these approaches can help decipher the complex genomes of human gliomas and the driver landscapes illuminate potential molecular intervention points for therapeutics.

## Results

### *EGFRvIII* expression initiates progressive gliomagenesis

**Fig. 1:**
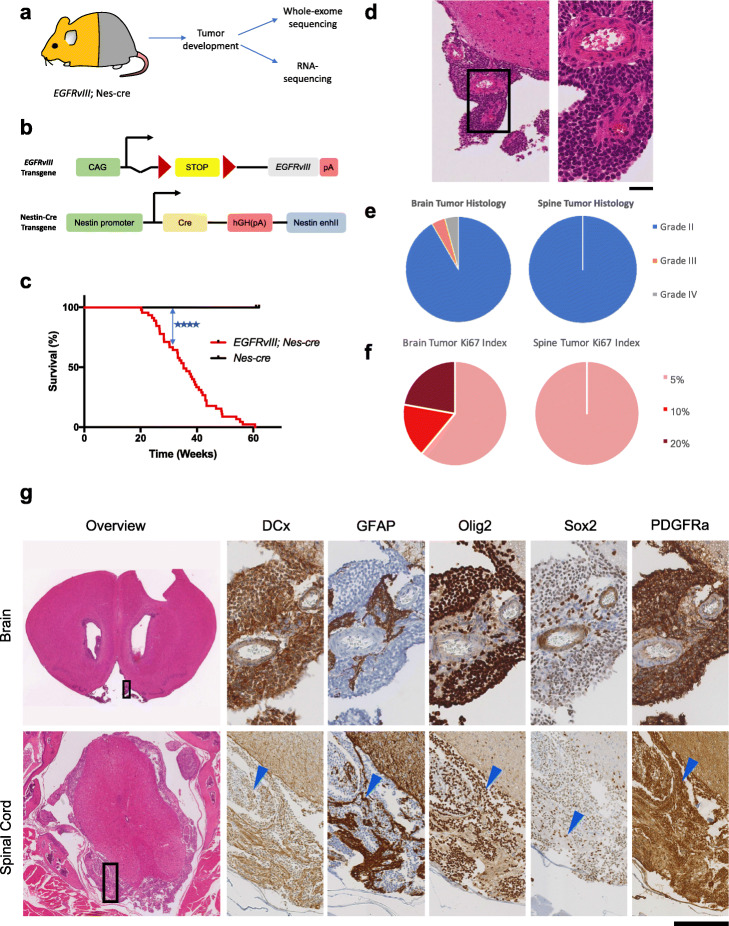
EGFRvIII initiates gliomagenesis in the brain and spinal cord of mice. **a** Outline of the experiment: *EGFRvIII* was conditionally expressed throughout the central nervous system using *nes-cre*. **b** Structures of *EGFRvIII* [12] and *nes*-cre alleles. Notation: CAG = cytomegalovirus (CMV) early enhancer, chicken β-actin promoter; pA = poly-adenylation signal; red triangle = *loxP* site; hGH(pA) = human growth hormone polyadenylation signal; nestin enhII = enhancer in second intron of rat nestin gene [27]. **c** Kaplan-Meier plot of *EGFRvIII*; *nes-cre* mice and control (*nes-cre*) mice (*p* < 0.0001, log-rank test, *n* = 48 and *n* = 10 mice respectively). **d** Low- and high-power views of a small glioma (microneoplasia) protruding from the cortical surface of the brain. Scale bar corresponds to 90 μm for left panel and 25 μm for right panel. **e** Pie charts show proportions of brain and spinal tumors of various grades in heterozygous *EGFRvIII*; *nes*-cre mice. **f** Pie charts show relative proportions of Ki67 proliferative index for brain and spinal tumors of these mice. **g** Top panel (left to right): low-power view of H&E stain of a brain with a typical microneoplasia (same as in **d**), and high-power view of immunostains of this neoplasm showing positivity for neural lineage markers double-cortin (DCx), GFAP (reflecting reactive astrocytes between tumor cells), Olig2, Sox2, and PDGFRa (*n* = 5/5). Histopathologic diagnosis was made by an expert consultant neuro-oncological pathologist. Lower panel (left to right): low-power view of spinal cord with an encasing glioma, and high-power views of immunostains of this tumor for neural lineage markers—tumor cells are negative for DCx, reactive astrocytes are positive for GFAP, and tumor cells are positive for Olig2, Sox2, and PDGFRa. Scale bar corresponds to 1 mm for left H&E panel, 70 μm for upper immunostain panels; 0.7 mm for lower H&E panel, 140 μm for lower immunostain panels

To study the role of mutant *EGFR* in gliomagenesis, we generated double heterozygous mice carrying a conditional human *EGFRvIII* transgene [12] and cre under the control of the Nestin promoter [27] (*nes*-*cre*) which specifically activates *EGFRvIII* expression in the central nervous system, (Fig. 1a, b). *EGFRvIII*; *nes-cre* mice were born at expected frequencies with structurally normal brains. By 60 weeks of age, 100% of mice had succumbed to brain and/or spinal tumors (*n* = 48) (Fig. 1c).

Examination of brains prior to clinically overt disease (mice aged 12–28 weeks) revealed focal cellular expansions in the subventricular zone (SVZ) and small glioma precursors with proliferative activity, also described as “microneoplasias” [28, 29] (12/12 mice, Additional file 1, Fig. S1). Multiple lesions were detected bilaterally protruding into the lateral ventricles and from the brain surface (Fig. 1d). These microneoplasias expressed markers of neural stem cells and transit-amplifying cells, specifically Sox2, Nestin, PDGFRa, GFAP and Olig2, (Fig. 1g, Additional file 1: Fig. S2).

Mice later developed neurological signs due to one or multiple gliomas within the lateral ventricles and/or brain surface with evidence of subarachnoid involvement (40/48 mice had brain gliomas; mean survival 41.1 weeks); immunostaining for human EGFR and EGFRvIII confirmed *EGFRvIII* expression specifically and clonally in tumor cells of microneoplasias and gliomas (Additional file 1: Fig. S3, S4). These tumors had histological features comparable to those of human gliomas, Additional file 1: Fig. S5; a small proportion displayed necrosis and microvascular proliferation, characteristic of GBMs, Additional file 1: Fig. S6. The proportions of grade II, III, and IV tumors and their proliferative indices (Ki67) are shown in Fig. 1e, f and Additional file 1: Fig. S7. A histopathological difference between these mouse and human gliomas however is that *EGFRvIII* is largely found in human GBMs whereas the majority here are mouse LGGs, as observed in some other *EGFRvIII* mouse models [21].

Overall, these results show that *EGFRvIII* can initiate gliomagenesis in the brain, with the long latency reflecting the need for secondary mutations.

### *EGFRvIII* drives spinal cord gliomas

In addition to brain tumors, *EGFRvIII*; *nes-cre* mice also developed multiple and widespread spinal tumors with 100% penetrance (48/48 mice, Additional file 2: Table S1), causing neurological deficits including limb weakness and ataxia. The tumors were located on the spinal cord surface and locally invaded surrounding soft tissue, nerve roots, and cranial nerve ganglia (Additional file 1: Fig. S8). At an advanced stage of tumor progression, tumor cells were seen invading the parenchyma, reminiscent of intramedullary spread of spinal astrocytomas in humans. They were present throughout the leptomeningeal space indicating leptomeningeal spread, a poor prognostic indicator in patients [30]. Tumors showed striking resemblance in histology and location with human leptomeningeal-disseminated spinal gliomas.

In 8/48 mice without established brain tumors (but with microneoplasia), there were widespread spinal tumors. Histology of spinal tumors universally classified them as grade II glioma even in the presence of grade IV intracranial gliomas, suggesting these are primary spinal gliomas, most likely arising independently (Fig. 1e). These spinal tumors expressed classical glioma markers, such as GFAP, Sox2, Olig2, and PDGFRa, and had a lower proliferative index than the corresponding brain tumors (Fig. 1f, g). These *EGFRvIII*-induced spinal tumors represent, to our knowledge, the first mouse model of spinal gliomas with leptomeningeal dissemination.

### Whole-exome sequencing reveals the mutational landscape

**Fig. 2:**
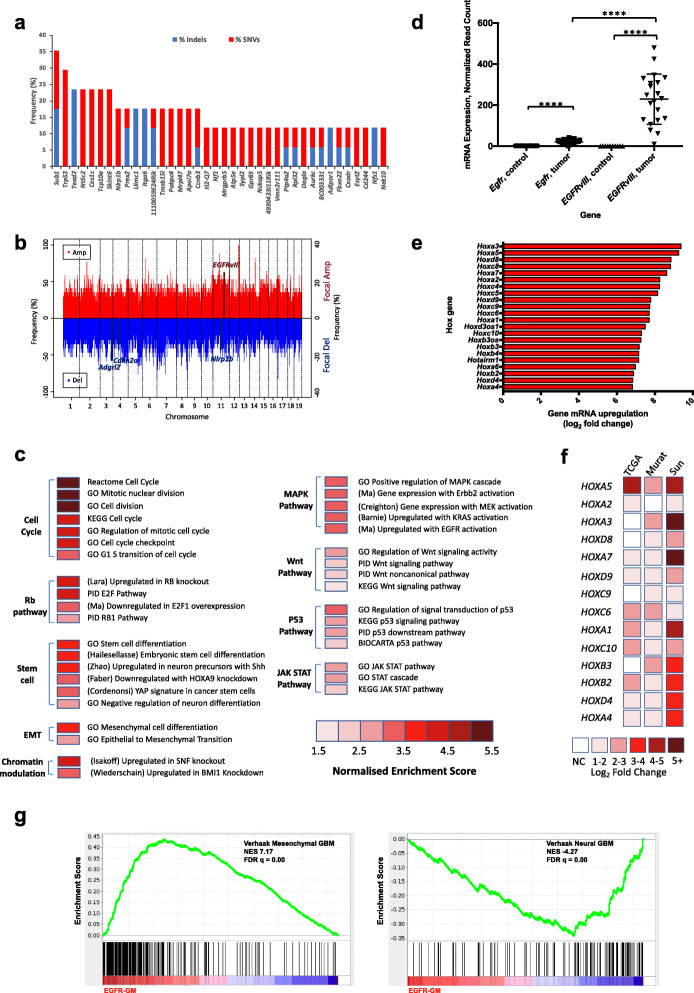
Whole-exome and transcriptome analysis of *EGFR*-mutant mouse brain and spinal cord gliomas. **a** Mutational profile of 17 brain and spinal tumors. Genes are ranked according to the frequency of mutations (indels or SNVs). Known glioma drivers include *Trp53* and *Nf1*, and novel ones found mutated are *Sub1* and *Tead2*. **b** Copy number profile, left axis shows frequency of larger amplifications and deletions, whereas right axis represents frequency of focal copy number changes; key genes with focal alterations are highlighted. **c** Gene set enrichment analysis reveals collaborative pathways in *EGFR*-mutant brain tumors, including oncogenic pathways, stem cell, and epithelial to mesenchymal (EMT)-related pathways. Each line identifies a transcriptomic profile with a Bonferroni-adjusted *p* value < 0.01. Although not displayed here, spinal tumors are enriched for the same pathways implying conserved molecular mechanisms. **d** Plot showing stronger upregulation of *EGFRvIII* mRNA expression (from RNA sequencing) compared with wild-type *Egfr* in tumors, highlighting the former is the more prominent driver (****p < 0.0001, paired *t* test; *n* = 11 brain tumors, *n* = 10 spinal tumors, relative to wild-type brain, *n* = 6, and spinal cord, *n* = 6). Mean expression and standard deviation values are plotted. **e***Hox* gene upregulation in *EGFRvIII-*brain gliomas. Genes are ranked according to log_2_ fold change compared to wild-type brain, Benjamini-Hochberg adjusted *p* < 1 × 10^−12^ for each gene. **f** Heat map showing expression of *HOX* genes in human GBMs relative to normal brain from three datasets (TCGA, Murat and Sun; *n* = 542, 80 and 81 GBMs respectively); log_2_ fold changes are all significant with Benjamini-Hochberg adjusted *p* < 0.05, except for “NC” (“no change”). Genes are ranked according to the greatest upregulation in mouse tumors. Heatmap shows upregulation for *HOX* genes in human GBMs, with no cases of downregulation. **g** Gene set enrichment analysis (GSEA) plots for *EGFRvIII* mouse gliomas showing significant positive enrichment for human mesenchymal GBM and negative enrichment for the neural GBM signature (Verhaak dataset); normalized enrichment score (NES) and FDR *q* value are stated on the plots. There was also weaker positive enrichment for Verhaak human proneural GBM and classical GBM signatures (NES 2.22 and 1.92, FDR *q* value 0.004 and 0.018 respectively)

**Fig. 3:**
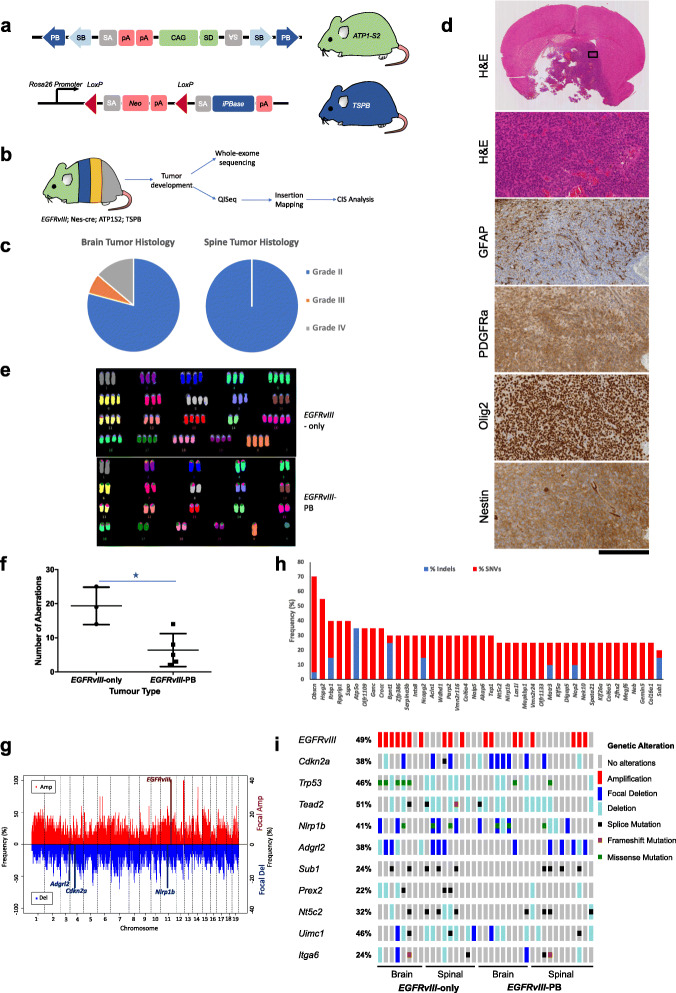
Conditional *piggyBac* transposon mutagenesis substitutes for genomic instability in *EGFRvIII*-mutant gliomas. **a** Mouse constructs for *piggyBac* transposition. The ATP1-S2 transposon line, with 20 copies per cell. Conditional *piggyBac* transposase targeted to *Rosa26* (tissue-specific *piggyBac* transposase, TSPB), SA = splice acceptor; SD = splice donor; CAG = CAG promoter; SB = Sleeping Beauty; PB = *piggyBac* inverted repeats; iPBase = insect version of the *piggyBac* transposase. The transposon can activate gene transcription if it inserts in the same orientation as the gene, usually in a 5′ position. Gene inactivation can occur if the transposon inserts in the body of the gene as a consequence of gene trapping which can occur in either orientation because of the presence of two splice acceptors and bidirectional poly(A) (pA) sites. **b** Outline of the experimental design: quadruple transgenic mice conditionally activate *EGFRvIII* expression and *piggyBac* transposition in the central nervous system. Resultant tumors are examined molecularly by whole-exome sequencing and mapping of transposon insertions. **c** Histology of *EGFRvIII*-PB tumors; although not statistically significant, a higher proportion of grade IV brain tumors are observed compared with tumors lacking transposition. **d** Immunostaining profile of a typical grade III brain glioma from an *EGFRvIII*-PB mouse, showing strong expression of neural stem and transit-amplifying cell markers. Scale bar corresponds to 2.8 mm for top panel, and 200 μm for all other panels. **e** Representative karyotype of *EGFRvIII*-only and *EGFRvIII-*PB brain tumors, showing polyploidy in the non-PB tumor. **f** Chromosomal aberrations in *EGFRvIII*-only and *EGFRvIII*-PB tumors (*n* = 3 and *n* = 5 tumors respectively; mean chromosomal aberrations 19 vs 6.4, *p* = 0.013, unpaired two-tailed *t* test; plots show mean ± standard deviation). **g** Copy number profile of *EGFRvIII*-PB tumors (*n* = 20) with focal amplifications and deletions in key genes highlighted. **h** Mutational profile of 20 *EGFRvIII*-PB brain and spinal tumors from whole-exome sequencing. **i** Key cancer genes identified, either as significantly mutated from MuSiC or copy number altered from GISTIC2, across all mouse brain and spinal tumors in both cohorts; each column represent one tumor

To identify somatic mutations and copy number changes acquired after glioma initiation by *EGFRvIII*, we performed whole-exome sequencing (WES) on 17 tumors (9 brain and 8 spinal gliomas). To increase power for detection of recurrent mutations, WES analysis was performed on the pooled group of gliomas from all CNS compartments [31]. Across all tumors, we found 85 significant recurrently mutated genes with mutations in two or more tumors identified by MuSiC [32] (adapted for mouse data); most had single-nucleotide variants (SNVs) but some genes exhibited INDELS (Fig. 2a, Additional file 3: Table S2). The median number of exonic mutations per tumor was 29 of which missense mutations were the most common. *Sub1*, a transcriptional coactivator, was the most frequently mutated gene (6 mutations in 5/17 tumors, *p* = 1.1 × 10^−16^, FDR 2.27 × 10^−12^, likelihood ratio test, LRT) displaying INDELs and SNVs, all in splice sites suggesting loss of function. *Trp53*, a known tumor suppressor in human LGG and GBM [33], was the second most frequently mutated gene (5/17 tumors had a *Trp53* missense mutation, all within *Trp53*’s DNA-binding domain; *p* = 1.13 × 10^−12^, FDR 7.75 × 10^−9^, LRT; Additional file 1: Fig. S9), validating the application of WES to identify relevant collaborative mutations. Similarly, *Nf1*, a known genetic driver of brain and spinal gliomas [34], was found to be mutated in two tumors (*p* = 0.0010, FDR 0.17, LRT). Other frequently mutated genes were *Tead2*, *Nt5c2*, *Ces1c*, *Prex2*, *Uimc1*, and *Itga6*. *Tead2*, a transcription factor in the Hippo pathway, had recurrent mutations across its TEA/ATTS (DNA-binding) domain (4 mutations in 3/17 tumors; *p* = 2.80 × 10^−11^, FDR 1.15 × 10^−7^, LRT), including splice site mutations and one frameshift mutation, suggesting loss of function. *Uimc1* and *Itga6* had three mutations each (*p* = 1.39 × 10^−7^ and FDR 1.9 × 10^−4^, *p* = 2.7 × 10^−7^ and FDR 3.2 × 10^−4^, LRT, respectively), all of which were INDELS and one of which caused a frameshift in *Itga6* (Fig. 3i). These gliomas were all wild-type for *Idh1*, consistent with gliomas in humans in which *IDH1* and *EGFR* mutations tend to be mutually exclusive.

In contrast to the relatively small number of recurrent mutations, *EGFR*-mutant tumors had complex genomes by DNA copy number analysis (Fig. 2b). Significant focal amplifications and deletions, identified by GISTIC2 [35], were evident in regions with known cancer genes, for example, significant focal *Cdkn2a* deletions (GISTIC *q* value = 1.39 × 10^−5^) were evident and *EGFRvIII* (in *Col1a1* locus, GISTIC *q* value = 0.017) was recurrently amplified. Significantly recurrent focal deletions were present in a novel putative glioma driver *Adgrl2* (GISTIC *q* value = 2.19 × 10^−6^, Additional file 4: Table S3). Several of the most significantly mutated genes were also in regions with frequent deletions, including *Trp53*, *Tead2*, and *Uimc1*, supporting putative tumor suppressive roles (Fig. 3i).

The significance and translational relevance of the most frequently mutated and/or focally deleted genes detected in mouse gliomas were assessed by comparison with human glioma datasets from The Cancer Genome Atlas (TCGA; *n* = 283 LGGs, 273 GBMs) [36, 37]. This revealed that *TEAD2* is recurrently deleted in 48% of human LGGs in a mutually exclusive manner with *TP53* (Bonferroni-adjusted *p* < 0.001, Fisher’s exact test, Additional file 1: Fig. S10). Recurrent deletions in previously unknown glioma genes *NT5C2*, *ADGRL2*, and *UIMC1* were observed whilst *SUB1*, *CES1*, and *ITGA6* were frequently methylated in human LGGs (Additional file 1: Fig. S10); frequent CNVs in these genes were also present in human GBMs. Subgroup analysis confirmed recurrent mutations/CNVs (> 2 tumors) in these genes specifically in *EGFR*-mutated/amplified human LGGs and GBMs. These data cross-validate the relevance of these novel putative drivers in humans.

### Transcriptomic profiling defines glioma oncogenic pathways

To delineate the signaling pathways deregulated in tumors, we performed RNA-sequencing (RNA-seq) on 11 *EGFRvIII*-expressing mouse brain gliomas and 10 spinal gliomas.

Compared with normal mouse brains (*n* = 6), *EGFRvIII*-brain gliomas show 2000 upregulated and 1784 downregulated genes (log_2_ fold change > ± 2 and Benjamini-Hochberg adjusted *p* value < 0.01, Additional file 5: Table S4). Gene ontology (GO) analysis of upregulated genes showed a significant enrichment for genes related to the cell cycle and mitosis, differentiation, and neurogenesis (FDR < 0.001). Downregulated genes showed enrichment for pathways such as neuron differentiation and migration (FDR < 0.001). Gene set enrichment analysis (GSEA) of differentially expressed genes in *EGFRvIII*-brain gliomas showed significantly enriched gene sets (*p* < 0.01) including p53, Wnt, MAPK, Jak-Stat, Rb pathways, and stemness, implicating these oncogenic pathways in driving gliomagenesis in cooperation with *EGFRvIII* (Fig. 2c, Additional file 1: Fig. S11).

The most “upregulated” gene was the *EGFRvIII* transgene, but as this human transgene is not present in normal tissue, fold change is not meaningful. The endogenous *Egfr* gene was also upregulated (mean log_2_ fold change = 3.71) in both brain and spinal tumors, suggesting both mutant *EGFR* and wild-type *Egfr* expression are advantageous to tumor growth (Fig. 2d), consistent with previous reports suggesting collaboration between the two as observed in human GBMs [38]. The majority of the top mutated genes are also expressed, including *Sub1*, *Trp53*, *Tead2*, *Nt5c2*, *Prex2*, *Uimc1*, and *Itga6*.

*Hox* (homeobox) genes have been implicated in escape from apoptosis, epithelial-mesenchymal transition, and angiogenesis in other cancers [39]. Nineteen of the 30 most strongly upregulated genes in the brain tumors were *Hox* genes (Benjamini-Hochberg adjusted *p* < 1 × 10^−12^, Fig. 2e), and these top genes associate with patient survival from human GBM TCGA data (Additional file 1: Fig. S12). Comparative analysis with large human GBM (Sun, Murat, and TCGA [37, 40, 41]) datasets revealed 14 of these most upregulated *Hox* genes in mice are also upregulated in human tumors, supporting a proposed role in oncogenesis [42, 43], Fig. 2f. In contrast, spinal tumors did not show such strong upregulation of *Hox* genes, although they did exhibit enrichment for the other oncogenic pathways (*p* < 0.01, Additional file 1: Fig. S13, S14, Additional file 6: Table S5).

Human gliomas may be classified according to gene expression profiles [44]. Comparison with human glioma subsets (Verhaak dataset) using GSEA revealed these mouse tumors showed strongest enrichment for the human mesenchymal GBM signature (*q* value < 0.01), although there was also weaker enrichment for the proneural and classical GBM signatures and negative enrichment for the neural GBM signature, Fig. 2g. Therefore, these tumors recapitulate key molecular features of a clinically relevant human GBM subset.

### Transposon mutagenesis replaces genomic instability in glioma progression

Transposons have been successfully used for identifying cancer driver genes [45–53]. Mobilized *piggyBac* transposons randomly integrate in the genome and activate and/or disrupt gene expression [54]. Given large chromosomal aberrations or transcriptional changes make pinpointing driver genes difficult to identify, we performed a conditional *piggyBac* transposon mutagenesis screen in vivo to further identify genes that cooperate with mutant *EGFR* in gliomagenesis.

To limit transposition to the central nervous system, a conditional *piggyBac* transposase allele was activated by *nes-cre* (Fig. 3a, b). An experimental cohort of quadruple transgenic mice carrying conditional *EGFRvIII*, 20 copies of a *piggyBac* transposon (ATP1S2) [54], a conditional *piggyBac* transposase, and *nes-cre* were generated (*EGFRvIII*-PB, *n* = 72; Fig. 3b, see Methods). As controls, we established transgenic mice expressing *EGFRvIII* but lacking transposition (*EGFRvIII*; *nes-cre* = *EGFRvIII*-only, *n* = 48) and a set with transposition but lacking *EGFRvIII* (transposase; ATP1S2; *nes-cre* = PB-only, *n* = 20). Mean survival times between *EGFRvIII*-PB and *EGFRvIII*-only cohorts were similar (41.4 vs 41.1 weeks, *p* = 0.95, log-rank test), and both groups had similar incidences of brain and spinal gliomas (Fig. 3c, d, Additional file 1: Fig. S15, S16, S17). There was a trend towards increased GBMs in *EGFRvIII*-PB mice compared with *EGFRvIII*-only mice (13.9% vs 4.2% GBMs respectively; *p* = 0.082, two-sided chi-square test).

Genomic instability is a hallmark of cancer (including human gliomas) and a key driving force [55–58]. *EGFRvIII* has also been associated with genomic instability in vitro [59]. We hypothesized that the absence of reduced survival times of *EGFRvIII*-PB mice may reflect genomic instability providing secondary molecular alterations in *EGFRvIII*-only mice that is similar in consequence to transposon mutagenesis in *EGFRvIII*-PB mice. Supporting this, cytogenetic analysis revealed significantly more chromosomal aberrations in *EGFRvIII*-only compared to *EGFRvIII*-PB tumors (19 vs 6.4 mean number of chromosomal aberrations, *p* = 0.013, unpaired two-tailed *t* test; Fig. 3e, f). Whole-exome sequence of 20 brain and spinal gliomas from *EGFRvIII*-PB mice confirmed these had substantially less complex tumor-genomes with fewer copy number changes than *EGFRvIII*-only tumors (Fig. 3g). Nevertheless, whole chromosome 11 amplification was still common as well as focal amplifications of *EGFRvIII* (*Col1a1* locus) and localized deletions in *Cdkn2a* and *Adgrl2* in tumors arising from both cohorts. GISTIC2 analysis shows these alterations occur significantly more frequently than expected by chance (*q* value < 0.05; Additional file 1: Fig. S18, Additional file 4: Table S3), suggesting they provide a selective advantage for tumor progression.

Whole-exome sequence analysis revealed that while the median number of mutations was similar between the cohorts, their mutational profiles differed substantially. The top mutated genes identified in the *EGFRvIII*-PB tumors were *Obscn*, *Hspg2*, *Rrbp1*, *Rpgrip1*, and *Atp5o* which have unknown functions in cancer (Fig. 3h). Although the frequency of mutations in these genes was high (70–40%), *Obscn* and *Hspg2* are particularly large genes (more likely to harbor mutations) and contained many synonymous changes, suggesting they were passengers. Nevertheless, in *EGFRvIII*-PB mice, there were low-frequency mutations in a subset of putative drivers we previously identified in *EGFRvIII*-only tumors, including frequent splice site mutations in *Sub1* and *Nt5c2*, and mutations in *Trp53*, *Tead2*, *Uimc1*, and *Itga6* (Fig. 3i).

We hypothesized genomic instability may be generated through oncogene-induced replicative stress [60]. We studied H2AX phosphorylation by immunostaining, which marks sites of DNA damage (focal nuclear staining) and replication stress (pan-nuclear staining) [61]. Mouse *EGFRvIII*-GBMs displayed large areas with a substantial fraction of cells showing intense pan-nuclear γ-H2AX and others with γ-H2AX foci (Additional file 1: Fig. S19a, b). Gene set enrichment analysis of RNA-seq data from these tumors revealed significant enrichment for upregulated gene sets involved in DNA repair, double strand break repair, base excision repair, and DNA damage checkpoints (Additional file 1: Fig. S19c). Specific DNA repair genes significantly upregulated in these tumors include *Chek2*, *Xrcc2*, *Xrcc4*, *Ercc2*, and *Foxm1*. These data are consistent with a model of oncogene-induced replication stress leading to genomic instability and activation of the DNA damage response (DDR), previously proposed for other oncogenes such as *K-ras* [62].

Together, these results suggest that *piggyBac* mutagenesis substitutes for genomic instability and highlight the relevance of transposon-mediated mutations for gliomagenesis. Replacing large chromosomal anomalies with precise genetic hits enables functional genomic interpretation.

### Transposon mutagenesis identifies *EGFR*-mutant glioma driver landscape

**Fig. 4:**
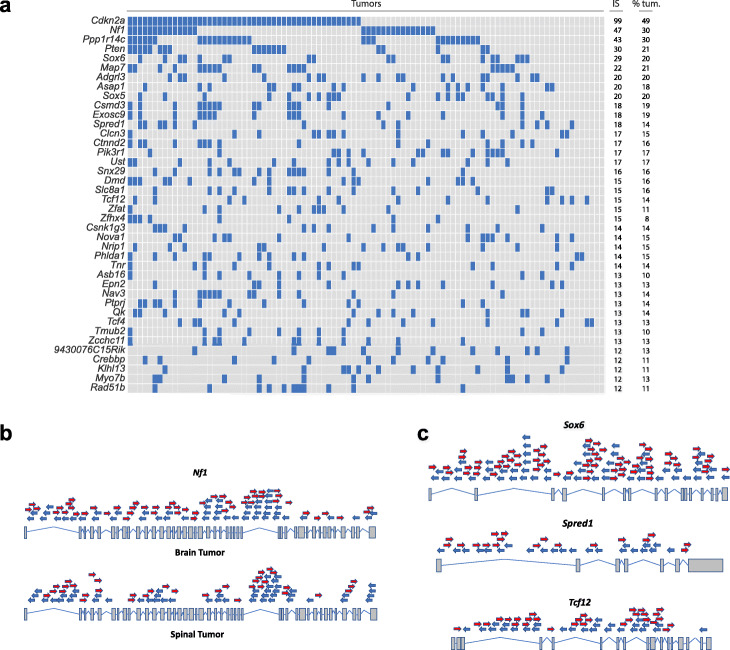
*PiggyBac* transposition identifies 281 known and novel driver genes cooperating with mutant *EGFR* in brain and spinal gliomas. **a** Oncoprint showing the top CIS genes across all 96 brain and spinal gliomas (Bonferroni adjusted *p* < 0.01 for each gene, Gaussian kernel convolution analysis). “IS,” total number of insertion sites; “% tum.,” percentage of tumors with an insertion in corresponding gene. The most well-known brain glioma tumor suppressors are among the top 4 genes (*Cdkn2a*, *Nf1*, and *Pten*). Novel glioma genes include *Sox6*, *Spred1*, and *Tcf12*. **b** The position of all transposon insertions across *Nf1* (a known brain tumor driver) in brain and spinal gliomas, showing a gene-disruption insertion pattern. Blue arrow = antisense orientation; red arrow = sense orientation with respect to gene direction. **c**. Novel glioma drivers, *Sox6*, *Spred1*, and *Tcf12* also have disruptive insertional patterns. These figures show all *piggyBac* insertions in brain tumors

To identify the genetic driver landscape with *piggyBac*, common integration sites (CIS—genes into which the *piggyBac* transposon has recurrently inserted more frequently than expected by chance, *p* < 0.01) were identified by transposon-host PCR [63] and sequence analysis (quantitative insertion site sequencing, QI-seq). Gaussian kernel convolution was used to identify CIS from 46 brain and 50 spinal tumors [50]. Brain and spinal tumors from the same mice had different transposon integration sites, confirming these tumors arose independently. In total, 281 significant CIS genes were ranked according to the number of insertions across all tumors (Fig. 4a, Additional file 7: Table S6). Pathway analysis using Gene Ontology and Panther [64] revealed that CIS genes were enriched for oncogenic pathways including Ras-MAPK, Wnt, PI3K-AKT, and stem cell-related pathways (Additional file 1: Fig. S20). Analysis of CIS genes with STRING [65] showed PB mutagenesis significantly enriched for mutations that affect a functionally interacting network of proteins in gliomagenesis (Benjamini-Hochberg adjusted *p* = 4.9 × 10^−13^, hypergeometric test, Additional file 1: Fig. S21).

The highest-ranked CIS was *Cdkn2a*, followed by *Nf1 (*Fig. 4b). Loss-of-function mutations of *CDKN2A* and *NF1* have been observed as drivers in a range of human gliomas including LGG and GBM [66, 67]. Interestingly, *Spred1* (whose product also acts as negative regulator of the Ras pathway [68] and is a recently discovered melanoma tumor suppressor [69]) ranked within the top 10 CIS and exhibited a disruptive *piggyBac* insertional pattern, suggesting *Spred1* acts as a novel tumor suppressor in glioma (Fig. 4c). Other MAPK signaling-related genes with recurrent mutations include *Prkca*, *Pebp4*, and *Map3k1*.

Genes involved in the PI3K-AKT oncogenic pathway were also identified including known tumor suppressor genes in gliomas such as *Pten* [70] and *Pi3kr1* [71] as well as novel genes including *Prex2*, and the protein tyrosine phosphatases *Ptpro* and *Ptprj*, all with inactivating transposon insertional patterns. The glioma oncogene and PI3K-AKT activator, *Pdgfrα* [72], was also a CIS, with an insertional pattern consistent with gene activation (Additional file 1: Fig. S22). This supports the validity of our transposon screen in identifying both tumor suppressor genes and oncogenes. Other genes involved in the PI3K pathway with recurrent insertional mutations include *Cbl* and *Pik3c3*.

Several top CIS genes known from their function in nervous system development were not previously recognized as tumor suppressors. *Sox6* and its paralog, *Sox5*, are expressed in a mutually exclusive pattern during brain development [73]—both were identified as CIS. *Tcf12* and *Tcf4*, transcription factors implicated in neurogenesis [74], were also identified as CIS. *Nav3*, a gene belonging to the neuron navigator family predominantly expressed in the nervous system, had recurrent insertional mutations too. *NAV3* silencing in breast cancer cells increased tumorigenicity in a xenograft model, supporting our data here for gliomas [75]. Inactivating transposon insertion patterns suggest tumor suppressor roles for these genes (Fig. 4c). Frequent insertional mutations were also observed in other genes with developmental roles: *Qki*, *Zeb2*, *Dmd*, *Zfhx3*, *Zfhx4*, and *Exosc9*.

To explore the evolutionary mechanisms underlying brain gliomas in our mouse model, we performed multi-region tumor sampling and QI-seq. This revealed intratumor heterogeneity, with clonal and subclonal *piggyBac* insertions, implying branching tumor evolution (Additional file 1: Fig. S23). With the exception of clonal *Pdgfra* and *Nav3* insertions in one tumor, transposon insertions in MAPK/PI3K pathway and neurodevelopmental genes (including *Nf1*, *Pten*, *Pik3r1*, *Ptprj*, *Sox6*, *Sox5*, and *Tcf4*) were subclonal in these tumors, implying these were late evolutionary events. Altogether, *piggyBac* mutagenesis has comprehensively identified known and novel putative cancer genes and pathways driving *EGFR*-mutant gliomas.

### Comparative validation of CIS genes with human TCGA gliomas

**Fig. 5:**
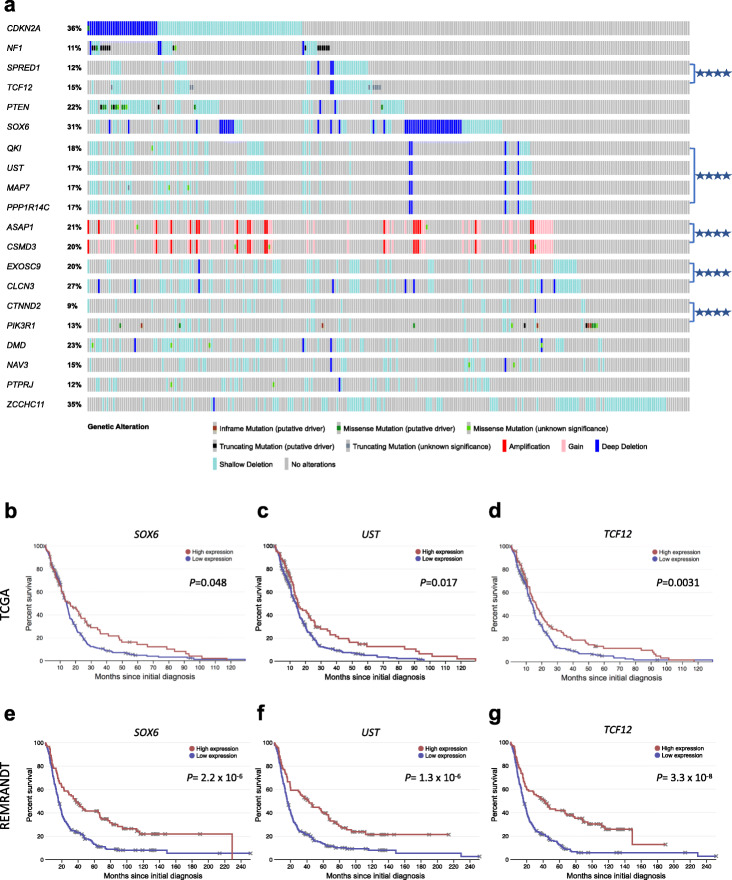
Top *piggyBac* CIS genes are recurrently altered in human brain gliomas. **a** Patient data was analyzed from The Cancer Genome Atlas (TCGA) datasets (*n* = 283 LGGs, 273 GBMs; LGG data shown here, GBM data shown in Additional file 1: Fig. S24), for cross-comparison of the main CIS genes in mouse brain and spinal tumors. The frequency of alterations of CIS genes observed in patient samples is indicated. Similar genes (*NF1* and *SPRED1*) and co-deleted/co-amplified genes have been grouped together. *TCF12* and *SPRED1* are co-deleted (chromosome 15q), as are *QKI*, *UST*, *PPP1R14C*, and *MAP7* (chromosome 6p), as well as *EXOSC9* and *CLCN3* (chromosome 4q). *ASAP1* and *CSMD3* (chromosome 8q) are co-amplified in human tumors. From these 20 top CIS genes, there are 28 gene pairs with significantly co-occurring alterations in human LGGs, many of which are on neighboring chromosomal locations; 8 pairs had mutually exclusive alterations (Bonferroni-corrected *p* value < 0.05, Fisher’s exact test); for simplicity, only the key co-occurring alterations are highlighted here. **b**–**e** Kaplan-Meier plots of GBM patient survival in relation to expression levels of key mutated novel genes *SOX6* (**b**), *UST* (**c**), and *TCF12* (**d**) in TCGA and REMBRANDT (**e**–**g**) datasets. *P* values were calculated using the log-rank test comparing the top 30% of expression level with the lower 70% for each gene. All survival data from TCGA and REMBRANDT GBM datasets were used (*n* = 348 and *n* = 329 patients respectively with survival data), to ensure a sufficient sample size; analyses were performed using the open web interface “Project Betastasis” (www.betastasis.com)

To assess the clinical relevance of the putative glioma driver genes, we analyzed the frequency with which genetic alterations occur in our top CIS genes in 283 human brain LGGs and 273 GBMs from TCGA datasets [36, 37]. Aside from the known brain glioma tumor suppressors, *CDKN2A*, *NF1*, and *PTEN*, we found *SPRED1* is deleted (heterozygous or homozygous) in 12% of LGGs and 27% of GBMs; and *TCF12* deletions and/or truncating mutations are present in 15% of LGGs and 23% of GBMs—indeed *SPRED1* and *TCF12* are mostly co-deleted (*p* < 0.001, Fisher’s exact test) likely as part of a 15q deletion [76]. *SOX6* is deleted with high frequency: 31% of LGGs and 18% of GBMs, Fig. 5a and Additional file 1: Fig. S23. Subgroup analysis confirmed these top CIS genes had recurrent mutations/CNVs (> 2 tumors) in *EGFR*-mutated/amplified human LGGs and GBMs.

*QKI*, *UST*, *PPP1R14C*, and *MAP7*, all mapping to chromosome 6q, are frequently co-deleted in both human LGGs and GBMs (Bonferroni-adjusted *p* < 0.001, Fisher’s exact test; Fig. 5a, Additional file 1: Fig. S24). In mice, all four genes had recurrent *piggyBac* insertions across their sequence (implying gene disruption), supporting the hypothesis that there are multiple putative tumor suppressors in this region [77]. Similarly, *EXOSC9* and *CLCN3* are frequently co-deleted on human chromosome 4q and both had disruptive transposon insertions in mice. These data illustrate the potential utility of *piggyBac* in pinpointing cancer drivers hidden within large copy number altered regions.

To understand the clinical relevance of top mutated novel genes, we analyzed the REMBRANDT [78] and TCGA GBM datasets for correlation of gene expression with patient survival (*n* = 329 and *n* = 348 tumor samples respectively): expression levels of *SOX6*, *UST*, *QKI*, *PPP1R14C*, *TCF12*, *SPRED1*, *TEAD2*, and *NAV3* significantly correlated with patient survival in one or both of these independent datasets (*p* < 0.05, log-rank test, comparing patients with upper 30% vs lower 70% of expression levels, Fig. 5b–g). Moreover, deletions in these genes associate with correspondingly lower gene expression (Additional file 1: Figure, S25). Altogether, these results further support roles for these genes in gliomagenesis.

### Validating the effects of transposon insertions from glioma transcriptomes

**Fig. 6:**
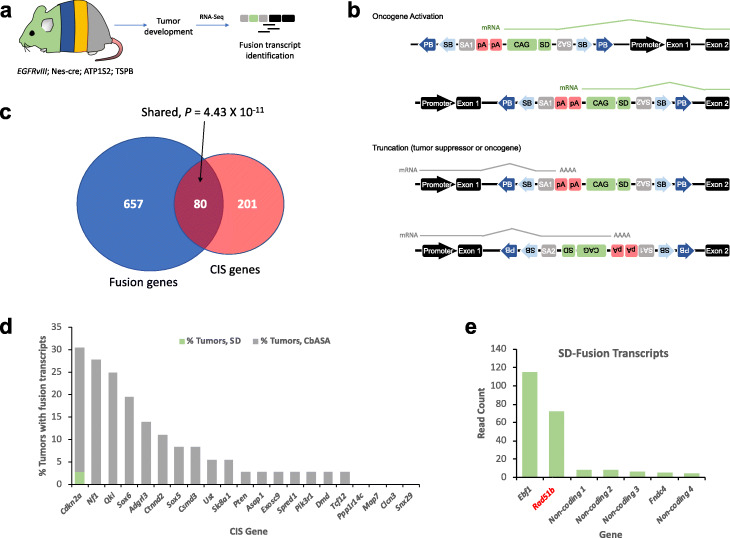
Effects of PB insertions on glioma transcriptomes. **a** RNA-seq was performed on tumors from *EGFRvIII*-PB mice (*n* = 36), with IM-Fusion [80] analysis of the data to identify fusion transcripts. **b** Overview of the effect ATP1-S2 transposons on the transcriptome: the transposon can insert in the sense orientation upstream of a gene’s promoter or in an early intron, driving gene transcription through the transposon’s promoter and splice donor (SD). Alternatively, it can cause transcript termination by inserting in an intron in either sense or antisense orientation because of its two splice acceptors (SA1 = CbASA; SA2 = En2SA) and bi-directional polyA sites; transcript termination can have the effect of inactivating tumor suppressor genes, but also potentially activating an oncogene if there are downstream inhibitory domains for the protein that are removed. **c** Of all genes with fusion transcripts, 80 genes overlapped with CIS genes identified by QI-seq. *P* value was calculated using a two-sided Fisher’s exact test. All fusion transcripts detected with the carp-beta-actin splice acceptor (CbASA) and splice donor (SD) contained *piggyBac* in the sense orientation, and all those with Engrailed-2 exon-2 splice acceptor (En2SA) contained *piggyBac* in the antisense orientation, suggesting the transposon insertion had functional consequences in all cases. **d** Bar plot showing percentage of gliomas with fusion transcripts among top 20 CIS genes (*Qki* is also included here). **e** Top fusion transcripts containing the PB splice donor ranked by read count; among them, only *Rad51b* was also identified as a CIS gene

Transposition results in fusions with endogenous genes that can be detected by RNA-seq [79]. To produce direct evidence of *piggyBac* insertions affecting transcription of target CIS genes, we performed paired-end RNA-sequencing of 36 brain and spinal gliomas from *EGFRvIII*-PB mice and implemented IM-Fusion to detect gene-*piggyBac* fusion transcripts [80], Fig. 6a, b. Of the 281 CIS genes identified by QI-seq, 80 had supporting fusion transcripts from RNA-seq analysis (4.43 × 10^−11^, two-sided Fisher’s exact test, Fig. 6c). Moreover, 16 of the top 20 CIS genes had supporting fusion transcripts from at least one tumor, including *Cdkn2a*, *Nf1*, *Pten*, *Sox6*, *Sox5*, *Spred1*, and *Tcf12* (all containing PB splice acceptor fusions, implying transcript termination; Fig. 6d). Other key genes with fusion transcripts suggesting disruption included *Qki* and *Ust* (Additional file 8: Table S7). Of the genes with the most fusion transcript sequencing reads containing PB splice donor (implying activating insertions, see Fig. 6b), *Rad51b* was also a CIS gene (Fig. 6e); its fusion transcripts found in two tumors imply a putative oncogenic role, supporting data demonstrating *RAD51* inhibition radio-sensitizes gliomas by reducing DNA repair [81]. These transcriptomic signatures of *piggyBac* support the functional effects of the identified CIS genes on gliomas.

### Brain and spinal tumors share core genetic drivers

**Fig. 7:**
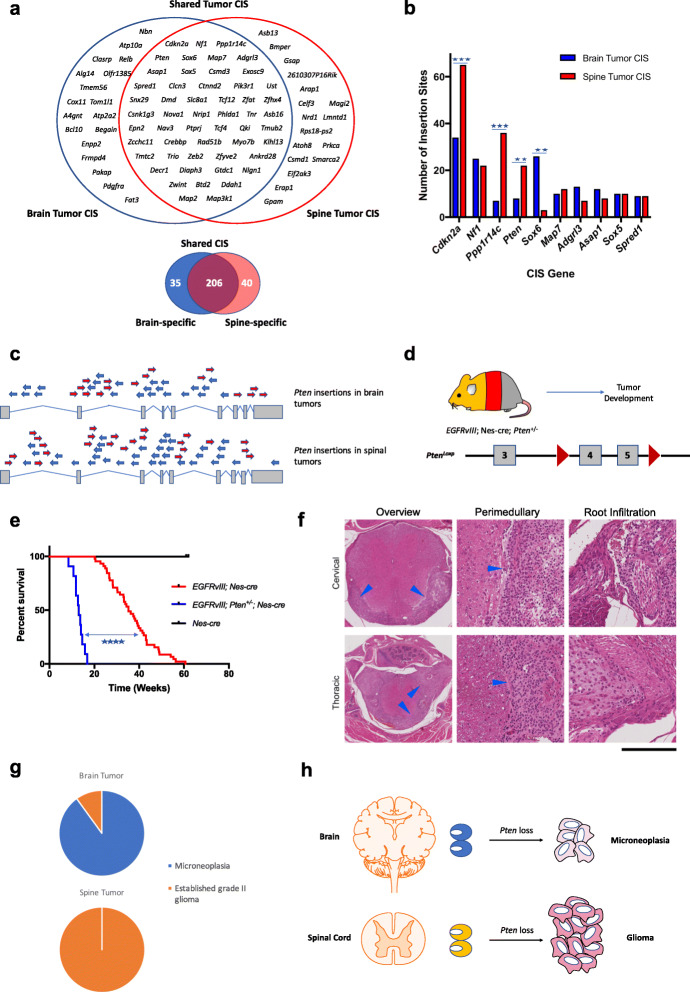
Novel context-dependent differential tumor suppressive effects of *Pten* in the brain and spinal cord. **a** Brain and spinal gliomas share a core set of drivers. Upper Venn diagram shows the top genes from each tumor cohort, with core drivers including genes such as *Cdkn2a*, *Pten*, and *Sox6*. Lower Venn diagram shows among all transposon CIS genes, brain and spinal cord tumors share 206 genes (with at least one insertion in each tumor type), and there are 35 brain glioma-specific CIS genes and 40 spinal glioma-specific CIS genes. **b** Bar plot comparing number of insertions between brain and spinal tumors for the top 10 CIS genes. *Cdkn2a*, *Ppp1r14c*, and *Pten* have significantly more insertions (normalized for number of tumors analyzed) in spinal than brain tumors, and *Sox6* has more insertions in brain tumors (Fisher’s exact test, *p* < 0.05). **c** All *Pten piggyBac* insertions from brain gliomas and spinal cord gliomas are plotted across the structure of the gene, with the pattern implying disruption; note the higher density of insertions in this gene in spinal cord tumors. **d** Conditional mice with both *EGFRvIII* and *Pten* heterozygous loss (exons 4 and 5 deleted with cre [82]) were generated and monitored for brain and spinal tumor development. **e***Pten* loss significantly shortened survival time of mice in the context of conditional *EGFRvIII* expression (*p* < 0.0001, log-rank test, *n* = 11 *EGFRvIII*; *nes*-cre; *Pten*^+/−^ mice and *n* = 48 *EGFRvIII*; *nes*-cre mice). **f***EGFRvIII*; *nes*-cre; *Pten*^+/−^ spinal tumor growth and nerve root invasion. Left panels: cervical and thoracic spinal cord with encasement by tumor cells growing within the subarachnoid space. Middle panels: detailed view of the spinal cord and tumor cells. Right panels: tumor cells invading root structures. Scale bar corresponds to 0.8 mm for left upper panel and 1.6 mm for left lower panel, and 180 μm for all other panels. **g** Histological assessment of *EGFRvIII*; *nes*-cre; *Pten*^+/−^ tumors. In the brain, lesions were predominantly microneoplasias (tumor precursors) rather than fully formed gliomas; in contrast, in the spinal cord, gliomas were uniformly fully established and grade II. **h** Illustration showing the tumor tissue-of-origin in the CNS influences *Pten* tumor suppressive effect strengths

Of the 281 CIS genes, 206 (73%) were shared by both brain and spinal tumors, Fig. 7a. These include known tumor suppressors underlying multiple types of human gliomas, such as *Cdkn2a*, *Nf1*, and *Pik3r1*, as well as several putative tumor suppressors such as *Sox6*, *Tcf12*, and *Spred1*. However, the frequency of insertions in particular shared genes differed between brain and spinal tumors. For example, *Pten* had significantly more insertions in spinal than brain tumors (22 vs 8 insertions respectively, *p* = 0.008, Fisher’s exact test). Conversely, *Sox6* has significantly more insertions in brain compared with spinal tumors (26 vs 3 insertions, respectively, *p* < 0.0001, Fisher’s exact test; Fig. 7b and Additional file 1: Fig. S26). Other CIS occurred uniquely in each tumor type, for example, *Pdgfra* had activating insertions in brain but not spinal tumors (4 and 0 insertions, respectively). Although CIS genes with lower frequency insertions require further characterization to confirm their tumor-type specificity, collectively, these results show there is a shared core set of driver genes for both brain and spinal gliomas.

### Differential tumor suppressive effects of *Pten* in brain and spinal gliomas

*PTEN* loss is a common event and known to cooperate with *EGFR* in brain gliomas but its role is unclear in spinal tumors [83], with no previous mouse models (to our knowledge) showing whether *Pten* drives spinal gliomas. *Pten* was a CIS in both brain and spinal gliomas, Fig. 7c. To explore the role of *Pten* inactivation on brain compared with spinal gliomagenesis, we generated triple transgenic mice carrying the conditional allele of *EGFRvIII*, *nes-cre* and a conditional knockout *Pten* allele [82], *Pten*^*Loxp*^*/+* (*n* = 11; Fig. 7d).

*EGFRvIII*; *nes*-cre; *Pten*^+/−^ mice developed signs of spinal (focal paralysis) rather than brain disease (hydrocephalus or seizures) and showed a reduction in survival time compared with mice just carrying the *EGFRvIII* and *nes-cre* alleles (median age 13.0 vs 41.1 weeks, *p* < 0.001, log-rank test; Fig. 7e). Histological examination of *EGFRvIII*; *nes*-cre; *Pten*^+/−^ mice identified extensive grade II gliomas surrounding the spinal cord at all levels with widespread leptomeningeal and nerve root invasion (9/9) (Fig. 7f, g). Of lesser clinical significance, microneoplasias in the SVZ and base of brain were observed. These data identify *Pten* as a novel spinal glioma tumor suppressor and suggest a stronger cooperative driving effect of *Pten* loss on spinal compared with brain tumors, highlighting context-dependent tumor suppressive effects (Fig. 7h).

### Druggable targets in the glioma driver network

Knowledge of cancer driver landscapes presents opportunities for therapeutic strategies. Using canSAR [84], we have applied established chemogenomic technologies to pharmacologically annotate the glioma set of putative driver proteins identified here. This set was derived from all CIS genes (*EGFRvIII*-PB cohort) and from recurrent significantly mutated genes (*EGFRvIII*-only cohort); given loss-of-function (LOF) of several proteins identified directly lead to Akt activation (e.g., Pten, Ptpro, Pik3r1) [85–87], and Ras/Erk/Mek activation (e.g., Pdgfra, Nf1, Spred1) [88–90], these linked downstream oncoproteins were included as targets. The glioma set thus comprised 375 proteins. Each protein was assessed in multiple ways for “druggability” (probability of the protein being targeted by small molecule drugs). Comparative genomic analysis with human LGG and GBM data from TCGA confirmed all druggable genes in the set, except *Ddx3y* and *Usp9y*, are genetically altered in patients. CanSAR analysis revealed a highly druggable network of putative drivers, with 14 targets of approved drugs (for other indications), 3 targets of clinical investigational drugs, 34 targets under drug discovery or chemical biology investigation, and 96 proteins predicted to be druggable and thus of potential interest for future drug discovery efforts. In addition to targeted EGFR therapies, the network highlights targets being investigated clinically for glioma treatment, including not only PI3K, but also ESR1 and PDGFRA, Additional file 9: Table S8 and Additional file 10: Table S9.

Next, to validate the potential therapeutic effects of targeting these proteins with drugs, we analyzed large-scale drug sensitivity data from 21 human glioma cell lines (including *EGFR*-mutated and wild-type; 13 GBM, 8 LGG; GDSC [91]). Twenty-four drugs acting on our glioma network were tested by GDSC, of which 9 demonstrated significant growth inhibitory effects (IC_50_*Z* score < − 2) and 15 showed partial inhibitory activity on at least one cell line, Additional file 11: Table S10. These results highlight potential efficacy of drugs targeting PI3K, AKT, MEK, ERK, EGFR, and PDGFRA, as well as APP, ESR1, SMARCA2, HDAC9, AURKC, and NAMPT in selected gliomas. Further testing is additionally required in genetically faithful models for drug sensitivity, but blood-brain barrier penetration is a challenge that will need to be overcome to realize the clinical potential of these observations. Nevertheless, such orthogonal demonstrations of functional genes and targets are essential for prioritizing potential therapeutics for preclinical and clinical trials.

### *EGFRvIII*-glioma cells can serially engraft in recipient mice and are suppressed by afatinib

**Fig. 8:**
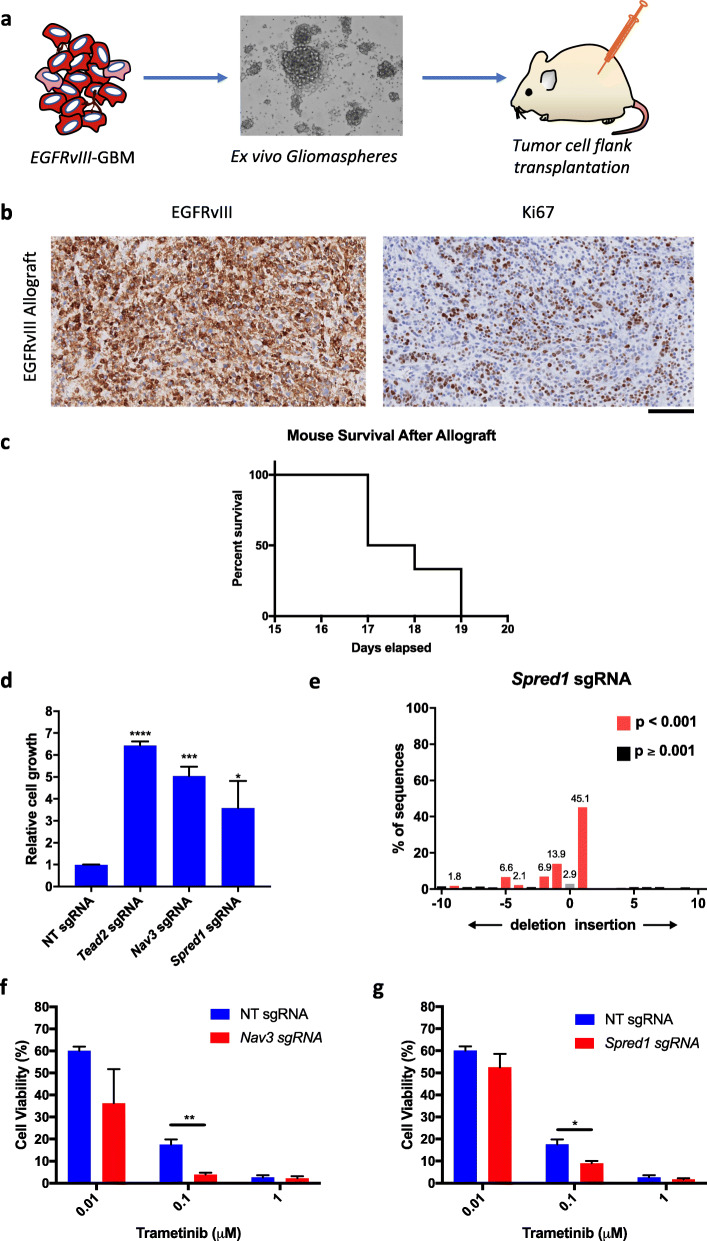
Validation of putative tumor suppressors using an ex vivo glioma model derived from *EGFRvIII*-mouse GBMs. **a** Cells from mouse GBMs were propagated as gliomaspheres in culture and subcutaneously transplanted into NOD-SCID-γ mice. **b** Histology of allografted cells confirmed tumor formation with diffuse expression of EGFRvIII in tumor cells (left panel), and a high level of Ki67 expression (right panel). Scale bar = 100 μm **c** Kaplan-Meier curve of survival of mice with allografts of EGFRvIII-tumor cells; 6/6 transplanted mice developed tumors with a median survival of 17 days. **d** Bar plot showing cell growth 4 weeks after CRISPR-cas9 induced mutations in *Tead2*, *Nav3*, and *Spred1* in EGFRvIII-glioma cells, demonstrating significantly increased viable cells relative to wild-type cells (with non-targeting, NT, sgRNA). Measurements were performed in triplicate with three independent experiments. Data are represented as mean values ± SEM. Significance was determined using the one-way ANOVA test. **e** TIDE confirms a high level of gene editing of *Spred1* using CRISPR-cas9 in glioma cells. **f** Treatment of *EGFRvIII*-glioma cells carrying CRISPR-induced *Nav3* mutations and Spred1 mutations (**g**) with trametinib shows significantly reduced cell viability compared with WT cells (carrying NT sgRNA) at 0.1 μM. Significance was determined using the two-sided *t* test. ****p < 0.0001; ***p < 0.001; **p < 0.01; *p < 0.05. Data are represented as mean values ± SEM

*EGFRvIII*-glioma cellular models are needed for pre-clinical studies. Cells from *EGFRvIII*-mouse GBMs were expanded ex vivo as gliomaspheres. We aimed to delineate the engrafting capacity of *EGFRvIII*-driven tumor cells as further evidence of their neoplastic nature by subcutaneous injection in the flanks of severe combined immunodeficient (NOD-SCID-γ) mice (Fig. 8a); subcutaneous rather intracranial injection was chosen as previous studies show the tumorigenic potential of mouse gliomas is equivalent for both methods [22] and tumor growth assessment was simplified. Tumors formed and were harvested within 20 days of injection in all mice (*n* = 6, Fig. 8c), and *EGFRvIII* was expressed in the vast majority of tumor cells as confirmed by immunostaining (Fig. 8b), demonstrating their transformed nature. Afatinib suppressed growth of these gliomaspheres with an IC50 of 0.1 μM (relative to equal volume vehicle treatment with DMSO; Additional file 1: Fig. S27a). Collectively, these data imply *EGFRvIII* is needed for initiation and maintenance of gliomagenesis in this model.

### Novel genes drive *EGFRvIII*-tumor cell proliferation and drug sensitization

We next decided to explore the putative tumor suppressive effects of genes not previously linked to gliomagenesis, but strongly implicated by our mutational analysis and *piggyBac* experiments—*Tead2*, *Nav3*, and *Spred1*. We performed CRISPR-Cas9 knockout experiments using ex vivo *EGFRvIII*-gliomaspheres derived from mouse GBMs. Lentiviral transduction enabled Cas9 expression from these tumor cells, and subsequent targeted sgRNA transduction led to the production of frequent on-target indels in coding exons of *Tead2*, *Nav3*, and *Spred1*. A non-targeting sgRNA was transduced as a wild-type control. Tumor cells with these alterations were assessed for gliomasphere growth at 4 weeks post-sgRNA transduction—loss of each of these genes led to significantly increased gliomasphere proliferation (*Tead2*-loss—6.44x, *Nav3*-loss—5.04x, *Spred1*-loss—3.58x, relative cell viability compared with non-targeting sgRNA control (1x); *p* < 0.0001, < 0.0001, and 0.036 respectively, adjusted one-way ANOVA test, 3D, CellTiter-Glo 3D cell viability assay, Fig. 8d, e, Additional file 1: Fig. S27b, c, Additional file 12: Table S11). These results confirm that loss of these genes heightens tumor cell proliferation and gliomasphere growth and highlight the use of *EGFRvIII*-mouse gliomaspheres as a platform for functional genetic validation studies.

To demonstrate the utility of our model for pre-clinical drug testing, we conducted a proof-of-principle experiment comparing the sensitivity of *EGFRvIII*-gliomaspheres, with and without CRISPR-induced mutations in *Nav3* and *Spred1*, to key small-molecule inhibitors. Although neither *Spred1* nor *Nav3* mutations affected tumor cell sensitivity to EGFR inhibition with afatinib (Additional file 1: Fig. S27a), loss of *Spred1* or *Nav3* increased sensitivity of *EGFRvIII*-tumor cells to MEK inhibitor treatment with trametinib (Fig. 8f, g). These preliminary results illustrate our model can be used for drug screening and the potential therapeutic relevance of *EGFR*-collaborative drivers.

## Discussion

Understanding how a cancer-initiating mutation influences downstream genomic evolution from human studies is challenging because of the absence of data from tumors before they become clinically overt, the large number of passenger mutations, co-occurring mutations caused by frank chromosomal anomalies and extensive tumor heterogeneity. Studies in mice offer a more uniform and defined genetic background and the opportunity to establish a temporal sequence of genetic changes, including in this case the introduction of a predisposing mutation which was activated during development.

We examined mouse tumor-genomic evolution through whole-exome sequencing and RNA-seq. Given the number of exonic mutations in each tumor was modest, genetic drivers could be discerned based on recurrent mutations as well as the impact of these on gene function. The most frequently mutated genes *Sub1*, *Trp53*, and *Tead2* had loss-of-function mutations, and recurrent focal deletions in other novel genes were detected; many of the mutated and deleted genes are also altered in human patients, supporting tumor suppressor roles. These findings are in keeping with elegant demonstrations of somatically acquired events driving mouse lung cancer evolution [92, 93]. Traditionally, mouse phenotypes have been solely attributed to the initiating events, but given somatically acquired events occur in GEMMs, these mutational landscapes should be considered in mouse glioma preclinical modeling and therapeutic efforts.

Given their genomic complexity, transcriptomes of these mouse tumors exhibited many changes from normal tissue. Recurrent amplification of *EGFRvIII* was observed, suggesting strong selection for increased expression, consistent with human gliomas where even extrachromosomal amplification of *EGFRvIII* has been reported [16, 17]. Although *EGFR* mutations are present in multiple GBM subsets (based on transcriptional profiles), our tumors most strongly enriched for the human mesenchymal GBM signature (a subtype more responsive to aggressive treatment) [44], likely because the specific cooperative drivers acquired in this model also occur in the human subset (including *Nf1*, *Pten*, and *Trp53*) [44].

By using conditional *piggyBac* insertional mutagenesis, there was a trend for increased GBMs, although the expected reduction in mouse survival [50] was not observed. This can be explained by the marked chromosomal instability (which *EGFR* can drive [59]) observed in the absence of transposition providing an adequate reservoir of additional mutations to facilitate oncogenesis. Supporting this is the obvious difference in ploidy observed in tumors from the two cohorts, similar to findings with *Sleeping Beauty* in osteosarcoma [48]. A difference in the spectrum of mutations was also apparent: in the absence of transposition, the most frequently mutated genes included known cancer genes, such as *Trp53*. These data imply *piggyBac* replaces the need for genomic instability for providing secondary molecular alterations to drive tumor progression.

Transposon studies complement human oncogenomic studies by pinpointing driver alterations hidden in large chromosomal aberrations of human cancer genomes and helping us prioritize key genes among the many alterations observed. Analysis of the CIS provided strong evidence for many known and novel genetic drivers collaborating with *EGFRvIII*. Multiple lines of evidence support this conclusion. First, the observation of integration sites in the same (CIS) genes in a significant fraction of the 96 tumors provides strong statistical evidence for selection of these events as putative driver mutations. Second, the position of these integrations with respect to the gene body and consequence on expression, consistently disrupting or activating gene expression, such as disruption of *Nf1* and *Spred1*. Third, RNA-seq data support the integration pattern because the transposon is designed to affect gene expression—transcripts were observed emanating from transposons splicing into *Rad51b*, as were transcripts from the gene splicing into the acceptor sites encoded by the transposon thereby disrupting gene expression such as *Cdkn2a*, *Nf1*, *Pten*, *Sox6*, *Sox5*, *Spred1*, *Qki*, and *Ust*. Fourth, the overlap of genes identified with mutations/focal deletions by exome sequencing and mutated by *piggyBac* cross-validates their biological selection—including *Cdkn2a*, *Esr1*, and *Myo10* (focal deletions) and *Nf1*, *Prex2*, and *Dgkb* (recurrent mutations). Finally, the correlation with human genetic data is compelling, not only for the known genes but also for genes like *SPRED1*, *TCF12*, and *SOX6*. The conserved role of these genes in both species validates the similarity and therefore relevance of the mouse model to human disease.

Relatively few activating insertions were detected with RNA-seq: this may reflect that glioma driver landscapes are dominated by tumor suppressors, but also be partly due to the biology of the transposon with gene disruption being a more likely event than activation. Although these mutations occurred in the context of mutant *EGFR* (implying genetic cooperation), this does not preclude these being drivers in other contexts without *EGFR*, as exemplified by *Pten* and *Nf1* also causing multiple glioma types with other drivers [94]. Future work will help provide mechanistic insight into the roles of the novel putative drivers identified here.

In this study, *EGFRvIII* initiated gliomas in mice after long latency. Previous studies reported *EGFRvIII* caused brain tumors with short latency, only in the presence of predisposing tumor suppressor loss, such as *Cdkn2a* and *Pten* [12, 21, 95], or with *Nras*^*G12D*^ and *Trp53* loss [96]. These differences may be due to the longer observation times here (allowing for accumulation of secondary mutations we identified by sequencing and *piggyBac*) and/or the *nes*-cre driver which also targets neurogenic niches such as the SVZ. The cell-of-origin (COO) of *EGFRvIII*-glioma was not the focus of this study but warrants further investigation. The COO of gliomas is unknown, yet evidence suggests that neural stem cells, oligodendrocyte precursors, and astrocytes can all act as the COO [97, 98].

A species differences between the mouse and human tumors is that most gliomas in this model were histologically low-grade, whereas in humans the majority with *EGFRvIII* mutations are histologically GBM. However, recent work on human samples demonstrates histologically low-grade appearing, *IDH1*-wildtype astrocytomas with *EGFR* amplification likely represent early GBMs with corresponding molecular features and poor prognosis [15, 99]; also, extrachromosomal *EGFR* driver mutations and amplifications are frequently detected in both human LGGs and GBMs [16]. Caution must thus be applied in histologically classifying these tumors in the absence of microvascular proliferation or necrosis. *EGFRvIII* is heterogeneously expressed in human GBMs, although the mutation has been detected throughout human GBMs suggesting it is an early event in some cases [18], and similarly is a clonal initiating event in these mouse tumors. The strength of the models here are molecular features recapitulating human *EGFR*-mutant gliomas, including the matched transcriptomic signatures and cooperative mutations.

It has been suggested *EGFRvIII* expression may induce senescence in the absence of tumor suppressor losses [21]. Although it is possible *EGFRvIII* led to transformation of rare cells with pre-existing tumor suppressor losses, multi-region transposon analysis revealed few clonal mutations making this possibility unlikely here. The genomic instability observed in the mouse tumors may be explained at least partially by oncogene-induced replicative stress, with the high frequency of *Cdkn2a* and *Trp53* mutations indicating strong selection for mechanisms to bypass oncogene-induced senescence in early gliomagenesis.

Few human genomic studies have been conducted for spinal tumors [26]. Although the frequency and nature of *EGFR* alterations (particularly extrachromosomal ones) in these tumors remains to be determined in larger studies, *EGFR* amplification and expression has been detected in a subset of human spinal tumors—leptomeningeal-disseminated pediatric spinal LGGs [24]. Clearly, not all tumor subsets can be recapitulated by one model, but this tumor subset shares a similar histology and unique location (leptomeningeal) as tumors from our mice. The mice have *EGFRvIII* as the driver, but these tumors could conceivably be generated by other mechanisms for increased EGFR signaling including alternative *EGFR* mutations, amplification, and/or overexpression. In patients, germline *NF1*-loss predisposes to spinal glioma [100] and a study of spinal gliomas detected frequent *CDKN2A* deletion and loss of heterozygosity at 10q23 (containing *PTEN*) [101]. Here, mice with conditional mutant *EGFR* and *Pten* loss exhibited accelerated spinal tumor development, confirming a key role of *Pten* in spinal gliomagenesis. This may have therapeutic implications—targeting Pten signaling, such as with PI3K inhibitors, may be warranted in spinal gliomas, although precise mechanisms of *Pten* loss driving spinal gliomagenesis must be explored [102].

Previous studies using the *Sleeping Beauty* transposon yielded common integration sites from gliomas [103–106], despite the incidence of tumors in some being low. Given *piggyBac* has different integration preferences and less local hopping (aiding genome-wide mutagenesis) compared with *Sleeping Beauty* [107–109], our work complements these studies. The model used here has the additional advantages of conditional rather than whole-body transposition (limiting tumor generation outside the CNS), being an autochthonous screen, and having a strong initiating mutation to drive complete penetrance of gliomas, increasing the power for detection of CIS driver genes.

Given the poor morbidity and mortality of glioma (particularly GBM) patients, additional therapies are needed. A key finding of this study is that many of the mutated glioma genes are druggable or predicted to be so. Several drugs suppressed growth even in wild-type *EGFR* human cell lines, supporting the idea that the drivers identified can act independently of *EGFR* too. Many of the glioma genes are putative tumor suppressors, which may be more challenging to target than oncogenes. However, tumor suppressors (and their downstream pathways) are increasingly regarded as potentially powerful therapeutic targets [110], particularly if a definite structure such as a pocket can be identified, as exemplified by molecules blocking the interaction of p53 with MDM2 thus increasing wild-type p53 [111].

Our novel models of gliomas will provide further opportunities for insights into their pathogenesis and therapeutic development. This is the first study to employ *piggyBac* mutagenesis in vivo in gliomas. The functional genomic datasets presented here will help decipher whole-genome sequencing studies of brain and spinal gliomas. Genome-wide *piggyBac* autochthonous screening in immunocompetent mice with high incidences of gliomas can feasibly be applied to explore resistance mechanisms to therapies. The finding of extensive cooperative mutations in mutant *EGFR* gliomas that can influence prognosis and drug treatment response highlights the importance of integrated genomic diagnosis for developing rational, personalized polytherapy strategies in patients to improve survival.

## Conclusions

Understanding the driver landscapes in the context of mutant *EGFR* is essential for advancing targeted glioma therapies. We show mutant *EGFR* is sufficient to initiate gliomagenesis in the brain and spinal cord. Through whole-exome sequencing, we defined the mutational landscape of these tumors in mice. Functional genomic landscapes of *EGFR*-mutant gliomas were elucidated by genome-wide *piggyBac* transposon mutagenesis and transcriptomics, identifying 281 known and novel cancer genes (tumor suppressors and oncogenes), with clinical relevance demonstrated by confirming corresponding human genetic alterations in patients. A genetic network susceptible to drug targeting was identified, providing potentially translatable therapeutic opportunities for gliomas.

## Methods

### Mice and genotyping

All animal experiments were in accordance with the Animal Scientific Procedures Act 1986 at the Wellcome Trust Sanger Institute (Hinxton, Cambridgeshire, UK). *EGFRvIII* mice were obtained from the NCI Mouse Repository, *nestin*-cre (*nes*-cre) mice from the Jackson Laboratory. *EGFRvIII* mice were crossed with tissue-specific *piggyBac* transposase (conditional transposase; TSPB) mice to yield *EGFRvIII*/+ TSPB/+ mice. The offspring were crossed with each other to yield homozygotes for both alleles (*EGFRvIII*/*EGFRvIII* TSPB/TSPB). Simultaneously, *nestin*-cre (*nes*-cre) mice were crossed with those carrying the ATP1S2 allele (containing 20 transposon copies) to yield *nes*-cre/+ ATP1S2/+ mice, which were then crossed with each other to give double homozygotes for these two alleles. To generate the main experimental cohort with both *EGFRvIII* expression and transposition, *EGFRvIII*/*EGFRvIII*; TSPB/TSPB mice were crossed with *nes*-cre/*nes*-cre; ATP1S2/ATP1S2 mice, giving mice heterozygous for these four alleles (*EGFRvIII*/+; TSPB/+; *nes*-cre/+; ATP1S2/+ = *EGFRvIII*-PB). Mice in the final experimental (*EGFRvIII*-PB) and control (*EGFRvIII*/+ *nes*-cre/+ ATP1-S2, or *EGFRvIII*/+; *nes*-cre/+ = *EGFRvIII*-only) cohorts were of mixed background. To generate *EGFRvIII*; *nes*-cre; *Pten*^+/−^ mice, *EGFRvIII* mice were crossed with *Pten*^*Loxp*^*/+* mice, and offspring carrying both alleles were crossed with *nes*-cre.

Genotyping of mice and PCR detection of transposition were completed as described previously [12, 27, 50, 54, 82]. The strains of the original mice are as follows: *EGFRvIII* mice are FVB, *nes*-cre mice are C57BL/6J; the ATP1S2 and TSPB mice are C57BL/6J albino.

### Necropsy and histopathological analysis

Mice were monitored daily in particular for neurological signs, including limb weakness, ataxia, hydrocephalus/macrocephaly, head tilt and/or circling, lethargy, and weight loss. Mice were sacrificed when the neurological signs were sufficient to impair basic functions. For downstream DNA and RNA extraction, tissue was immediately snap-frozen and/or placed in RNA-later. Tissues were fixed in 4% paraformaldehyde and then embedded in paraffin. Four-micrometer sections were stained with hematoxylin and eosin for morphological analysis. A consultant neuropathologist (SB), with expertise in neuro-oncological pathology of human and mouse tumors, and who was blinded to *EGFRvIII* and transposition genotype, reviewed all histological sections for pathological diagnosis. Neuropathological grading of gliomas was determined as follows: grade 1: tumors of low-to moderate cellularity, overall bland cytological appearance, bland nuclear morphology and only rare, or no mitotic figures; grade 2: tumors with moderate or high cellularity, occasional mitotic figures, and absence of microvascular proliferation and necrosis; grade 3: tumors with high cellularity, clear presence of mitotic figures, including brisk mitotic activity, hyperchromatic nuclei, but with no microvascular proliferations and no necrosis; and grade 4: highly cellular tumors, with densely packed nuclei, often a high nucleus to cytoplasm ratio, frequent mitotic figures, and with either microvascular (vascular endothelial) proliferations, or necrosis, or both. No characteristic histologic features of ependymomas or meningiomas were observed.

### Immunohistochemistry

Immunohistochemistry staining was performed using the Ventana Discovery XT instrument, using the Ventana DAB Map detection Kit (760-124). For pre-treatment, either Ventana CC1 (950-124), equivalent to EDTA buffer, or Protease 1 (equivalent to pronase, 760-2018), was used. Slides were hematoxylin counterstained. The antibodies used were as follows: Olig2 (1:100, Millipore ab9610), Sox2 (1:500, Abcam ab97959), Nestin (1:500, Abcam ab22035), Ki67 (1:100, Cell Signaling 12202S), GFAP (1:1000, Dako Z0334), PDGFRα (pre-diluted, Abcam ab15501), EGFR (Invitrogen 280005), and EGFRvIII (1:100, Sigma MABS1915).

### Glioma primary cultures

Mouse brain tumors were carefully dissected under the microscope. A small portion of the brain tumor was placed in cold saline on ice. This sample was then processed as soon as possible for primary culture establishment: it was incubated in Accutase (STEMCELL Technologies) for 15 min at 37 °C to dissociate the cells in sterile conditions. Cells were washed with PBS before being adding to culture medium and plated in a 6-well plate. The culture medium was composed of DMEM/F12 medium (50%), neurobasal medium (50%), hEGF (25 ng/ml), bFGF (25 ng/ml), N2 (1x), B2 (1x), BME (1x), and PSL (1x). The cultures were incubated at 37 °C and split every 2–3 days as required. To test for sensitivity to the EGFR inhibitor, afatinib (Selleckchem, S1011), the drug was added in varying concentrations (0–80 nM) to gliomaspheres in a 96-well plate on the day of plating (20,000 cells per well), with equal volume DMSO as a vehicle control. Spheres were counted and assessed for mean diameter after 10 days. The experiment was repeated in triplicate.

### Flank xenograft studies

5 × 10^5^*EGFRvIII*-GBM cells were subcutaneously injected into the flanks of NOD-SCID-γ mice (*n* = 6). Once tumors reached a maximum surface area of 1.2cm^2^, the mice were euthanized, and tumors were dissected and fixed in formalin for later embedding in paraffin.

### Fluorescence in situ hybridization (FISH)

For multiplex-fluorescence in situ hybridization (M-FISH), a chromosome-specific DNA library for each mouse chromosome was generated from 5000 copies of flow-sorted chromosomes, provided by the Flow Cytometry Core Facility of the Wellcome Trust Sanger Institute, using Genome-Plex Whole Genome Amplification (WGA2) kit (Sigma-Aldrich). A mouse 21-color painting probe was made following the pooling strategy (Jentsch et al. 2001). Five chromosome pools were labeled with ATTO 425-, ATTO 488-, CY3-, CY5-, and Texas Red-dUTPs (Jena Bioscience), respectively, using WGA 3 re-amplification kit (Sigma-Aldrich) as described before (Gribble et al. 2013). The labeled products were pooled and sonicated to achieve a size range of 200–1000 bp, optimal for use in chromosome painting. Sonicated DNA sample (enough for 10 hybridizations) was precipitated with ethanol together with mouse Cot-1 DNA (Invitrogen) and re-suspended hybridization buffer. Metaphase preparations were dropped onto pre-cleaned microscopic slides and followed by fixation in acetone and dehydration through an ethanol series. Metaphase spreads on slides were denatured by immersion in an alkaline denaturation solution and dehydration. The M-FISH probe was denatured before being applied onto the denatured slides. Hybridization was carried out in a 37 °C incubator for 2 nights. The post-hybridization washes included a 5-min stringent wash in 0.5 × SSC at 75 °C, followed by a 5-min rinse in 2 × SSC containing 0.05% Tween20 (VWR) and a 2-min rinse in 1 × PBS, both at room temperature.

Slides were mounted and images were visualized on a Zeiss AxioImager D1 fluorescent microscope equipped with narrow band-pass filters for DAPI, DEAC, FITC, CY3, TEXAS RED, and CY5 fluorescence and an ORCA-EA CCD camera (Hamamatsu). M-FISH digital images were captured using the SmartCapture software (Digital Scientific UK) and processed using the SmartType Karyotyper software (Digital Scientific UK). At least 10–20 metaphases per samples were fully karyotyped.

We quantified the cytogenetic anomalies found on FISH as follows: single translocations, copy number gains, or losses were counted as one anomaly for each chromosome; for polyploidy in all chromosomes, this was counted as one anomaly for each cell in which this was seen for a particular culture.

### Whole-exome sequencing

For whole-exome sequencing, extracted DNA was first quantified (using Accuclear UltraHS dsDNA Standards Assay reagent kit and BMG FLUOStar Omega fluorescence reader), followed by normalizing each sample to 4.17 ng/μl in 120 μl in preparation for library creation. DNA was sheared into fragments of 150 bp (on the Covaris LC220 and Agilent Bravo automated workstation) followed by library creation and amplification using unique indexed tags and adaptors (Agilent’s SureSelectXT Automated Library Prep & Capture Kits and MJ Tetrad). The amplified libraries were then purified (using Agencourt AMPure XP and Beckman Coulter Biomek NX96 automation) and eluted in nuclease-free water, followed by a second round of quantification. The libraries were then diluted down to an appropriate concentration for introduction into the exome-capture stage. Exome pulldown (hybridization) was performed using *Mouse-All-Exon* oligo-baits (Agilent) for 23 h at 65 °C. Uniquely indexed samples were baited and captured into pools. The pulldown was then purified and eluted using streptavidin-coated Dynal beads ready to be amplified (on the MJ Tetrad). The amplified product was further purified, followed by quantification using the Agilent Bioanalyzer and finally sequencing on the HiSeq Illumina 2500.

### Somatic variant calling and CNV analysis

Sequencing reads were mapped to the *Mus musculus* genome (GRCm38/mm10) using BWA-MEM (version 0.7.16a) [112] with default parameters. Duplicate reads were marked by biobambam2, and base quality scores were recalibrated with GATK (version 3.7) [113]. Sequencing coverage ranged from 50 to 80 x for each sample. Somatic variant calling of tumor and its matched normal BAM files were performed using Mutect2 (version 3.8) [114]. Mutations were annotated to a database of GRCm38.86 by SnpEff-4.3i [115]. Significantly mutated genes (SMGs) were identified by MuSiC (Version 0.4) [32] with default parameters; genes were called SMGs if mutated in two or more tumors, corrected likelihood ratio test *p* value < 0.01 and FDR < 0.2, and convolution test *p* value < 0.01. To detect somatic copy number alterations, the pileup files of tumor and its matched normal BAM files were generated by samtools mpileup (version1.5) [116], followed by copy number analysis using varScan2 (version 2.4.2) [117] with default parameters. Copy number variations were segmented using circular binary segmentation algorithm [118], which was implemented in DNAcopy (version 1.52). GISTIC2 (version 2.0.23) [35] with the following parameters: “qvt = 0.05, confidence level = 0.99, and maxseg = 20000” was performed to find focal CNVs using the *Mus musculus* (mm10) refSeq gene annotations.

### RNA-sequencing and bioinformatics analysis

RNA was purified from tumors and normal brain/spinal cord tissue (microdissected SVZ as brain tissue controls; all from age-matched control mice, *n* = 6) using the RNeasy microkit (Qiagen) according to the manufacturer’s instructions. RNA-seq libraries were constructed using the Illumina Tru-Seq Stranded RNA protocol with oligo dT pulldown and sequenced on Illumina HiSeq2500 by 75-bp paired-end sequencing. The RNA-seq data for samples were generated as 75 bp paired-end Illumina reads and aligned using STAR [119] to the human genome (GRCh37). The total number of reads that align to the exons of each gene as defined by Ensembl (version 75) [120] were obtained using STAR. The obtained gene counts were used obtain expression fold changes (FC) and false discovery rates (FDRs) for genes between any two conditions using DESeq2 [121]. The genes were considered differentially expressed if their − 2.0 > logFC > 2.0 and the Benjamini-Hochberg adjusted *p* value ≤ 0.01. These differentially expressed genes are given in Additional files 5 and 6: Tables S4 and S5. The gene set enrichment analysis (GSEA) against each of the MsigDB [122] datasets was performed using the GSEA tool [123].

To detect the presence of human *EGFRvIII* transcripts in RNA-seq data from mouse tumors (indicating that recombination of the conditional *EGFRvIII* allele has occurred), we introduced the human *EGFR* sequence with exons 2 to 7 removed into the mouse reference genome prior to RNA-seq alignment. The total number of reads aligned to the *EGFRvIII* gene was then counted as given above for all our RNA-seq samples. This process was applied both to brain and spinal tumors as well as to wild-type brain and spine control samples (that do not carry the *EGFRvIII* allele).

Transposon insertion sites from RNA-sequencing were obtained using IM-Fusion [80]. In any given sample, genes with at least one read traversing the transposon-gene junction or by a fragment (read pairs) spanning across the junction were identified. Based on the orientation of the inserted transposon and the feature (splice donor, or splice acceptor) of the transposon inserted, the gene transcript was either declared as activated or truncated. As controls, we analyzed 10 EGFRvIII; *nes*-cre; ATP1S2 tumors (lacking TSPB)—there were no read counts supporting fusion transcripts in these tumors, implying detection of fusion transcripts occurs specifically in the presence of transposition only as expected.

### Splinkerette PCR and Illumina sequencing

Tradis library preparation was performed as described before [63]. Briefly, DNA extracted from tumor tissue was quantified using the Qubit and sheared on the Covaris AFA sonicator to a mean fragment size of 250 bp (with re-shearing to be done if the fragment size were considerably larger). DNA samples were subjected to custom Splinkerette adaptor ligation. A PCR for amplification of the adaptor-ligated library to enrich for transposon-containing amplicons was performed using KapaHiFi HotStart and a separate primer for each DNA end (3′ and 5′), with 18 PCR cycles. A further 12-cycle transposon-PCR was performed using a separate primer for each library (one for 3′ and one for 5′) and an index primer for each individual sample (allowing for multiplexing of the samples for sequencing). To avoid individual samples being heavily overrepresented in the sequencing pool, the indexed samples in the libraries were quantified by quantitative PCR and then combined into an equimolar pool. Each library pool (one for each transposon end) was sequenced on the Illumina MiSeq platform in a separate sequencing run yielding, on average, 10 million 75 bp paired-end reads. The libraries were multiplexed for up to 55 samples in each pool in this study, requiring 4 MiSeq runs in total, in order to give high coverage sequencing.

### Insertion mapping

We used the Gaussian kernel convolution (GKC) approach of de Ridder et al. [124] for identifying *piggyBac* (PB) common insertion sites (CIS), as described previously [54, 63]. CIS are genomic regions of several tens of kilobases in length where transposons insert significantly more frequently than by chance considering the background rate of insertions and number of TTAA canonical insertion motifs. Briefly, the sequencing reads were filtered for Splinkerette primer sequences contained within the PB ITRs. Transposon insertion sites (IS) were established by mapping the sequencing reads to the mouse genome (assembly version GRCm38) using the SMALT aligner (http://smalt.sourceforge.net). For each tumor sample, sequencing reads mapping to the same location in the genome counted as a single IS. The top 300 IS, by read count, of each sample were pooled in a non-redundant set and subjected to a GKC analysis with “window sizes” (kernel widths), ranging from 10 to 100 kb in 10 kb steps. Similar numbers of CIS were found for each window size, and most CIS were detected across multiple windows. Significant CIS were taken to be those with a Bonferroni-corrected *p* value < 0.001 for multiple window sizes. Significant CIS were associated with genes as annotated in ENSEMBL release 90 [125]. Mouse genes labeled as “predicted” in the ENSEMBL annotation were not considered in the analyses. Cancer genes were obtained from COSMIC v82 [126].

### Pathway and network analysis

Pathway analysis was performed using DAVID [127] (with KEGG, Biocarta, and GO-term datasets) for transposon and RNA-seq data; for the latter, GSEA was performed as above. Further analysis of transposon genes was performed using the *Panther* tool, focusing on the pathway gene sets. For network analyses to determine functional connectivity between CIS genes, we used the STRING tool to visualize known and predicted interactions between proteins [65].

### Glioma comparative genomics analysis

Data on somatic variant and copy number variant calls, RNA-seq expression *z*-scores, and methylation scores were obtained through Cbioportal from TCGA human low-grade glioma and glioblastoma datasets on 10 December 2017 [128]. Patient survival data from TCGA and REMBRANDT GBM datasets were analyzed through the Betastasis software (www.betastasis.com). These were brain tumors; there are currently no such large genomic datasets from human spinal gliomas for comparative genomics analysis. Mutual exclusivity and co-occurrence between genetic alterations in these datasets were determined by the Fisher’s exact test, with a Bonferroni adjusted *p* value < 0.05 taken as statistically significant.

For comparative transcriptomic analysis for *Hox* genes, RNA-seq log_2_ fold changes and *p* values were taken for the three large human patient datasets described comparing GBM with normal brain tissue, from publicly available Oncomine software (www.oncomine.org). To capture all levels of differential expression, *p* value significance was set at 0.05 and fold change/gene rank were set to “all.”

### Clonal heterogeneity analysis

To analyze intratumoral clonal heterogeneity, we sampled three independent sites from two established *EGFRvIII*-PB gliomas. DNA from the samples was extracted for Splinkerette PCR and Illumina sequencing, followed by insertion mapping as described above. Given that lineage relationships between tumor samples can be inferred from patterns of shared mutations, we identified the matching insertions in CIS genes (with supporting read count of 2 or more for a particular insertion) between different regions of the same tumor, as well as the unique insertions in CIS genes for a given tumor region.

### Lentiviral production

For lentiviral production of sgRNA constructs, 2.2 × 10^4^ 293FT cells were plated on each well of 96-well plate and 24 h later were transfected with 25 ng of the lentiviral vector, 75 ng of Virapower Lentiviral Packaging Mix (Invitrogen) per well with the Lipofectamine LTX & PLUS reagent (Invitrogen). The viral media was collected and pooled at 72 h post-transfection, centrifuged at 1000*g* for 5 min prior to filtration.

### CRISPR-cas9 knockout assays

Neurospheres from *EGFRvIII*-mouse gliomas were dissociated into single cells with Accutase and transduced with lentiCas9-Blast (Addgene #52962) in the presence of 2 μg/ml polybrene. Media was changed 24 h after transduction and selection performed with 2.5 μg/ml blasticidin. Cas9 expression was confirmed by qPCR. Single-guide RNAs (sgRNAs) against target genes of interest were selected using from the Brie library [129] and were ligated into the sgRNA expressing plasmid (Addgene #67975). Cas9-GBM cells were infected with lenti-sgRNA lentivirus as described above and selected with puromycin for 1 week before seeding in the proliferation/drug viability assays. Three independent experiments were performed in triplicate for each condition. TIDE was used to assess the efficiency of gene editing with PCR amplicons flanking the cutting sites. Additional file 11: Table S10 shows the sequences for all sgRNAs used.

Cell viability was assessed using the CellTiter-Glo-3D cell viability assay (Promega). Briefly, *EGFRvIII*-GBM cells were plated in a 24-well opaque-walled plate, with 2500 cells per well. CellTiter-Glo-3D reagent was added in equal volume to each well, and the plate was mixed by shaking for 5 min then incubated at room temperature for 25 min. Luminescence was quantified using a luminescent plate reader.

To measure the effect of drugs on cell viability, single-cell split *EGFRvIII*-GBM cells were plated in a 96-well plate (5000 cells per well). Cells were left untreated, treated with dimethyl sulfoxide (DMSO), trametinib, or afatinib at concentrations of 0.001, 0.01, 0.1, 1, and 10 μM. After 4 days of treatment, cell viability was assessed using CellTiter-Glo-3D. Three independent experiments were performed in triplicate for each condition.

### Chemogenomic analysis

We constructed a core set of glioma genes by combining all CIS genes (*EGFRvIII*-PB cohort; 281 genes) and all recurrent significantly mutated genes (*EGFRvIII*-only cohort; 85 genes), in addition to 9 additional genes whose proteins are directly activated by loss of genes in the set (AKT1, AKT2, ERK1, ERK2, MEK1, MEK2, HRAS, NRAS, KRAS). This yielded set of 375 proteins. To determine if the corresponding genes are genetically altered in LGGs and GBMs in patients, the TCGA dataset was checked for heterozygous/homozygous deletions, amplifications, and mutations using Cbioportal; all genes with at least one tumor containing one of these alterations were included as genes also altered in patients. To annotate the glioma protein set with druggability and pharmacological data, we used the canSAR software (Cancer Protein Annotation Tool, CPAT, https://cansarblack.icr.ac.uk) [130]. Druggable proteins in the set were classified as (1) targets of clinically approved drugs (approved for indications other than glioma); (2) targets of drugs in clinical investigations; (3) targets of drugs at discovery or preclinical stages active against the proteins at concentrations of less than or equal to 100 nM; or (4) proteins predicted to be druggable using established structural druggability prediction methods [84, 130–132], which are potential future drug targets.

Proteins with compounds available were assessed for human glioma cell line sensitivity using the Genomics of Drug Sensitivity in Cancer (GDSC, www.cancerrxgene.org) database. IC_50_*Z*-scores represent the relative sensitivity of a cell line to a given drug relative to all other cancer cell lines tested, with a value of − 2.0 taken to be statistically significant for sensitivity and values between − 0.5 and − 2.0 taken to represent partial or weak sensitivity [91].

### Statistical analysis

Software calculations were performed using Microsoft Excel, GraphPad Prism version 7 or R version 3.2.0 (The R Project for Statistical Computing, http://www.r-project.org/). The *p* values, specific test, and data representation for each analysis is described in the main text or figure legends. Data were verified to meet the assumptions of the statistical tests used.

### Cell line authentication

Cell lines used in this study were directly derived from the mice generated, so formal authentication was not applicable.

## Supplementary information

**Additional file 1:** All supplementary figures with legends.

**Additional file 2: Table S1.** Mouse Phenotypes.

**Additional file 3: Table S2.** Significantly Mutated Genes.

**Additional file 4: Table S3.** Significant deletions.

**Additional file 5: Table S4.** RNA-seq in brain tumors.

**Additional file 6: Table S5.** RNA-seq in spinal tumors.

**Additional file 7: Table S6.** CIS genes.

**Additional file 8: Table S7.** Fusion transcripts.

**Additional file 9: Table S8.** Druggable genes.

**Additional file 10: Table S9.** Chemogenomic analysis.

**Additional file 11: Table S10.** GDSC glioma drug sensitivities.

**Additional file 12: Table S11.** sgRNA sequences.

## Data Availability

All the sequencing data generated in this study are available from the European Nucleotide Archive (ENA), https://www.ebi.ac.uk/ena, accession code ERP024282 [133]. Third party datasets analyzed include the Verhaak and TCGA datasets [36, 37, 44]. Cell lines generated herein can be provided to researchers on written request to the corresponding author.
