# Correction to: PiggyBac mutagenesis and exome sequencing identify genetic driver landscapes and potential therapeutic targets of EGFR-mutant gliomas

**DOI:** 10.1186/s13059-020-02127-8

**Published:** 2020-08-17

**Authors:** Imran Noorani, Jorge de la Rosa, Yoon Ha Choi, Alexander Strong, Hannes Ponstingl, M. S. Vijayabaskar, Jusung Lee, Eunmin Lee, Angela Richard-Londt, Mathias Friedrich, Federica Furlanetto, Rocio Fuente, Ruby Banerjee, Fengtang Yang, Frances Law, Colin Watts, Roland Rad, George Vassiliou, Jong Kyoung Kim, Thomas Santarius, Sebastian Brandner, Allan Bradley

**Affiliations:** 1 The Wellcome Trust Sanger Institute, Wellcome Trust Genome Campus, Hinxton, Cambridgeshire, CB10 1SA UK; 2 Department of Neurosurgery, Addenbrookes Hospital, University of Cambridge, Hills Road, Cambridge, CB2 0QQ UK; 3 Department of New Biology, DGIST, 333, Techno Jungang Daero, Hyeonpung-Myeon, Dalseong-Gun, Daegu, 42988 South Korea; 4 Division of Neuropathology and Department of Neurodegenerative Disease, UCL Institute of Neurology, Queen Square, Mailbox 126, London, WC1N 3BG UK; 5 Department of Internal Medicine II, Klinikum rechts der Isar, Technische Universität München, Ismaninger Strasse 22, 81675 Munich, Germany; 6 Birmingham Brain Cancer Program, Institute of Cancer and Genomic Sciences, College of Medical and Dental Sciences, University of Birmingham, Edgbaston, Birmingham, B15 2TT UK

**Correction to: Genome Biol 21, 181 (2020)**

**https://doi.org/10.1186/s13059-020-02092-2**

Following publication of the original paper [1], the authors identified errors the paper.

Under the heading *Whole-Exome Sequencing Reveals The Mutational Landscape* in the results section, the following text has been updated. The updated text is displayed in **bold typeface**.

In contrast to the relatively small number of recurrent mutations, *EGFR*-mutant tumors had complex genomes by DNA copy number analysis (Fig. 2b). Significant focal amplifications and deletions, identified by GISTIC2 ^35^, were evident in regions with known cancer genes, for example significant focal *Cdkn2a* deletions (GISTIC q-value = 1.39 × 10^− 5^) were evident and *EGFRvIII* (in *Col1a1* locus, GISTIC q-value = 0.017) was recurrently amplified. Significantly recurrent focal deletions were present in a novel putative glioma driver *Adgrl2* (GISTIC q-value = 2.19 × 10^− 6^, Additional File 4: Table S3). **Although focal deletions in** ***Nlrp1b*** **were present, recent evidence suggests these represent a strain-specific germline variant rather than being oncogenic** [2]. Several of the most significantly mutated genes were also in regions with frequent deletions, including *Trp53*, *Tead2* and *Uimc1*, supporting putative tumor suppressive roles (Fig. 3i).

The caption of Fig. 3 has also been updated. The correct caption is supplied below. The updated text is displayed in **bold typeface**.

Figure 3. Conditional *PiggyBac* transposon mutagenesis substitutes for genomic instability in *EGFRvIII*-mutant gliomas. A. Mouse constructs for *PiggyBac* transposition. The ATP1-S2 transposon line, with 20 copies per cell. Conditional *PiggyBac* transposase targeted to *Rosa26* (tissue-specific *PiggyBac* transposase, TSPB), SA = splice acceptor; SD = splice donor; CAG = CAG promoter; SB = Sleeping Beauty; PB = *PiggyBac* inverted repeats; iPBase = insect version of the *PiggyBac* transposase. The transposon can activate gene transcription if it inserts in the same orientation as the gene, usually in a 5′ position. Gene inactivation can occur if the transposon inserts in the body of the gene as a consequence of gene trapping which can occur in either orientation because of the presence of two splice acceptors and bidirectional poly(A) (pA) sites. B. Outline of the experimental design: quadruple transgenic mice conditionally activate *EGFRvIII* expression and *PiggyBac* transposition in the central nervous system. Resultant tumors are examined molecularly by whole-exome sequencing and mapping of transposon insertions. C. Histology of *EGFRvIII*-PB tumors; although not statistically significant, a higher proportion of grade IV brain tumors are observed compared with tumors lacking transposition. D. Immunostaining profile of a typical grade III brain glioma from an *EGFRvIII*-PB mouse, showing strong expression of neural stem and transit-amplifying cell markers. Scale bar corresponds to 2.8 mm for top panel, and 200 μm for all other panels. E. Representative karyotype of *EGFRvIII*-only and *EGFRvIII-*PB brain tumors, showing polyploidy in the non-PB tumor. F. Chromosomal aberrations in *EGFRvIII*-only and *EGFRvIII*-PB tumors (*n* = 3 and *n* = 5 tumors respectively; mean chromosomal aberrations 19 vs 6.4, *p* = 0.013, unpaired two-tailed t-test; plots show mean +/− standard deviation). G. Copy number profile of *EGFRvIII*-PB tumors (*n* = 20) with focal amplifications and deletions in key genes highlighted. H. Mutational profile of 20 *EGFRvIII*-PB brain and spinal tumors from whole-exome sequencing. I. Key genes identified **in gliomas**, either as significantly mutated from MuSiC or copy number altered from GISTIC2, across all mouse brain and spinal tumors in both cohorts **(note** ***Nlrp1b*** **deletions are however a germline variant** [2]**)**; each column represent one tumor.

In addition, the authors identified an error in the author name of Yoon Ha Choi.

The incorrect author name is: Yoonha Choi

The correct author name is: Yoon Ha Choi

## References

[1] Noorani I, de la Rosa J, Choi Y (2020). *PiggyBac* mutagenesis and exome sequencing identify genetic driver landscapes and potential therapeutic targets of *EGFR*-mutant gliomas. Genome Biol.

[2] Mueller S, et al. Linkage of genetic drivers and strain-specific germline variants confound mouse cancer genome analyses. Nat Commun. 2020; In press. 10.1038/s41467-020-18095-3 PMC7479137 32901003

