# Supplementary material for: PiggyBac mutagenesis and exome sequencing identify genetic driver landscapes and potential therapeutic targets of EGFR-mutant gliomas
Source: Genome Biol. 2020 Jul 30;21:181. doi: 10.1186/s13059-020-02092-2 (PMC7392733; doi:10.1186/s13059-020-02092-2)
Supplement: Supplementary file 1 — Additional file 1: All supplementary figures with legends. [file 13059_2020_2092_MOESM1_ESM.pdf]

EGFRvIII-induced brain microneoplasias

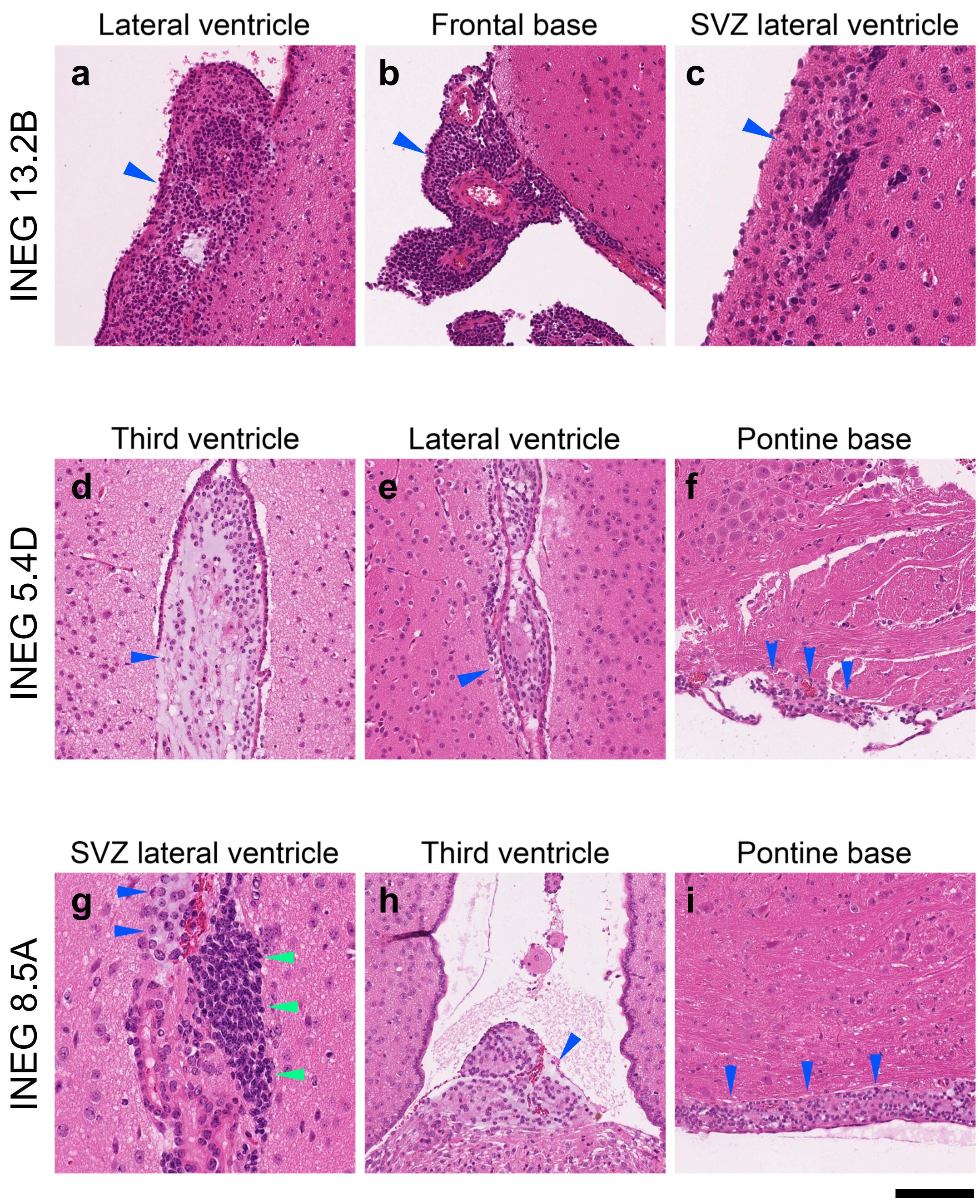

Figure S1

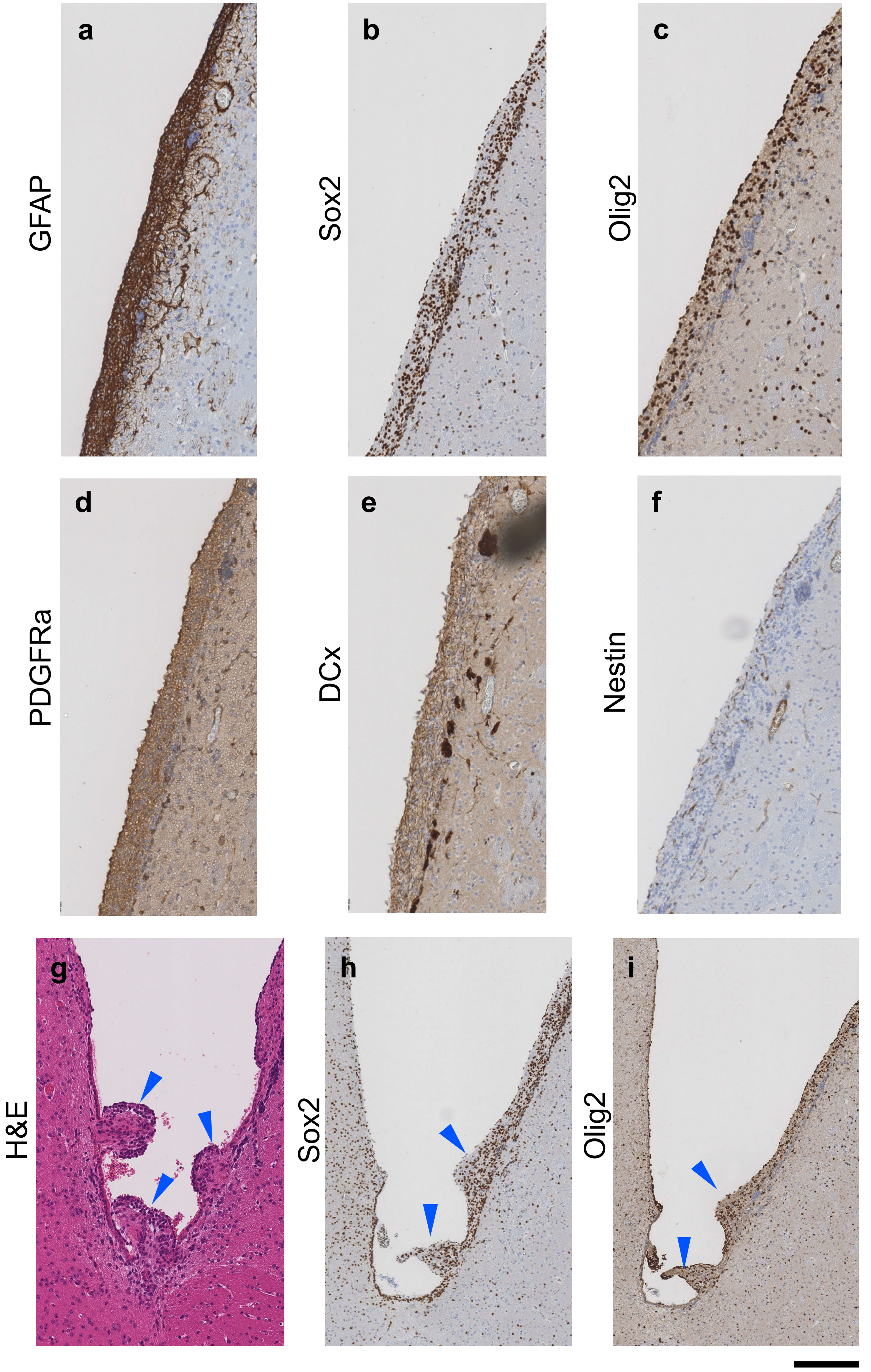

Figure S2

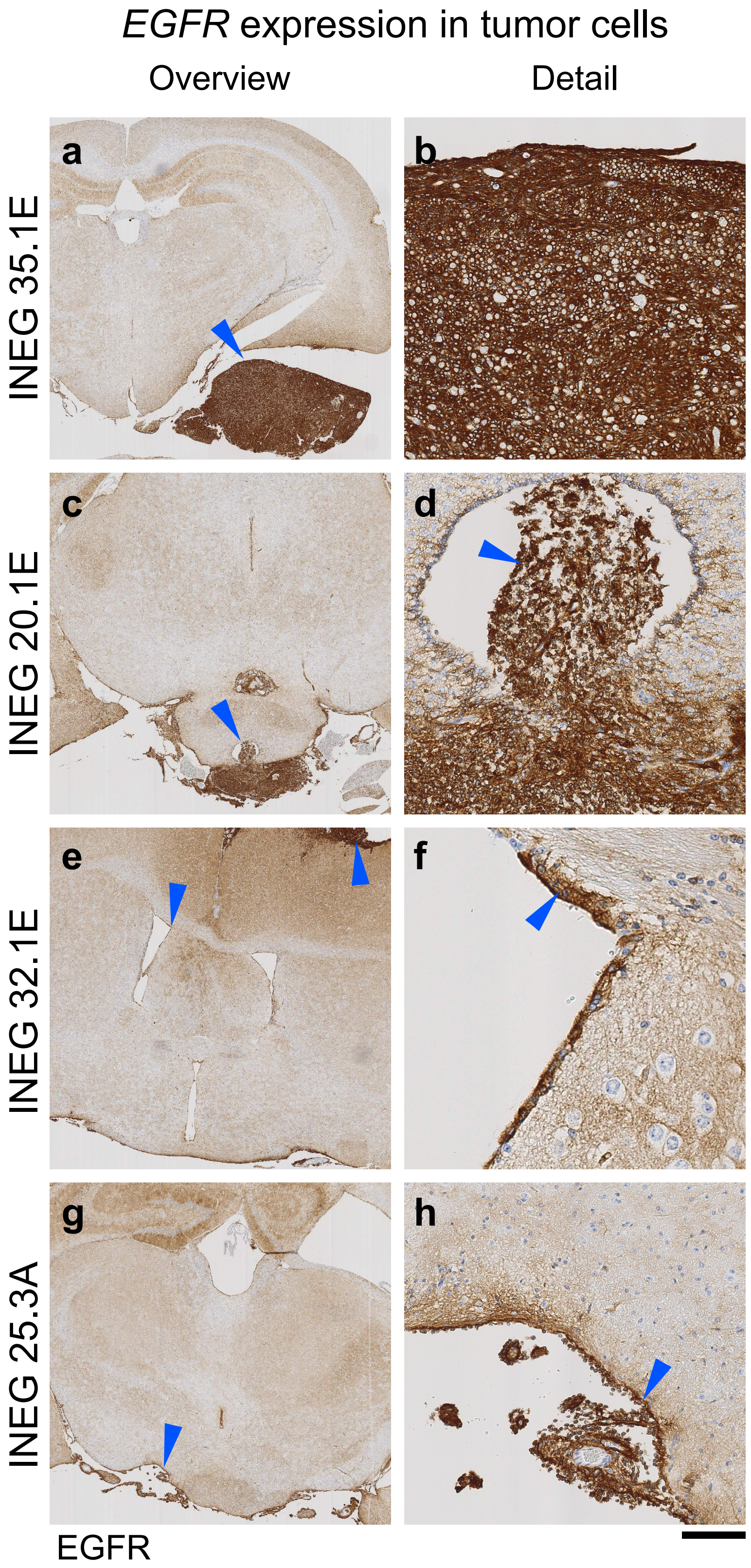
EGFR expression in tumor cells
Overview
Detail
INEG 35.1E
INEG 20.1E
INEG 32.1E
INEG 25.3A
a
b
c
d
e
f
g
h
EGFR

Figure S3

EGFRvIII recombination in tumor cells

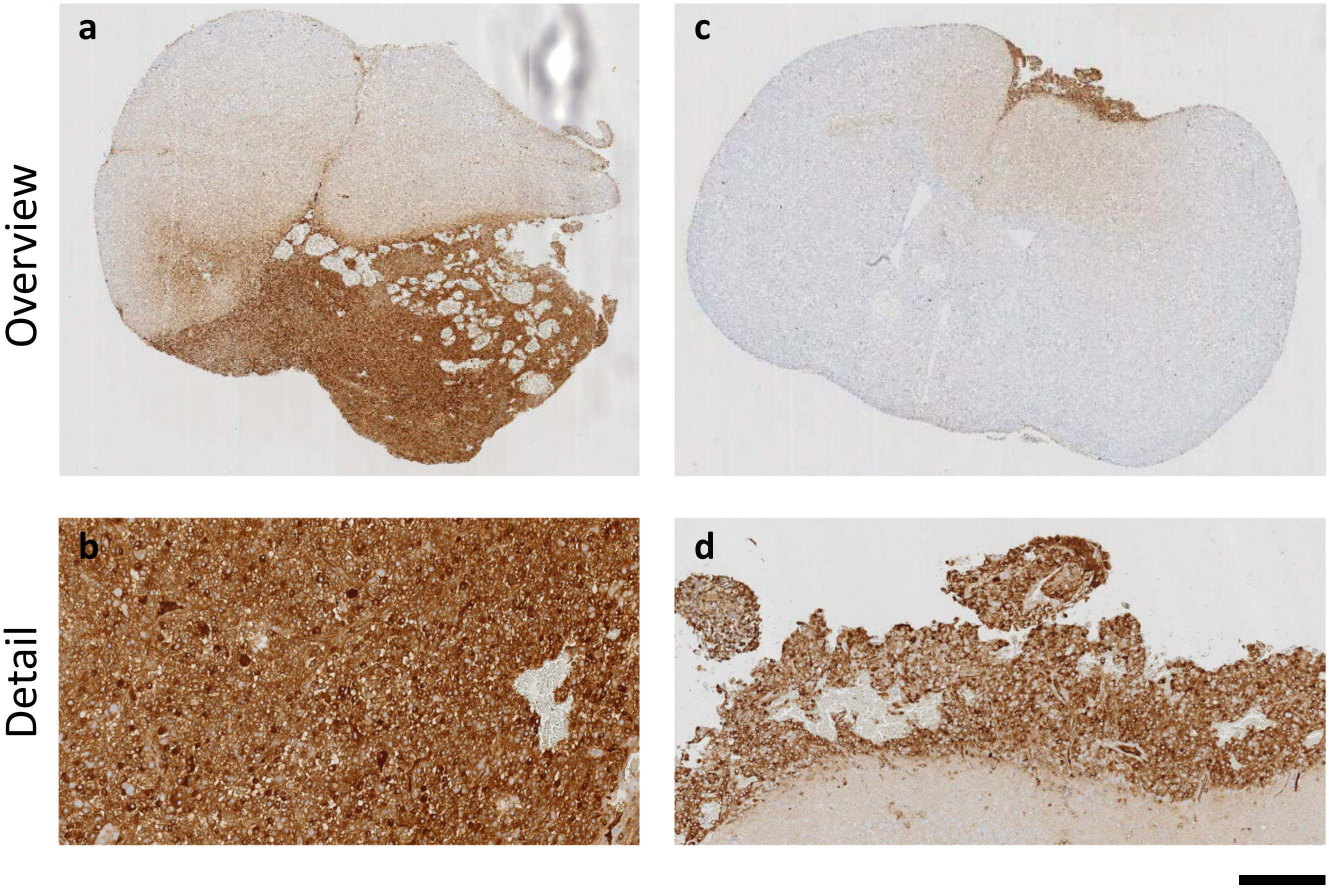

Figure S4

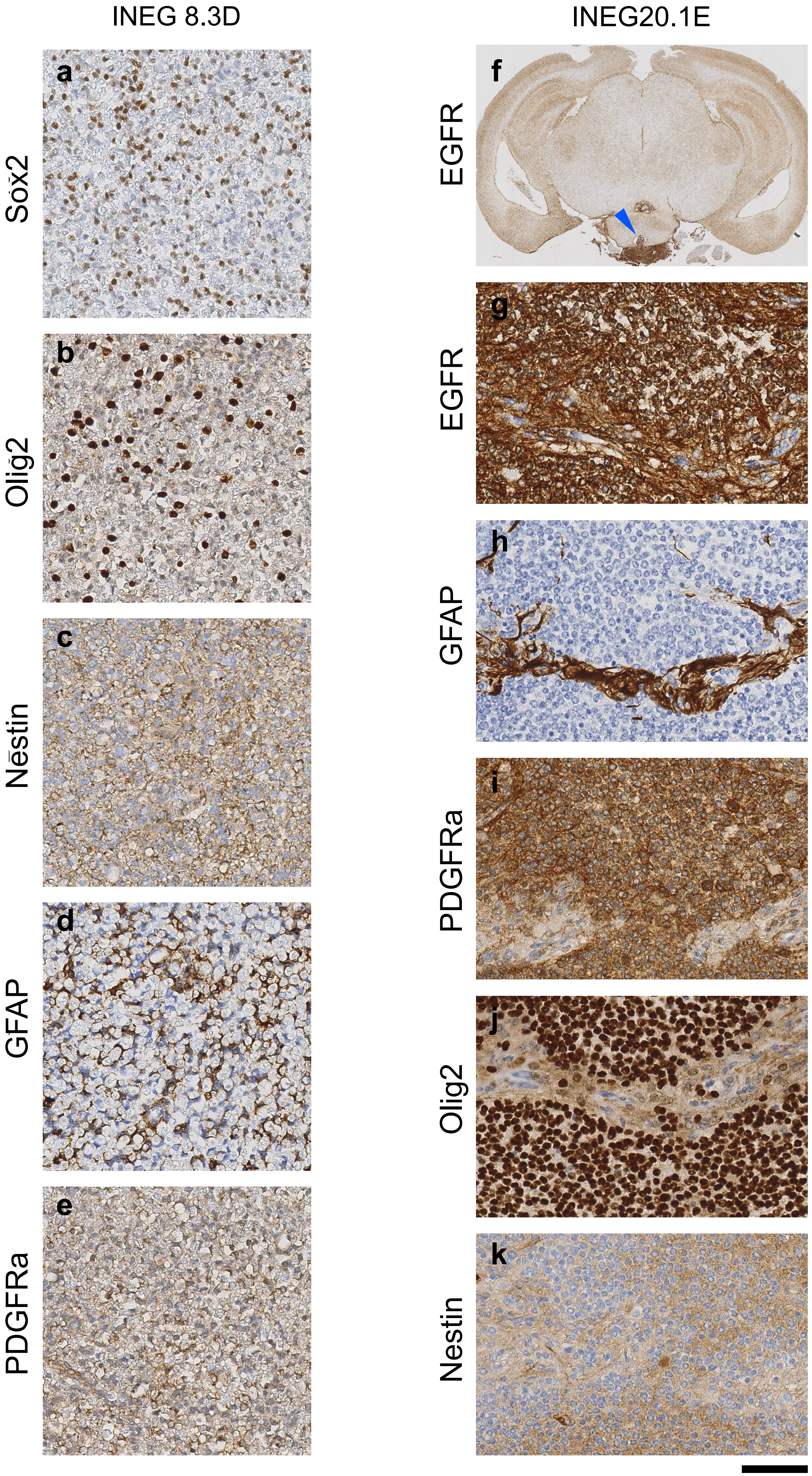

INEG 8.3D
INEG20.1E
a
Sox2
b
Olig2
c
Nestin
d
GFAP
e
PDGFRa
f
EGFR
g
EGFR
h
GFAP
i
PDGFRa
j
Olig2
k
Nestin

Figure S5

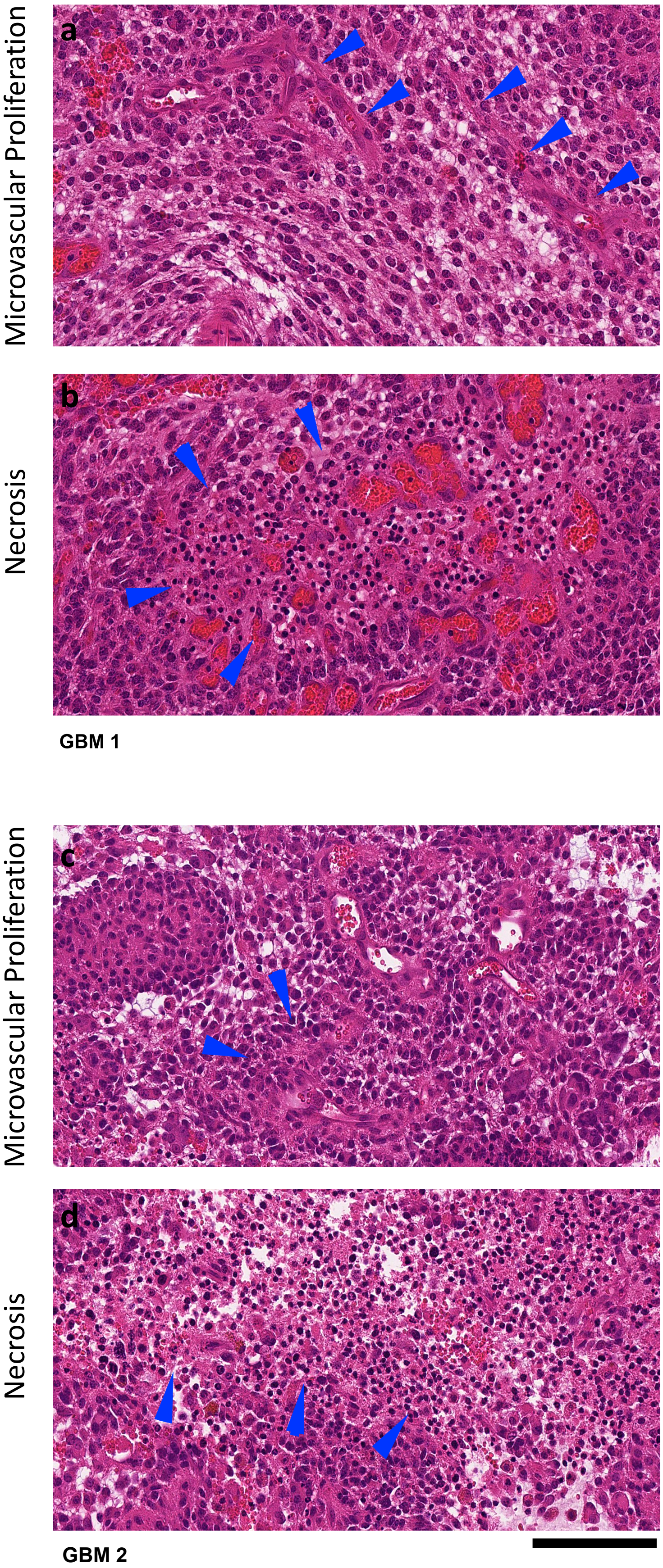
Microvascular Proliferation
a
Necrosis
b
GBM 1
Microvascular Proliferation
c
Necrosis
d
GBM 2

Figure S6

Ki67 Proliferation index of microneoplasias in a brain of an *EGFRvIII* ; Nes-cre mouse

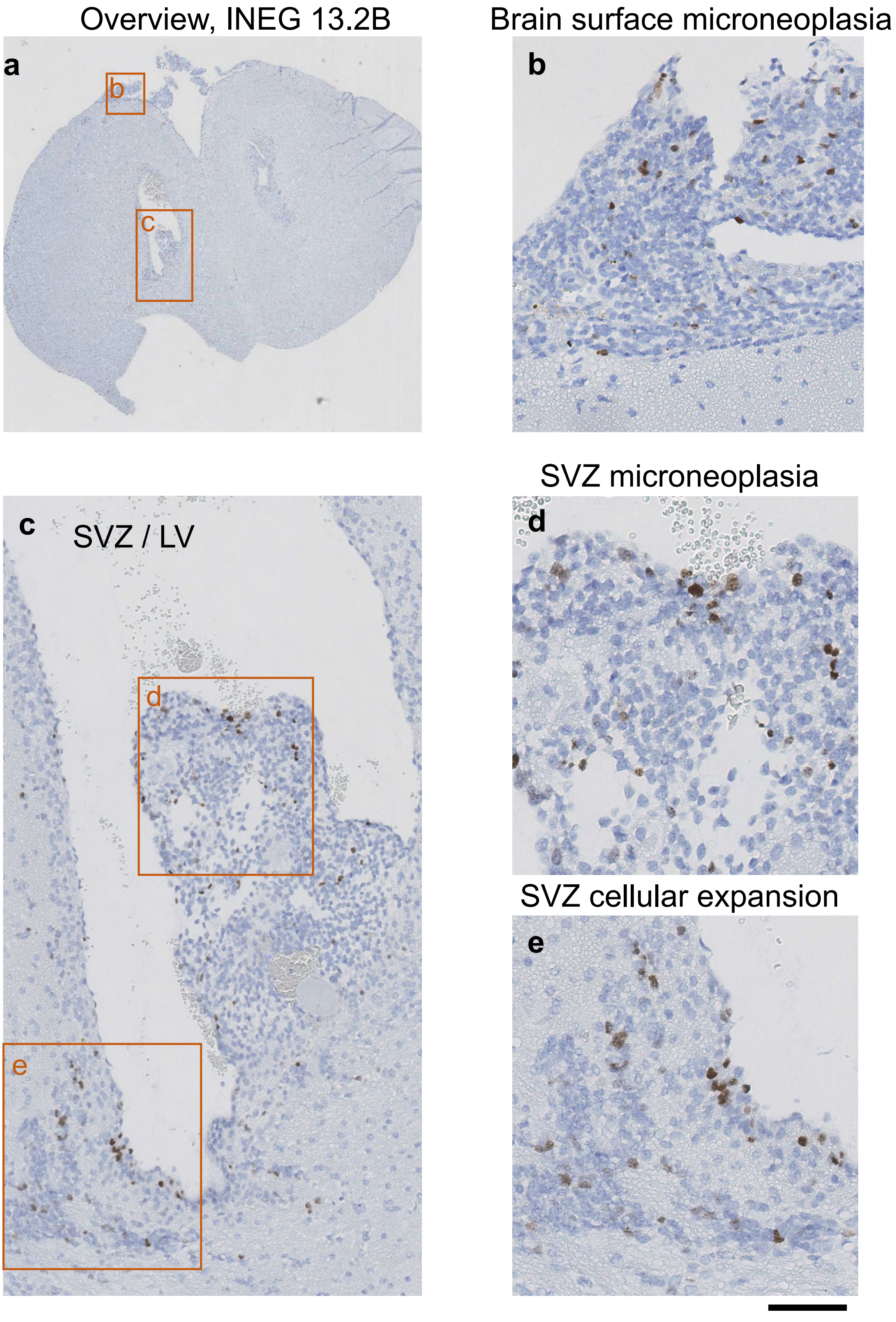

Figure S7

Glioma induction on spinal surface

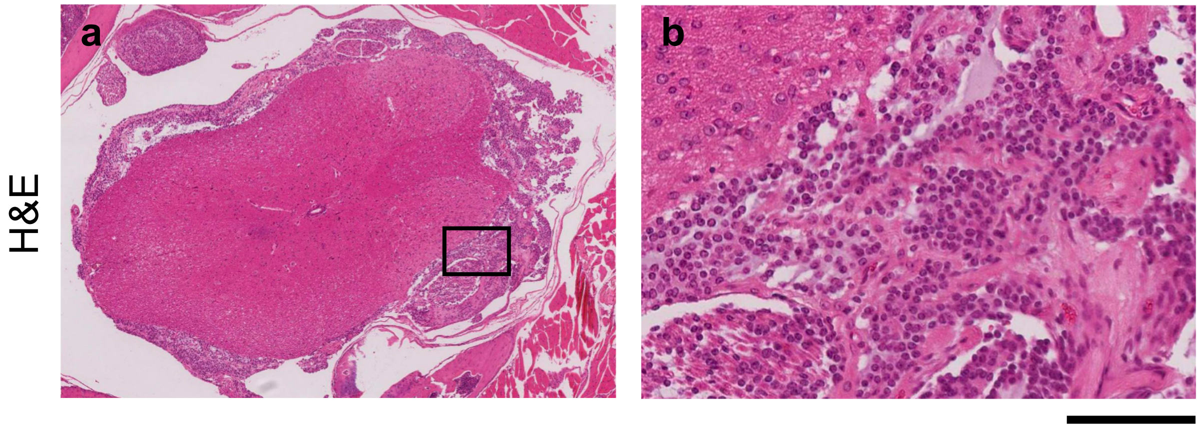

Figure S8

a

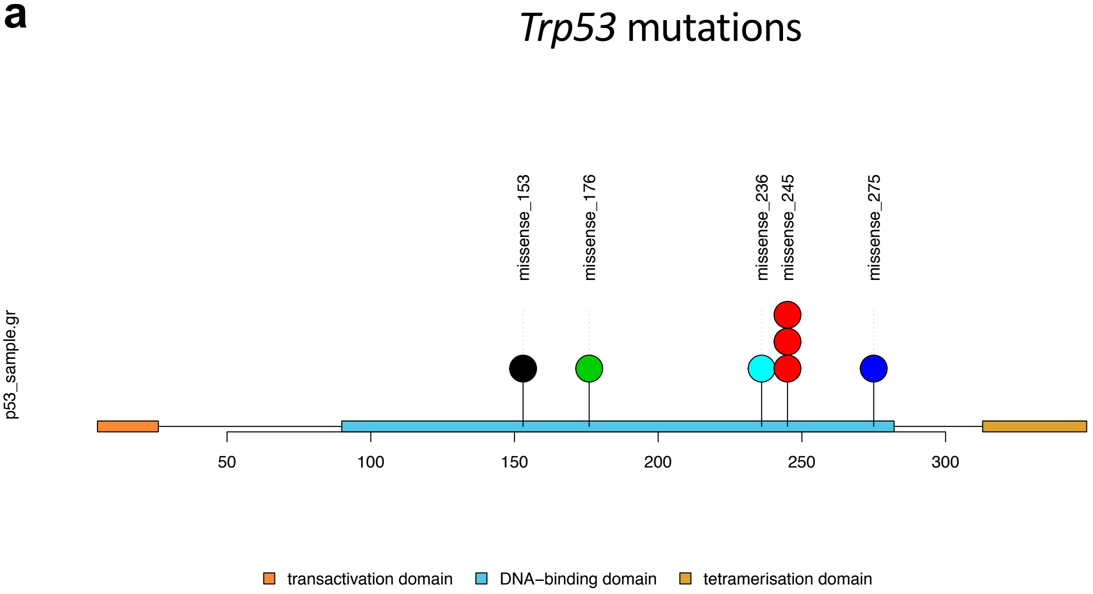

Trp53 mutations
p53_sample.gr
missense_153
missense_176
missense_236
missense_245
missense_275
50
100
150
200
250
300
transactivation domain
DNA–binding domain
tetramerisation domain

b

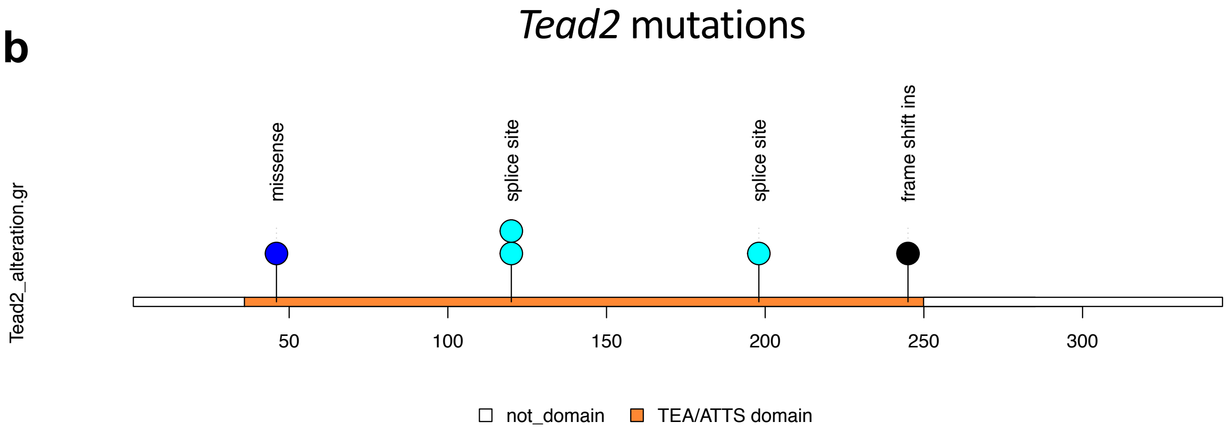

Tead2 mutations
Tead2_alteration.gr
missense
splice site
splice site
frame shift ins
50
100
150
200
250
300
not_domain
TEA/ATTS domain

Figure S9

a

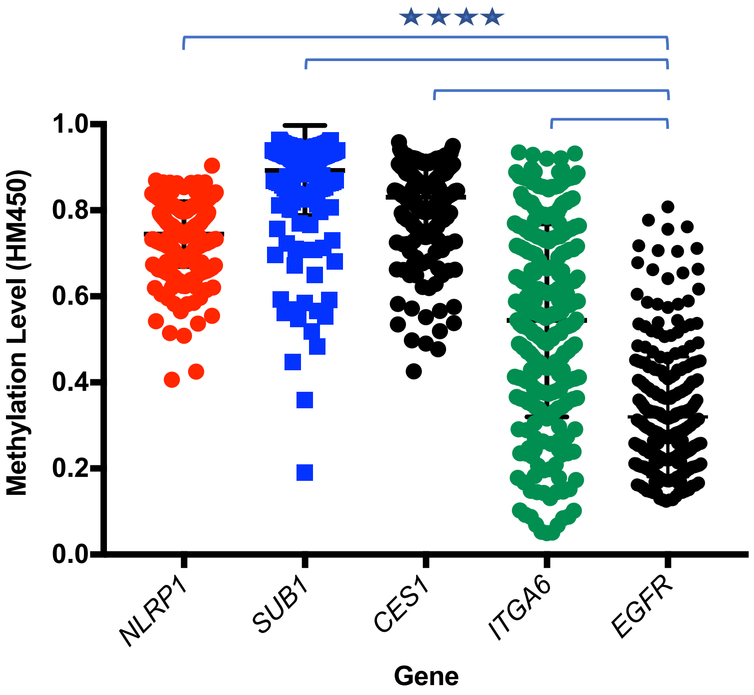

b

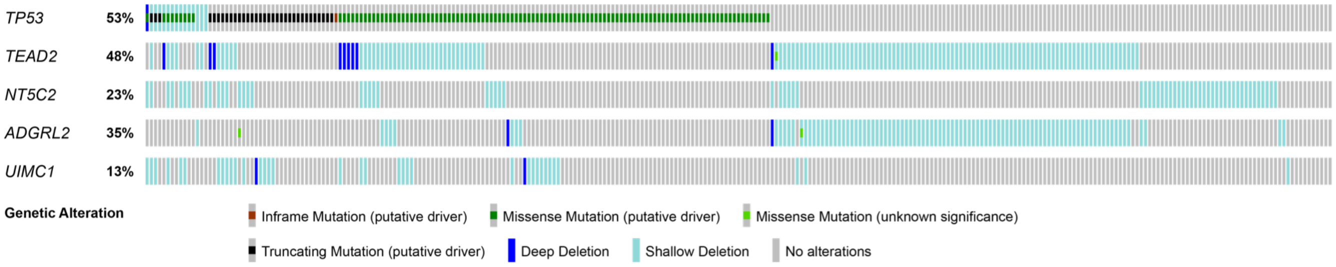

Figure S10

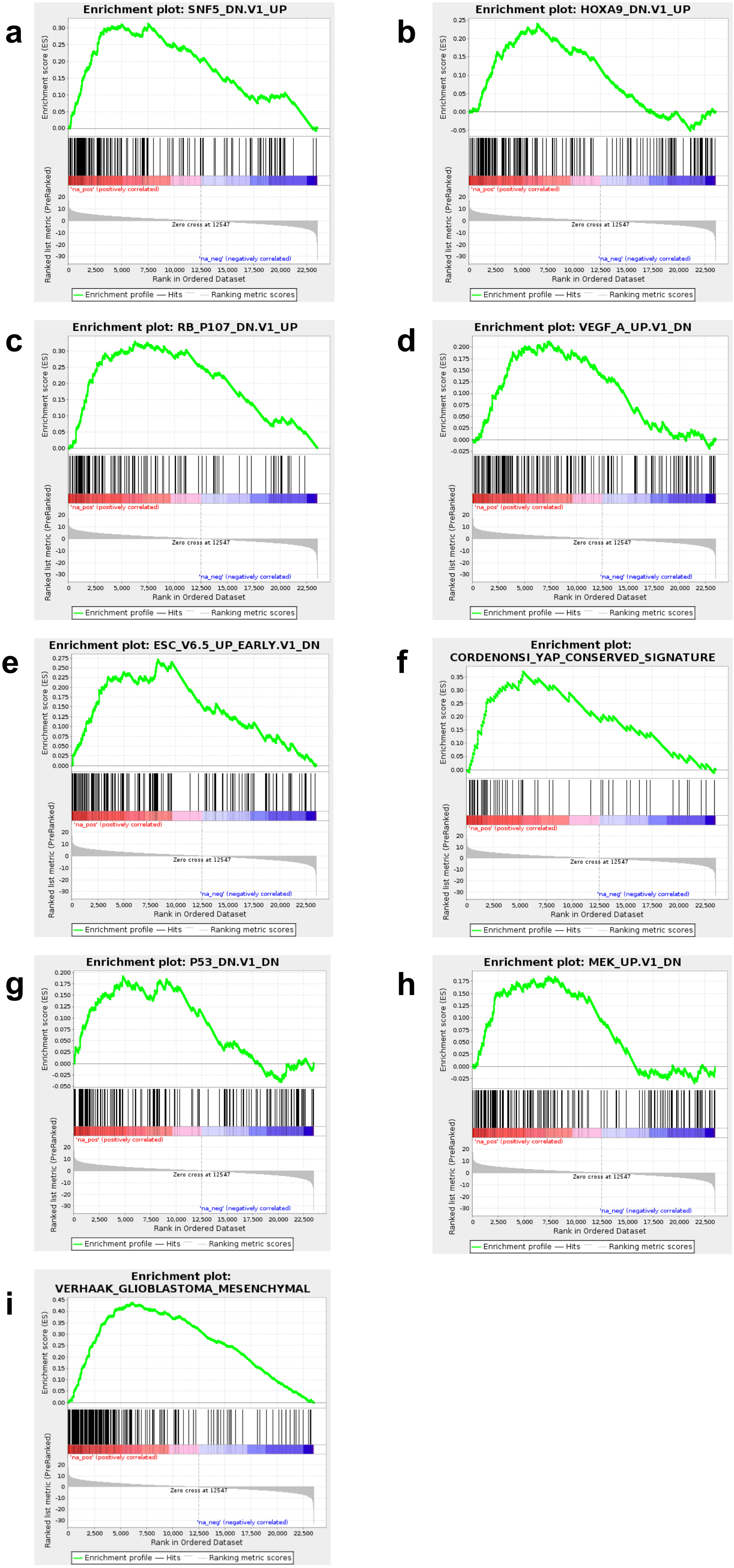
a
Enrichment plot: SNF5_DN.V1_UP
Enrichment score (ES)
'na_pos' (positively correlated)
Zero cross at 12547
'na_neg' (negatively correlated)
Ranked list metric (PreRanked)
Rank in Ordered Dataset
Enrichment profile — Hits — Ranking metric scores
b
Enrichment plot: HOXA9_DN.V1_UP
Enrichment score (ES)
'na_pos' (positively correlated)
Zero cross at 12547
'na_neg' (negatively correlated)
Ranked list metric (PreRanked)
Rank in Ordered Dataset
Enrichment profile — Hits — Ranking metric scores
c
Enrichment plot: RB_P107_DN.V1_UP
Enrichment score (ES)
'na_pos' (positively correlated)
Zero cross at 12547
'na_neg' (negatively correlated)
Ranked list metric (PreRanked)
Rank in Ordered Dataset
Enrichment profile — Hits — Ranking metric scores
d
Enrichment plot: VEGF_A_UP.V1_DN
Enrichment score (ES)
'na_pos' (positively correlated)
Zero cross at 12547
'na_neg' (negatively correlated)
Ranked list metric (PreRanked)
Rank in Ordered Dataset
Enrichment profile — Hits — Ranking metric scores
e
Enrichment plot: ESC_V6.5_UP_EARLY.V1_DN
Enrichment score (ES)
'na_pos' (positively correlated)
Zero cross at 12547
'na_neg' (negatively correlated)
Ranked list metric (PreRanked)
Rank in Ordered Dataset
Enrichment profile — Hits — Ranking metric scores
f
Enrichment plot:
CORDENONSI_YAP_CONSERVED_SIGNATURE
Enrichment score (ES)
'na_pos' (positively correlated)
Zero cross at 12547
'na_neg' (negatively correlated)
Ranked list metric (PreRanked)
Rank in Ordered Dataset
Enrichment profile — Hits — Ranking metric scores
g
Enrichment plot: P53_DN.V1_DN
Enrichment score (ES)
'na_pos' (positively correlated)
Zero cross at 12547
'na_neg' (negatively correlated)
Ranked list metric (PreRanked)
Rank in Ordered Dataset
Enrichment profile — Hits — Ranking metric scores
h
Enrichment plot: MEK_UP.V1_DN
Enrichment score (ES)
'na_pos' (positively correlated)
Zero cross at 12547
'na_neg' (negatively correlated)
Ranked list metric (PreRanked)
Rank in Ordered Dataset
Enrichment profile — Hits — Ranking metric scores
i
Enrichment plot:
VERHAAK_GLIOBLASTOMA_MESENCHYMAL
Enrichment score (ES)
'na_pos' (positively correlated)
Zero cross at 12547
'na_neg' (negatively correlated)
Ranked list metric (PreRanked)
Rank in Ordered Dataset
Enrichment profile — Hits — Ranking metric scores

Figure S11

a

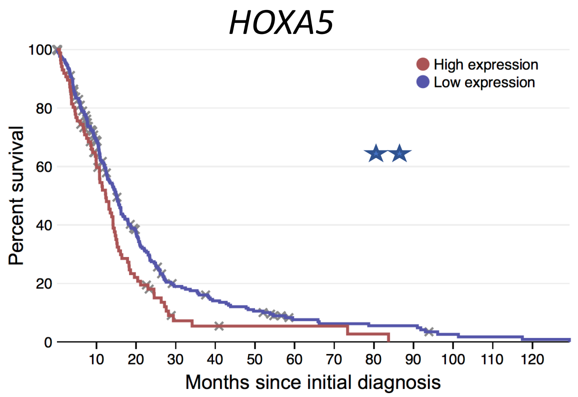

b

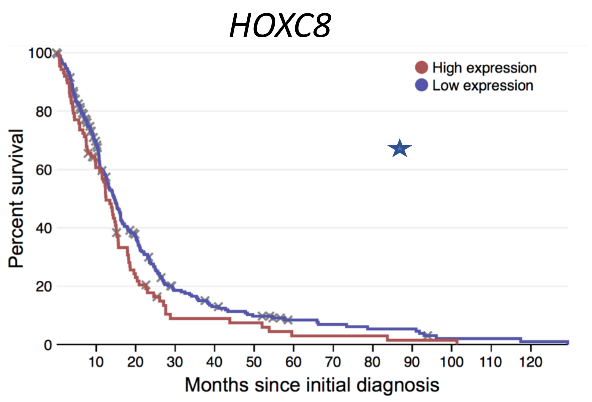

c

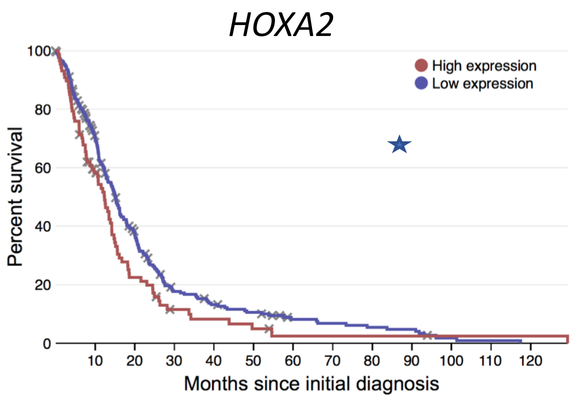

Figure S12

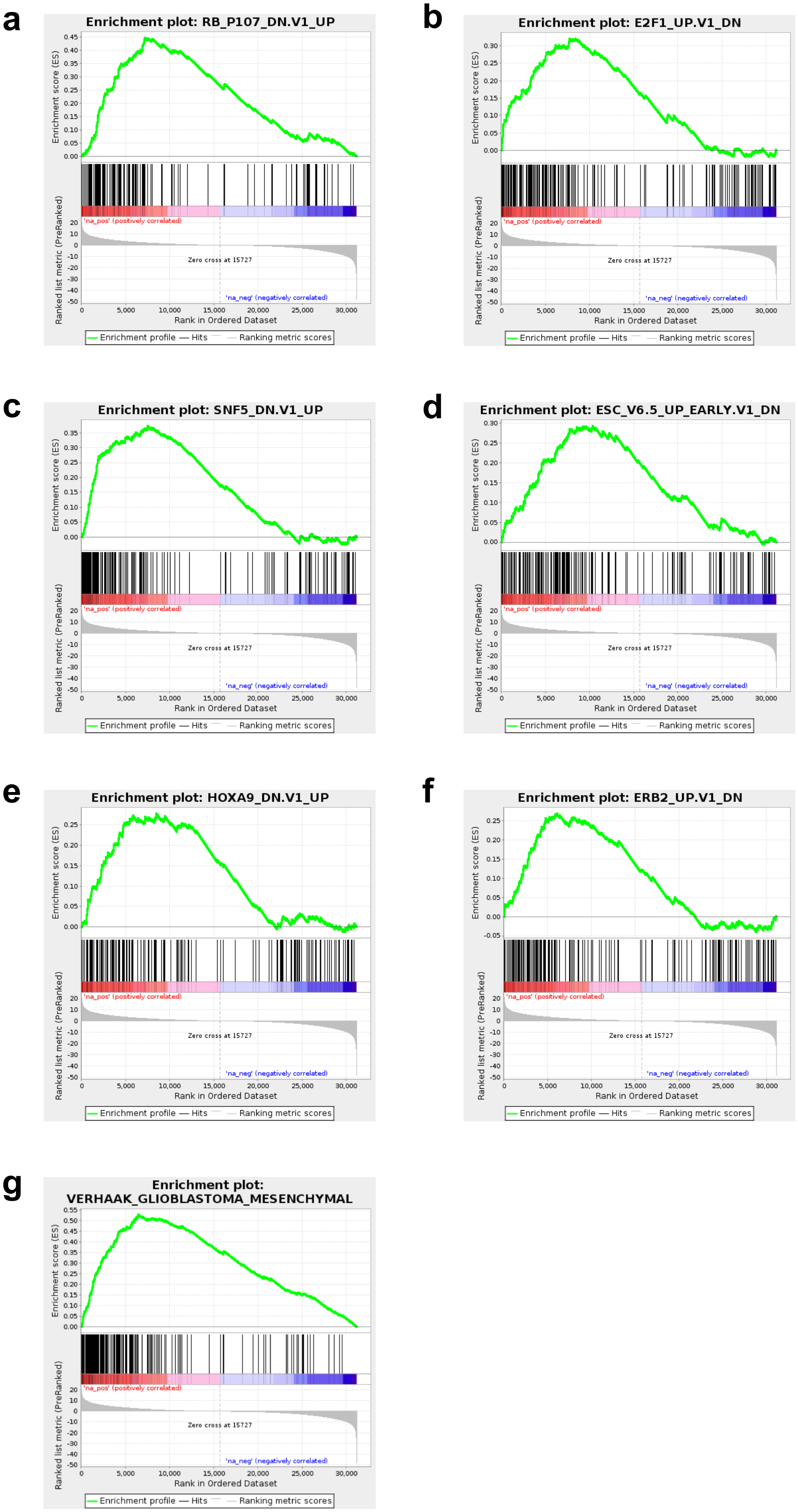
a
Enrichment plot: RB_P107_DN.V1_UP
b
Enrichment plot: E2F1_UP.V1_DN
c
Enrichment plot: SNF5_DN.V1_UP
d
Enrichment plot: ESC_V6.5_UP_EARLY.V1_DN
e
Enrichment plot: HOXA9_DN.V1_UP
f
Enrichment plot: ERB2_UP.V1_DN
g
Enrichment plot: VERHAAK_GLIOBLASTOMA_MESENCHYMAL
Enrichment score (ES)
Ranked list metric (PreRanked)
'na_pos' (positively correlated)
Zero cross at 15727
'na_neg' (negatively correlated)
Rank in Ordered Dataset
Enrichment profile — Hits — Ranking metric scores

Figure S13

a

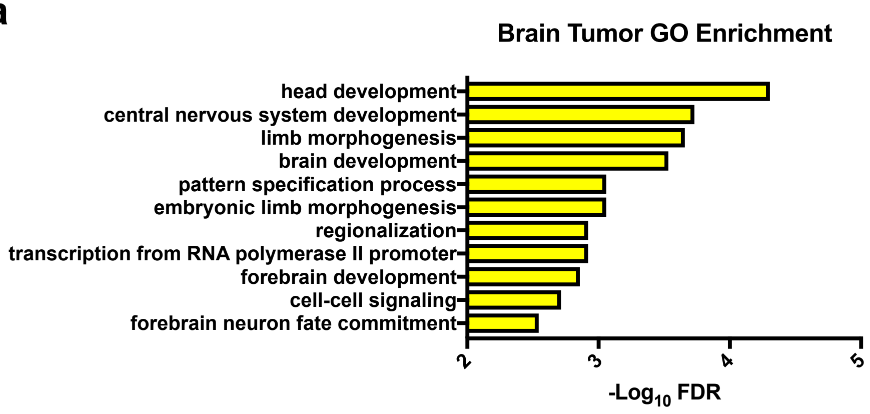

b

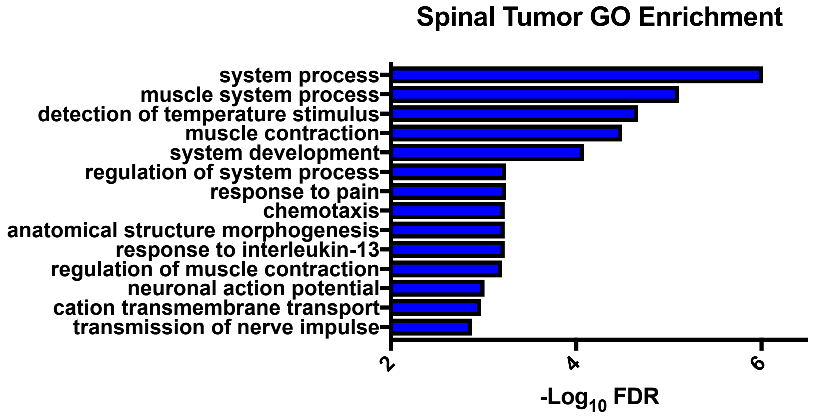

Figure S14

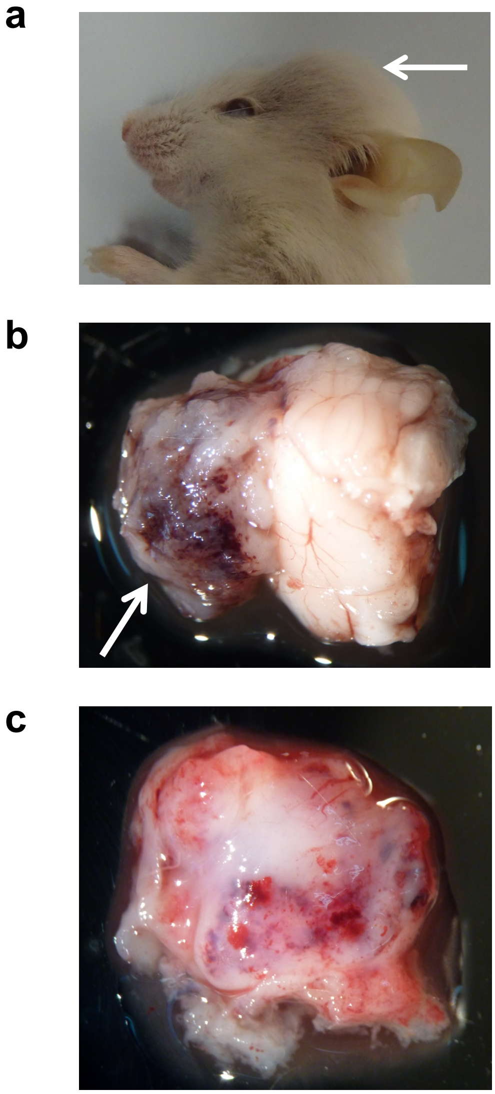

Figure S15

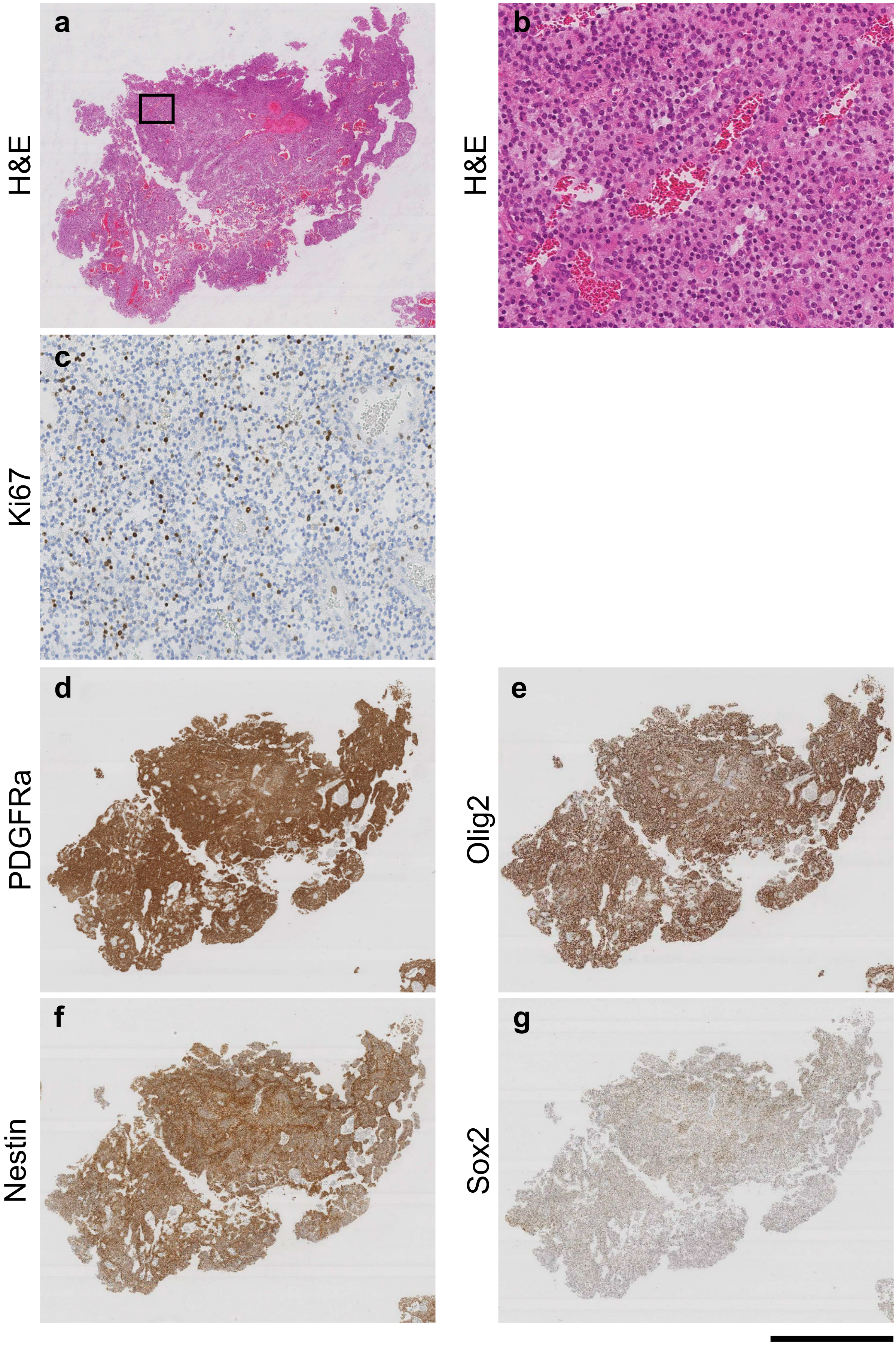

Figure S16

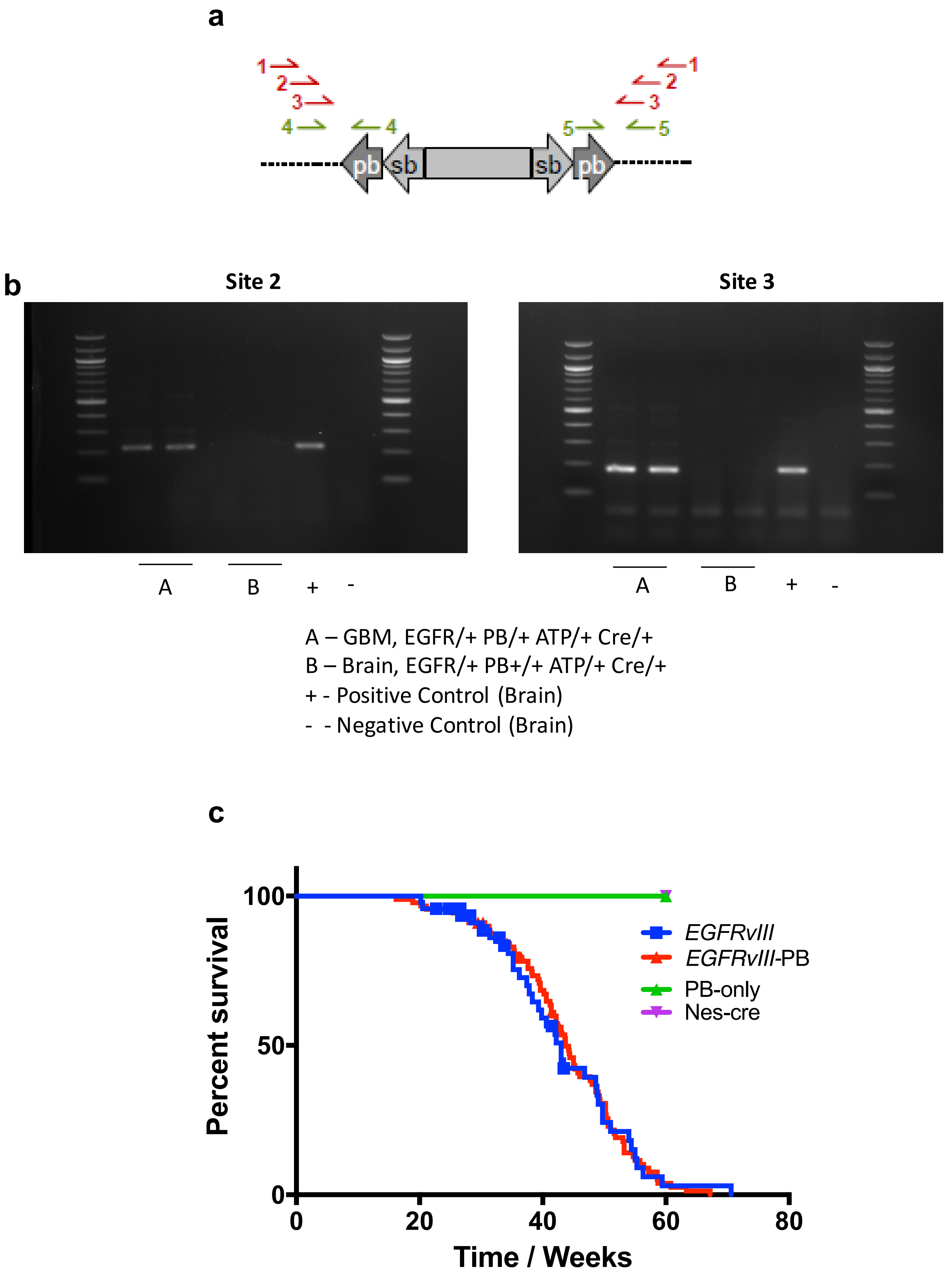
a
1
2
3
4
4
5
5
3
2
1
pb
sb
sb
pb
b
Site 2
Site 3
A
B
+
-
A
B
+
-
A – GBM, EGFR/+ PB/+ ATP/+ Cre/+
B – Brain, EGFR/+ PB+/+ ATP/+ Cre/+
+ - Positive Control (Brain)
- - Negative Control (Brain)
c
Percent survival
100
50
0
0
20
40
60
80
Time / Weeks
EGFRvIII
EGFRvIII-PB
PB-only
Nes-cre

Figure S17

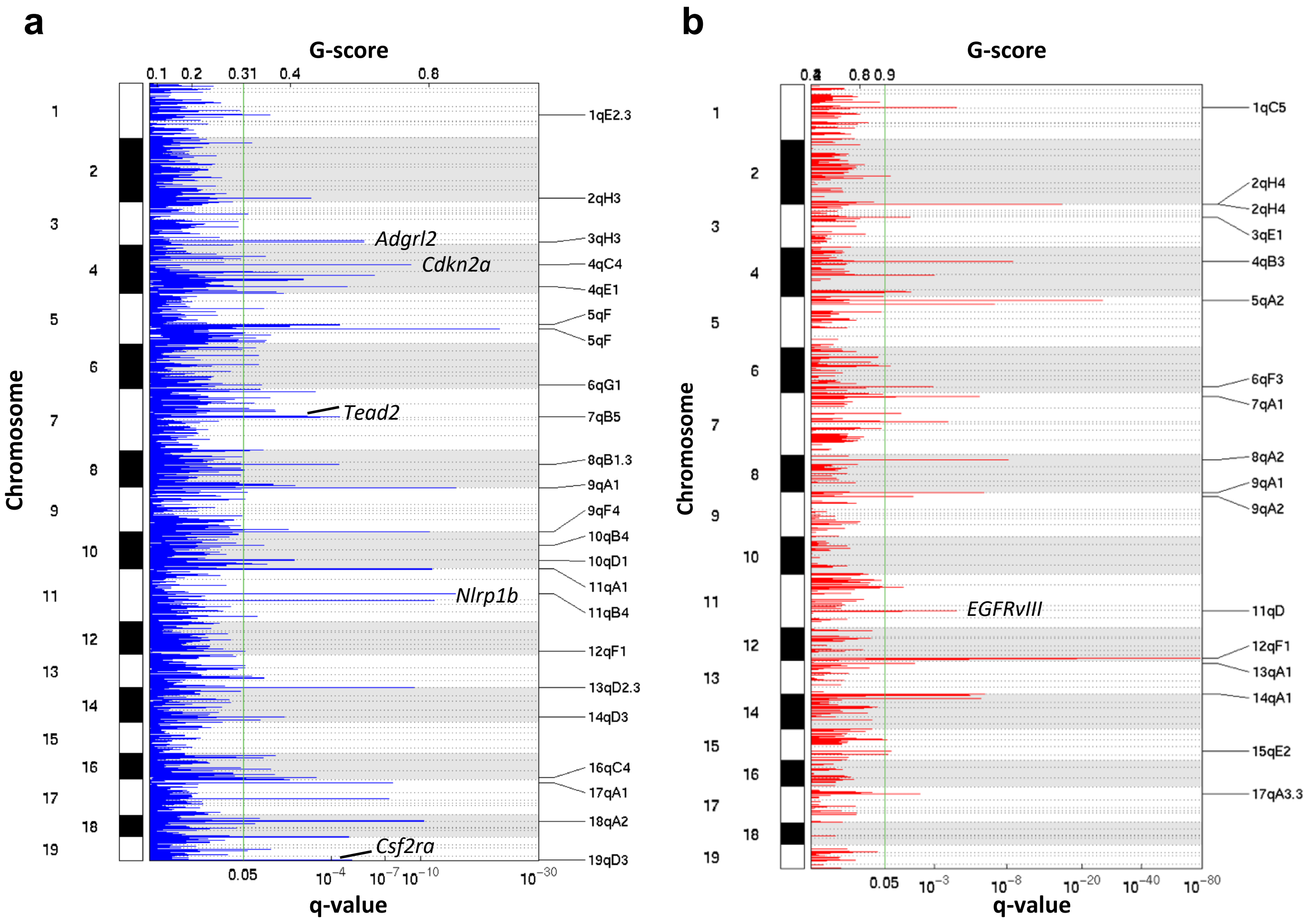
a
G-score
0.1
0.2
0.31
0.4
0.8
Chromosome
q-value
0.05
10^−4
10^−7
10^−10
10^−30
Adgrl2
Cdkn2a
Tead2
Nlrp1b
Csf2ra
1qE2.3
2qH3
3qH3
4qC4
4qE1
5qF
5qF
6qG1
7qB5
8qB1.3
9qA1
9qF4
10qB4
10qD1
11qA1
11qB4
12qF1
13qD2.3
14qD3
16qC4
17qA1
18qA2
19qD3
b
G-score
0.8
0.9
Chromosome
q-value
0.05
10^−3
10^−8
10^−20
10^−40
10^−80
EGFRvIII
1qC5
2qH4
2qH4
3qE1
4qB3
5qA2
6qF3
7qA1
8qA2
9qA1
9qA2
11qD
12qF1
13qA1
14qA1
15qE2
17qA3.3

Figure S18

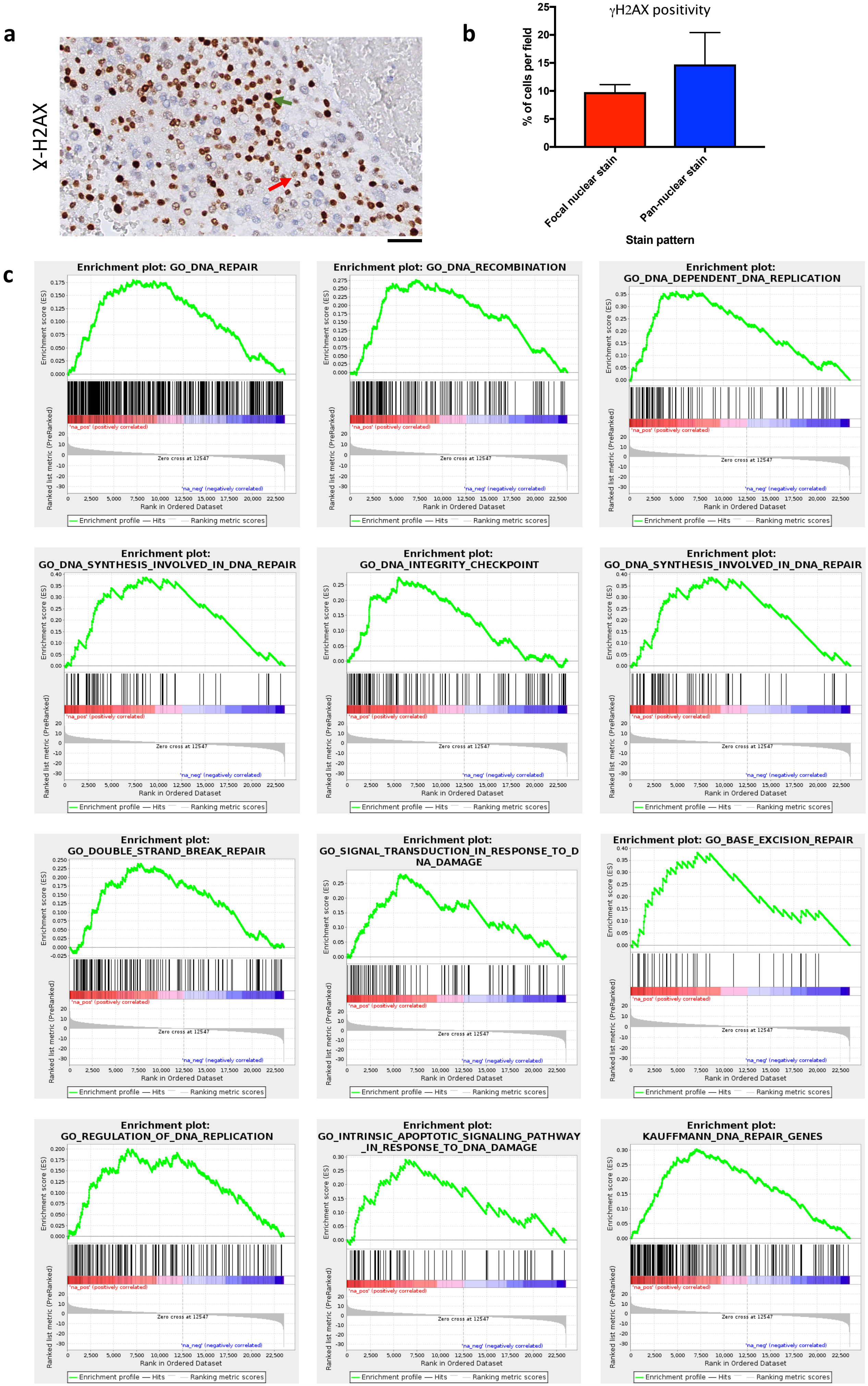
a
γ-H2AX
b
γH2AX positivity
% of cells per field
Focal nuclear stain
Pan-nuclear stain
Stain pattern
c
Enrichment plot: GO_DNA_REPAIR
Enrichment plot: GO_DNA_RECOMBINATION
Enrichment plot: GO_DNA_DEPENDENT_DNA_REPLICATION
Enrichment plot: GO_DNA_SYNTHESIS_INVOLVED_IN_DNA_REPAIR
Enrichment plot: GO_DNA_INTEGRITY_CHECKPOINT
Enrichment plot: GO_DNA_SYNTHESIS_INVOLVED_IN_DNA_REPAIR
Enrichment plot: GO_DOUBLE_STRAND_BREAK_REPAIR
Enrichment plot: GO_SIGNAL_TRANSDUCTION_IN_RESPONSE_TO_DNA_DAMAGE
Enrichment plot: GO_BASE_EXCISION_REPAIR
Enrichment plot: GO_REGULATION_OF_DNA_REPLICATION
Enrichment plot: GO_INTRINSIC_APOPTOTIC_SIGNALING_PATHWAY_IN_RESPONSE_TO_DNA_DAMAGE
Enrichment plot: KAUFFMANN_DNA_REPAIR_GENES
Enrichment score (ES)
Ranked list metric (PreRanked)
'na_pos' (positively correlated)
'na_neg' (negatively correlated)
Zero cross at 12547
Rank in Ordered Dataset
Enrichment profile — Hits — Ranking metric scores

Figure S19

a

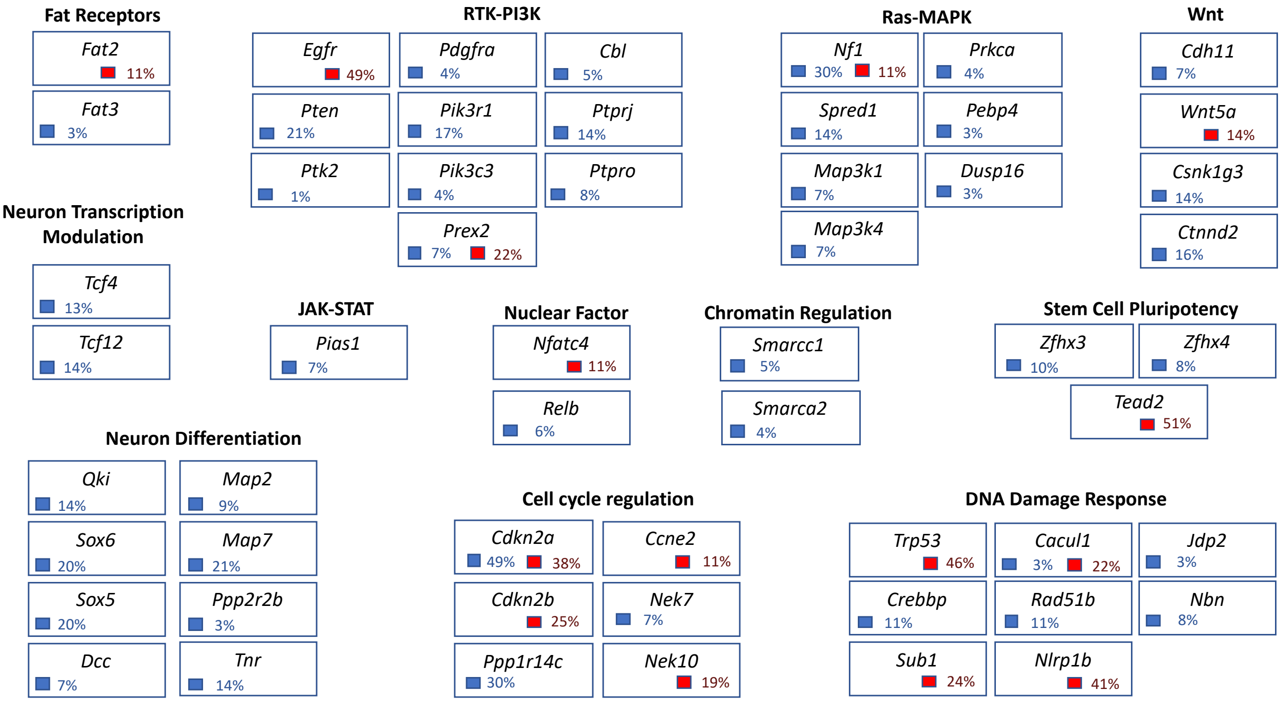

b

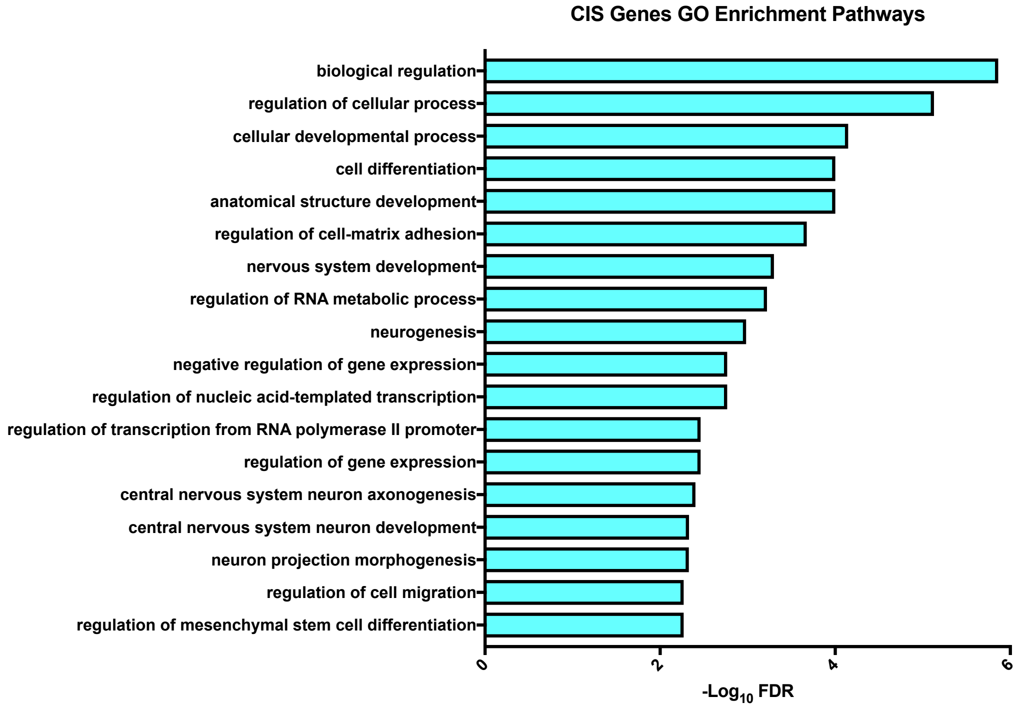

Figure S20

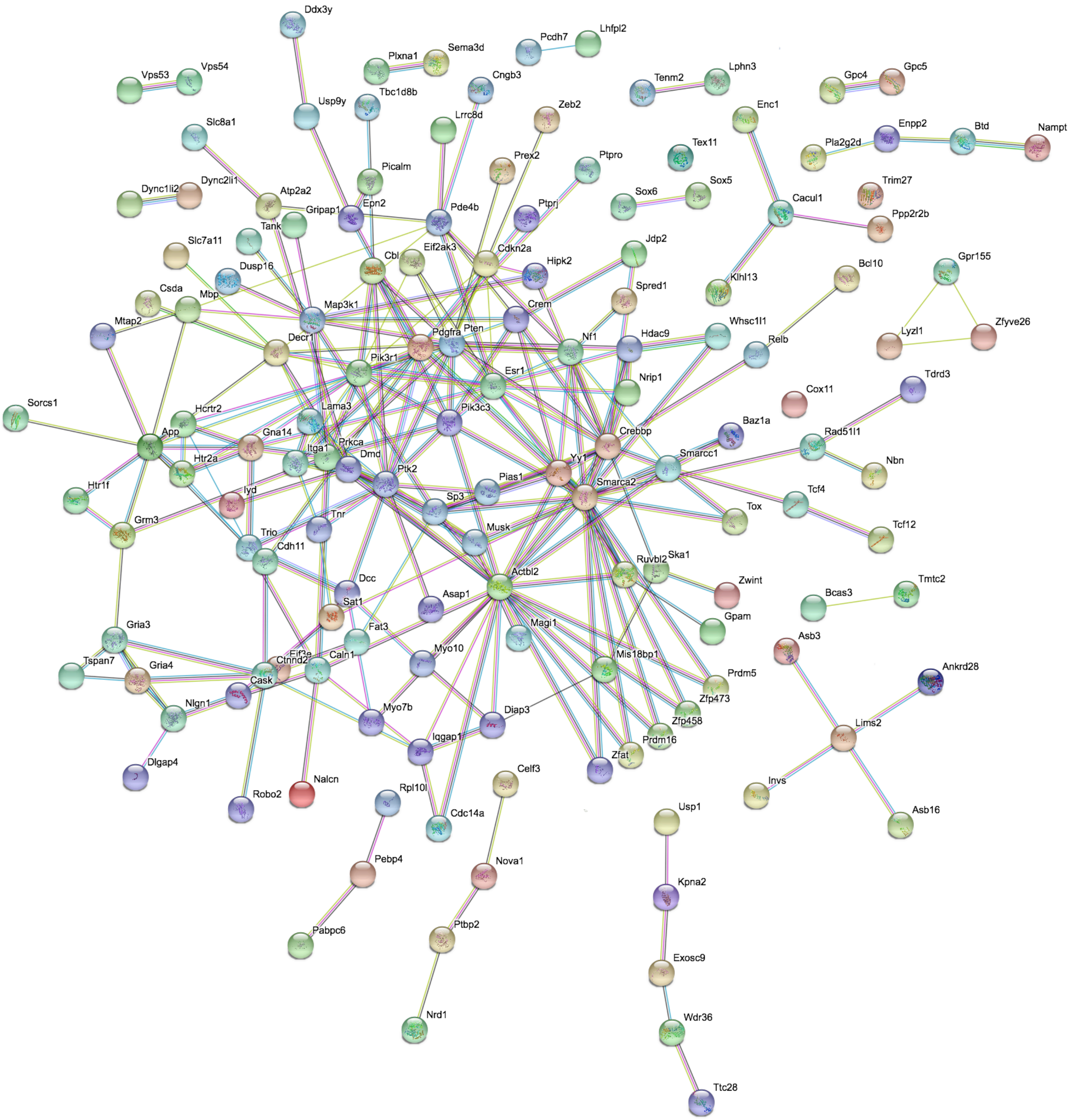
Ddx3y
Pcdh7
Lhfpl2
Sema3d
Plxna1
Vps53
Vps54
Cngb3
Tbc1d8b
Usp9y
Tenm2
Lphn3
Gpc4
Gpc5
Zeb2
Lrrc8d
Enc1
Slc8a1
Enpp2
Btd
Nampt
Pla2g2d
Tex11
Prex2
Ptpro
Picalm
Dync2li1
Dync1li2
Sox5
Trim27
Atp2a2
Epn2
Sox6
Cacul1
Gripap1
Pde4b
Ptprj
Tank
Ppp2r2b
Slc7a11
Jdp2
Eif2ak3
Cdkn2a
Cbl
Dusp16
Hipk2
Bcl10
Gpr155
Klhl13
Csda
Spred1
Mbp
Crem
Map3k1
Zfyve26
Whsc1l1
Mtap2
Pten
Lyzl1
Decr1
Pdgfra
Hdac9
Relb
Nf1
Pik3r1
Esr1
Tdrd3
Nrip1
Cox11
Sorcs1
Lama3
Pik3c3
Hcrtr2
Baz1a
Crebbp
Gna14
App
Rad51l1
Prkca
Smarcc1
Itga1
Yy1
Dmd
Nbn
Htr2a
Pias1
Ptk2
Smarca2
Htr1f
Iyd
Tcf4
Sp3
Tox
Tnr
Grm3
Musk
Tcf12
Trio
Cdh11
Ruvbl2
Ska1
Actbl2
Dcc
Zwint
Tmtc2
Asap1
Bcas3
Sat1
Gpam
Magi1
Fat3
Asb3
Gria3
Myo10
Mis18bp1
Eif3e
Ctnnd2
Caln1
Ankrd28
Prdm5
Tspan7
Gria4
Cask
Zfp473
Nlgn1
Myo7b
Diap3
Lims2
Zfp458
Iqgap1
Prdm16
Zfat
Dlgap4
Nalcn
Robo2
Invs
Celf3
Asb16
Rpl10l
Cdc14a
Usp1
Pebp4
Nova1
Kpna2
Ptbp2
Pabpc6
Exosc9
Nrd1
Wdr36
Ttc28

Figure S21

Pdgfrα

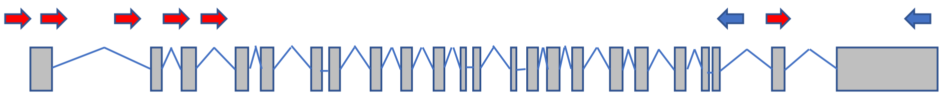

Figure S22

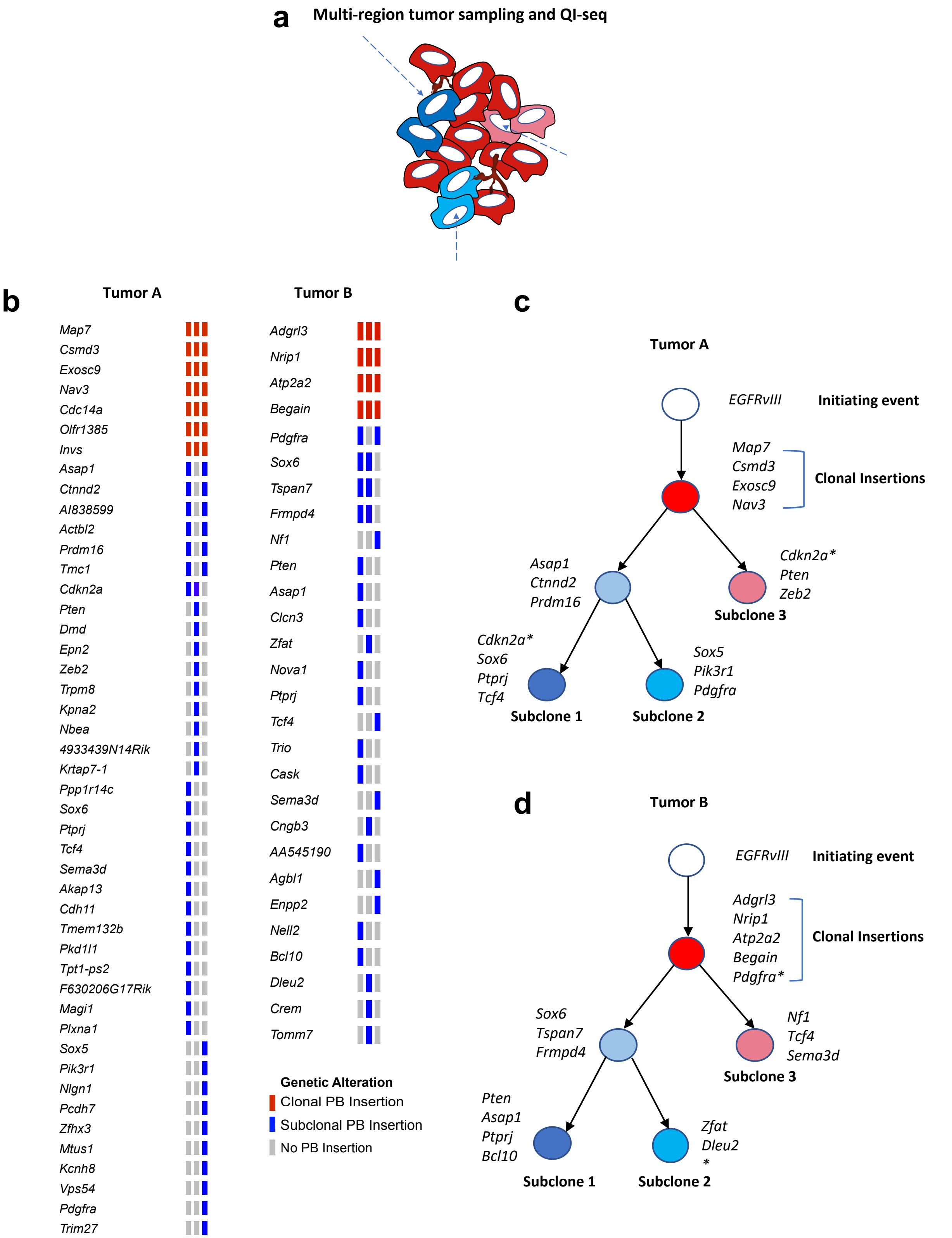
a
Multi-region tumor sampling and QI-seq
b
Tumor A
Map7
Csmd3
Exosc9
Nav3
Cdc14a
Olfr1385
Invs
Asap1
Ctnnd2
AI838599
Actbl2
Prdm16
Tmc1
Cdkn2a
Pten
Dmd
Epn2
Zeb2
Trpm8
Kpna2
Nbea
4933439N14Rik
Krtap7-1
Ppp1r14c
Sox6
Ptprj
Tcf4
Sema3d
Akap13
Cdh11
Tmem132b
Pkd1l1
Tpt1-ps2
F630206G17Rik
Magi1
Plxna1
Sox5
Pik3r1
Nlgn1
Pcdh7
Zfhx3
Mtus1
Kcnh8
Vps54
Pdgfra
Trim27
Tumor B
Adgrl3
Nrip1
Atp2a2
Begain
Pdgfra
Sox6
Tspan7
Frmpd4
Nf1
Pten
Asap1
Clcn3
Zfat
Nova1
Ptprj
Tcf4
Trio
Cask
Sema3d
Cngb3
AA545190
Agbl1
Enpp2
Nell2
Bcl10
Dleu2
Crem
Tomm7
Genetic Alteration
Clonal PB Insertion
Subclonal PB Insertion
No PB Insertion
c
Tumor A
EGFRvIII
Initiating event
Map7
Csmd3
Exosc9
Nav3
Clonal Insertions
Asap1
Ctnnd2
Prdm16
Cdkn2a*
Pten
Zeb2
Subclone 3
Cdkn2a*
Sox6
Ptprj
Tcf4
Sox5
Pik3r1
Pdgfra
Subclone 1
Subclone 2
d
Tumor B
EGFRvIII
Initiating event
Adgrl3
Nrip1
Atp2a2
Begain
Pdgfra*
Clonal Insertions
Sox6
Tspan7
Frmpd4
Nf1
Tcf4
Sema3d
Subclone 3
Pten
Asap1
Ptprj
Bcl10
Zfat
Dleu2
*
Subclone 1
Subclone 2

Figure S23

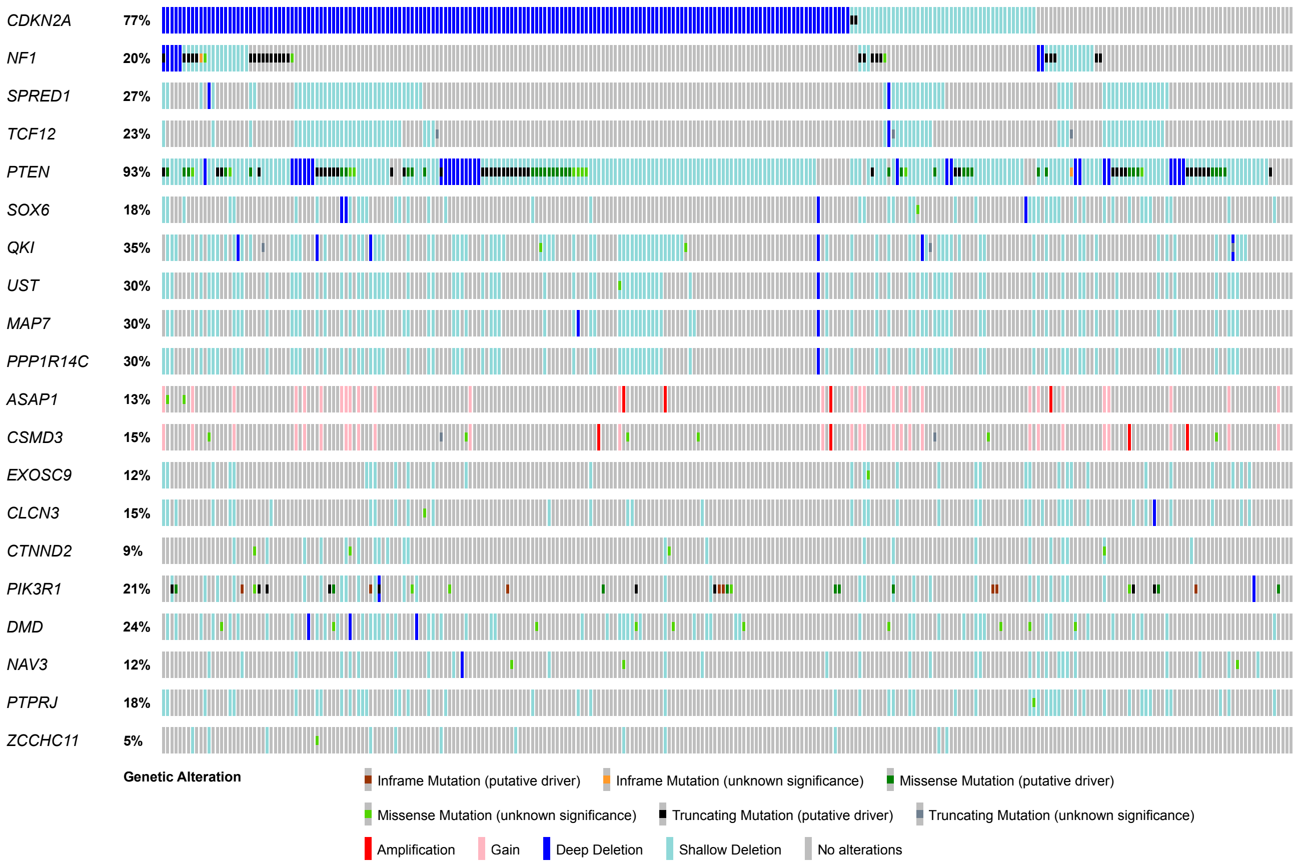
CDKN2A 77%
NF1 20%
SPRED1 27%
TCF12 23%
PTEN 93%
SOX6 18%
QKI 35%
UST 30%
MAP7 30%
PPP1R14C 30%
ASAP1 13%
CSMD3 15%
EXOSC9 12%
CLCN3 15%
CTNND2 9%
PIK3R1 21%
DMD 24%
NAV3 12%
PTPRJ 18%
ZCCHC11 5%
Genetic Alteration
Inframe Mutation (putative driver)
Inframe Mutation (unknown significance)
Missense Mutation (putative driver)
Missense Mutation (unknown significance)
Truncating Mutation (putative driver)
Truncating Mutation (unknown significance)
Amplification
Gain
Deep Deletion
Shallow Deletion
No alterations

Figure S24

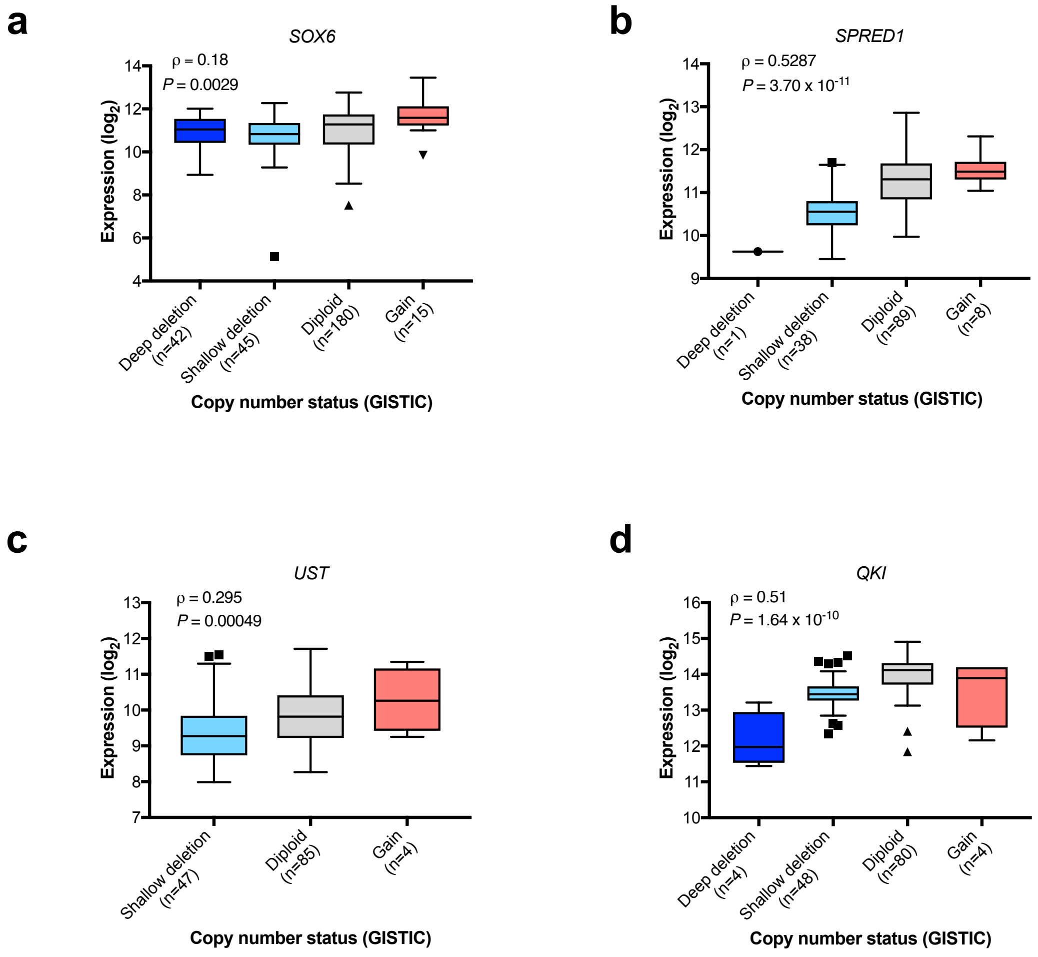
a
SOX6
ρ = 0.18
P = 0.0029
Expression (log2)
Deep deletion (n=42)
Shallow deletion (n=45)
Diploid (n=180)
Gain (n=15)
Copy number status (GISTIC)
b
SPRED1
ρ = 0.5287
P = 3.70 x 10-11
Expression (log2)
Deep deletion (n=1)
Shallow deletion (n=38)
Diploid (n=89)
Gain (n=8)
Copy number status (GISTIC)
c
UST
ρ = 0.295
P = 0.00049
Expression (log2)
Shallow deletion (n=47)
Diploid (n=85)
Gain (n=4)
Copy number status (GISTIC)
d
QKI
ρ = 0.51
P = 1.64 x 10-10
Expression (log2)
Deep deletion (n=4)
Shallow deletion (n=48)
Diploid (n=80)
Gain (n=4)
Copy number status (GISTIC)

Figure S25

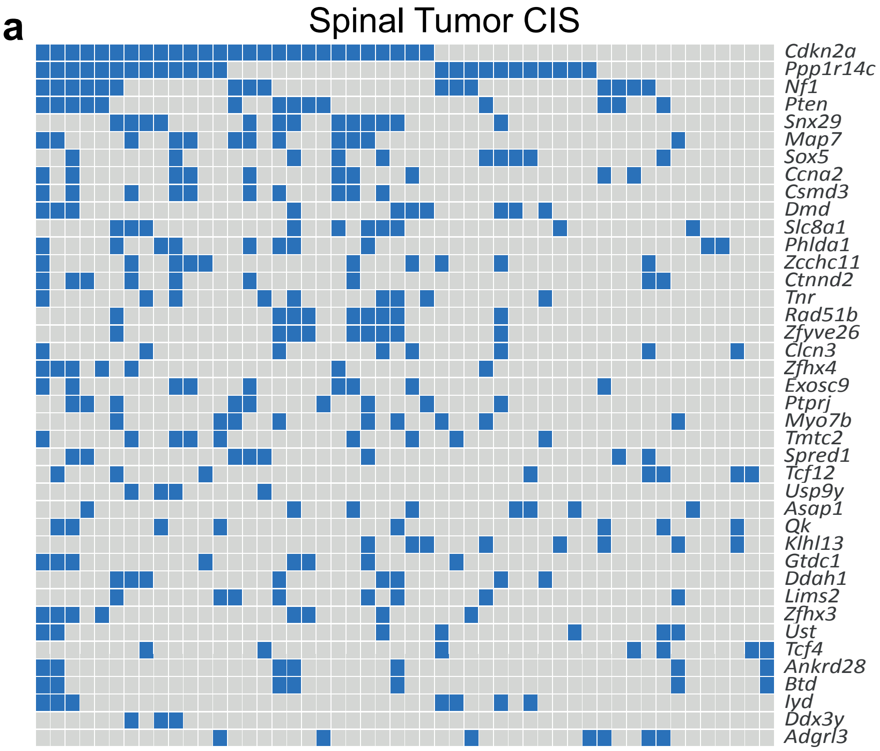
a
Spinal Tumor CIS
Cdkn2a
Ppp1r14c
Nf1
Pten
Snx29
Map7
Sox5
Ccna2
Csmd3
Dmd
Slc8a1
Phlda1
Zcchc11
Ctnnd2
Tnr
Rad51b
Zfyve26
Clcn3
Zfhx4
Exosc9
Ptprj
Myo7b
Tmtc2
Spred1
Tcf12
Usp9y
Asap1
Qk
Klhl13
Gtdc1
Ddah1
Lims2
Zfhx3
Ust
Tcf4
Ankrd28
Btd
Iyd
Ddx3y
Adgrl3

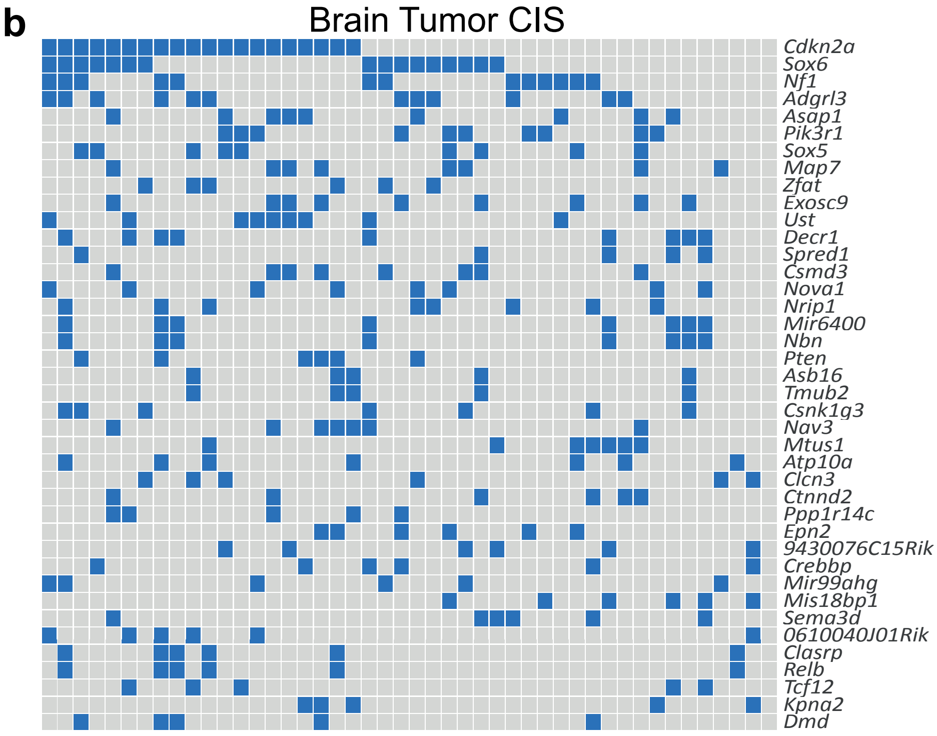
b
Brain Tumor CIS
Cdkn2a
Sox6
Nf1
Adgrl3
Asap1
Pik3r1
Sox5
Map7
Zfat
Exosc9
Ust
Decr1
Spred1
Csmd3
Nova1
Nrip1
Mir6400
Nbn
Pten
Asb16
Tmub2
Csnk1g3
Nav3
Mtus1
Atp10a
Clcn3
Ctnnd2
Ppp1r14c
Epn2
9430076C15Rik
Crebbp
Mir99ahg
Mis18bp1
Sema3d
0610040J01Rik
Clasrp
Relb
Tcf12
Kpna2
Dmd

Figure S26

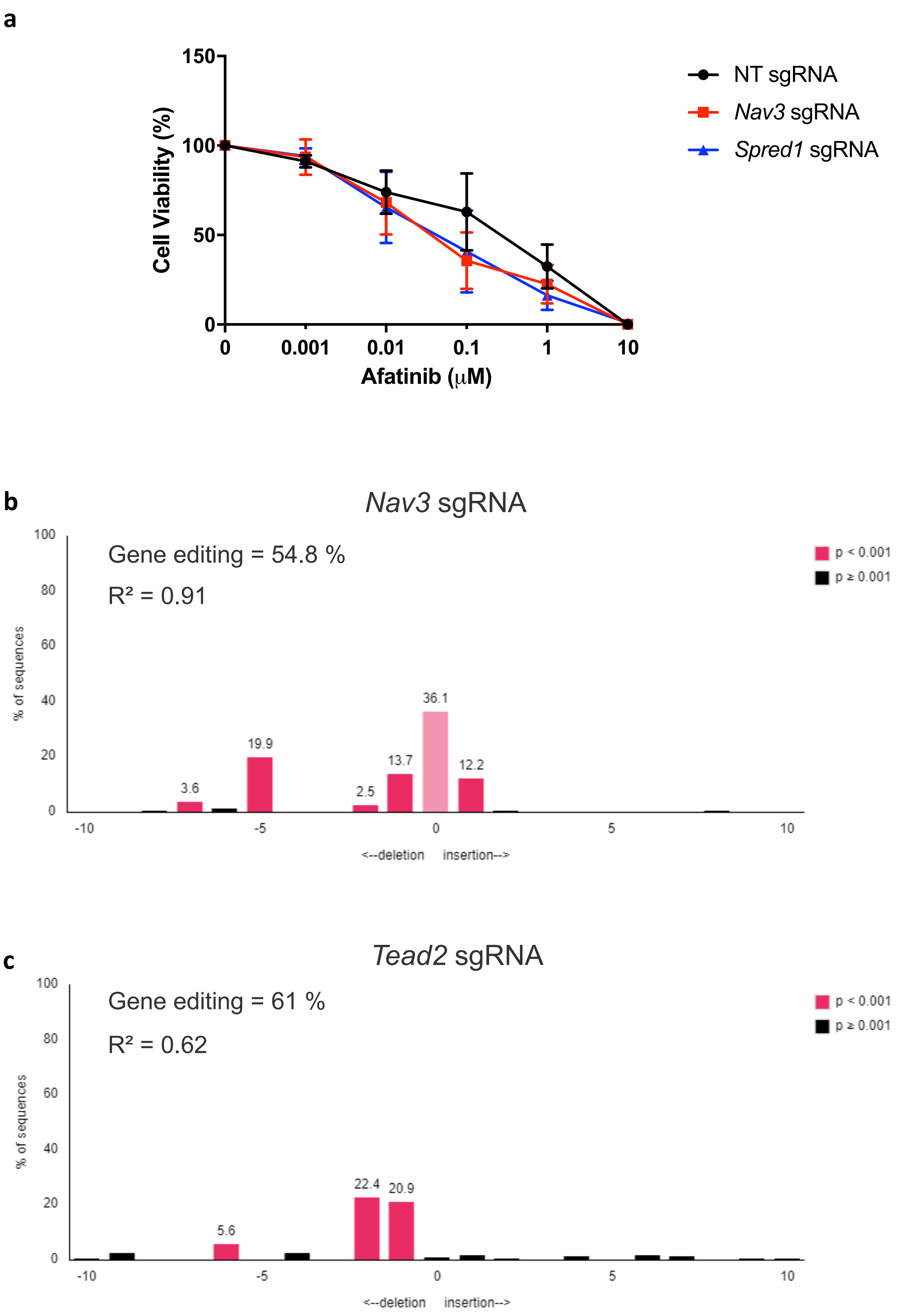
a
150
100
50
0
Cell Viability (%)
0
0.001
0.01
0.1
1
10
Afatinib (μM)
NT sgRNA
Nav3 sgRNA
Spred1 sgRNA
b
Nav3 sgRNA
Gene editing = 54.8 %
R² = 0.91
p < 0.001
p ≥ 0.001
% of sequences
3.6
19.9
2.5
13.7
36.1
12.2
-10
-5
0
5
10
<--deletion insertion-->
c
Tead2 sgRNA
Gene editing = 61 %
R² = 0.62
p < 0.001
p ≥ 0.001
% of sequences
5.6
22.4
20.9
<--deletion insertion-->

Figure S27

Figure S1. Microneoplasias in *EGFRvIII* mouse brains. Examples of the formation of small tumors in the ventricular system and subarachnoid space. A, B, C, tumor growth in the lateral ventricle, the base of the frontal brain and the subventricular zone (SVZ) adjacent to the lateral ventricle. D, E, F, formation of the hypercellular myxoid intrinsic tumor in the third ventricle (D) the lateral ventricle (E) and the base of the pons (F, arrows). G, H, I, hypercellular cluster (dark nuclei of expanded SVZ stem/progenitor cells (green arrows) and adjacent a small glial neoplasm (blue arrows). H, small glioma protruding from the floor of the 3rd ventricle and I, subarachnoid spread of a glial neoplasm on the base of the pons, in a "sugarcoat" fashion (arrows). Lettering on sides of panels reflect mouse IDs from which these tumor originated. Scale bar corresponds to 150μm for A and B, 75μm for C, 100μm for D, E and F, 50μm for G, 100μm for H and I.

Figure S2. Expansions of neural progenitor cells in the SVZ of *EGFRvIII* mice. Representative immunostaining for neural progenitor and stem cell markers of the SVZ from an 8-week old *EGFRvIII* mouse. Strong positivity is detected for GFAP (A), Sox2 (B) and Olig2 (C). Some positivity is also observed for PDFGRα (D), double-cortin (DCx, E) and nestin (F), correlating with cellular expansion of the SVZ. In the same mouse, possible incipient microneoplasias are seen in the SVZ (G), staining positive for Sox2 (H) and Olig2 (I). Scale bar corresponds to 50μm for A-F, 100μm for G-I.

Figure S3. Expression of human EGFR, as detected by immunostaining, is limited to tumor cells in EGFRvIII mouse brains. Overview (left) and detail (right) of tumors and microneoplasias of different sizes and locations. A, B, medium-sized circumscribed, extraparenchymal growing neoplasm attached to the temporal lobe. B, detail showing strong and diffuse EGFR expression specifically in the tumor. C, D, small circumscribed tumor growing on the floor of the third ventricle and expanding towards the optic tract. E, F, transformed cells, with possibly incipient formation of microneoplasia in the left lateral ventricle. G, H, scattered small neoplastic lesion on the floor of the midbrain. Scale bar corresponds to 0.7mm for A, 200μm for B, 0.4mm for C, 200μm for D, 0.7mm for E, 100μm for F, 0.5mm for G, 200μm for H.

Figure S4. Expression of human EGFRvIII is limited to tumor cells. A, B, overview and detail images demonstrating EGFRvIII immunostaining is positive across glioma cells but not normal mouse brain in *EGFRvIII*; *nes*-cre mice (n=4). C, D, overview and detail imaged demonstrating *EGFRvIII* is expressed in smaller glioma nests (precursors to larger tumors) in these mice. Scale bar corresponds to 1mm for A and C, and 100μm for B and D.

Figure S5. Immunostaining for markers of neural progenitors. A typical *EGFRvIII* mouse microneoplasia (INEG8.3D) is shown in the left panels: A, nuclear Sox 2 expression, B, nuclear Olig2 expression, C, nestin expression in a diffuse cytoplasmic pattern, D, expression of GFAP in reactive astrocytes, interdigitating between tumor cells. E, strong, diffuse expression of PDGFRa in all tumor cells. The right panels show a typical small glioma. F, overview of the coronal brain section with a circumscribed extracerebral intrinsic tumor, highlighted with an immunostaining for EGFR. G, detail of the tumor, stained for EGFR. H, immunostain for GFAP shows negative

tumor cells enclosing a strand of reactive glial tissue. All tumor cells strongly express PDGFRa (I), Olig2 (J), and Nestin (K). Scale bar corresponds to 50μm for A, B, C, D, E, G, H, I, J, K; 1.3mm for F.

Figure S6. Histological features of GBM in *EGFRvIII*-mice. Typical examples showing defining histological features of glioblastoma in two mouse tumors – A, B show microvascular proliferation and necrosis in one GBM, and C, D show these features in another. Scale bar corresponds to 50μm.

Figure S7. Proliferative capacity of microneoplastic cells in the SVZ and brain surface of *EGFRvIII*-mice. Overview (A) and high-power views are shown of representative Ki67-stained sections of the SVZ and brain surface. B shows the presence of Ki67 positive cells in a brain surface microneoplasia. C is an overview of the SVZ stained for Ki67; D is a high power view of a microneoplasia from this region with positive cells for Ki67, E shows some positive cells in a cellular expansion in the SVZ. Scale bar corresponds to 0.8mm for A, 70μm for B, 150μm for C, 50μm for D and E.

Figure S8. Spinal gliomas are induced on the surface of the spinal cord of *EGFRvIII* mice. H&E stained sections: A shows overview and B shows high power view of a representative tumor, with features of a leptomeningeal-invading glioma. Scale bar corresponds to 0.7mm for A and 70μm for B.

Figure S9. Mutations of *Trp53* and *Tead2* in *EGFRvIII*-only and *EGFRvIII*-PB gliomas are in DNA-binding domains. A. Plot outlining the location of *Trp53* mutations across all exome-sequenced mouse tumors. Five *EGFRvIII*-only and two *EGFRvIII*-PB tumors had in *Trp53*, all residing within its DNA-binding domain; 3 occurred in the same location. B. Plot outlining locations of *Tead2* mutations, all residing in the TEA/ATTS domain which is the DNA-binding domain of *Tead2*; 2 mutations were in same splice site location.

Figure S10. Comparative genomics of mouse *EGFRvIII* glioma mutations with human gliomas. Using the TCGA dataset of 283 patients demonstrates that several of the most frequently mutated genes in EGFRvIII mouse gliomas are found methylated or genetically altered with high frequency in human low-grade gliomas (LGGs). A. Plot showing genes *NLRP1*, *SUB1, CES1* and *ITGA6* are commonly methylated in human LGGs (medians and interquartile ranges are displayed; the methylation levels for the genes are significantly higher than for *EGFR,* $p < 0.0001$, paired t-test). B. Oncoprint demonstrating the high frequency of genetic alterations across all tumors in this dataset for *TP53*, *TEAD2*, *NT5C2, ADGRL2,* and *UIMC1*.

Figure S11. Gene set enrichment analysis (GSEA) of *EGFRvIII* brain gliomas defines their key oncogenic pathways. Plots are displayed for a selection of significantly enriched gene sets, including *SNF5*, *HOXA9*, *RB*, *VEGF*, *ESC*, *YAP*, *P53* and *MEK* gene sets (Benjamini-Hochberg adjusted $p < 0.01$). I. Transcriptional profile of these tumors are significantly enriched for the Verhaak human mesenchymal glioblastoma profile (Benjamini-Hochberg adjusted $p < 0.01$).

Figure S12. *Hox* gene upregulation correlates with poor prognosis in glioblastoma patients. The large human TCGA GBM dataset of 273 patients was analyzed for survival correlations. *Hoxa5*, *Hoxc8* and *Hoxa2* are amongst the top 5 most overexpressed genes in *EGFRvIII*-mouse brain tumors, and this analysis shows that overexpression of these genes correlates with significantly worse overall survival in GBM patients ($p < 0.05$, log-rank test).

Figure S13. Gene set enrichment analysis of *EGFRvIII* spinal gliomas defines their key oncogenic pathways. Plots for a selection of significantly enriched gene sets are presented here, including *Rb*, *E2F1*, *SNF5*, *ESC* (embryonic stem cell), *HOXA9*, and *ERBB2* gene sets (Benjamini-Hochberg adjusted $p < 0.01$). G. Transcriptional profile of these tumors are significantly enriched for the Verhaak human mesenchymal glioblastoma profile (Benjamini-Hochberg adjusted $p < 0.01$).

Figure S14. Gene ontology (DAVID) analysis of differentially expressed genes in *EGFRvIII* mouse brain and spinal gliomas. In brain tumors, there is enrichment for gene sets reflecting brain developmental processes, whereas in spinal tumors the gene sets reflect processes intrinsic to the spinal cord. These data reflect the different tissue origins of these tumors; note the absence of gene sets for oncogenic pathways here, as these are largely shared between the two types of tumor. FDR – false discovery rate.

Figure S15. Clinical Phenotype of *EGFRvIII*-PB mice. A. Photograph of an *EGFRvIII*-PB mouse with enlarged head due to an underlying brain glioma. B, C. Macroscopic photographs of areas of the brain from the same mouse showing the presence of a tumor on the brain surface.

Figure S16. Typical example of a grade III brain glioma from an *EGFRvIII*-PB mouse. Overview (A) and high power (B) H&E-stained histological sections of a brain tumor from the mouse previously shown in Supplementary Figure S13. C. This brain tumor has a higher proliferative index (Ki67 staining) than earlier microneoplasias, in this case estimated to be 10-20% across the tumor. This tumor contains many cells expressing neural stem and progenitor markers PDGFRα (D), Olig2 (E) and nestin (F), with some cells also expressing Sox2 (G). Scale bar corresponds to 2.5mm for A, D, E, F, G; 200μm for B; 150μm for C.

Figure S17. Transposon mobilization in *EGFRvIII*-PB mice and survival times. A. PCR assay to detect mobilization or lack of excision of the ATP1S2 transposon. B. Examples of PCR results showing mobilization of the transposon in GBMs from mice containing PB, and absence of transposition in brains from mice without PB; only genotyping from sites 2 and 3 (referenced in A) are shown here. Fragment sizes: 220bp for site 2; 182bp for site 3; 100bp ladder. C. Kaplan-Meier survival curves of *EGFRvIII*-only (n=48) and *EGFR*-PB (n=72) mice, with no significant difference between them ($p = 0.95$, log-rank test). No differences in survival or pathology were observed between *EGFRvIII*; *nes-cre* and *EGFRvIII*; *nes-cre*; ATP1S2 mice. Tumors were not observed in PB-only (TSPB; ATP1S2; *nes*-cre, n=20) or *nes*-cre (n=10) mice after 60 weeks.

Figure S18. Plots showing focal copy number variations across *EGFRvIII*-only and *EGFRvIII*-PB mice. Significant focal deletions as determined by GISTIC2 are displayed in A, and significant focal

amplications are displayed in B. Lower x-axis represents q-value (significance at < 0.05) and top x-axis represents the G-score.

Figure S19. Evidence of replication stress and activation of DNA damage response pathways. A. Immunostaining for γH2AX in *EGFRvIII*-mouse gliomas. High power view of a typical immunostain of a tumor; green arrow shows pan-nuclear staining of a tumor cell and red arrow indicates a typical pattern of focal nuclear staining. Scale bar 25μm B. Quantification of γH2AX in *EGFRvIII*-mouse gliomas, including focal and pan-nuclear staining. Bars represent mean values (n=2 tumors with three views per tumor) +/- SEM. C. RNA-seq analysis using gene set enrichment analysis (GSEA) of mouse *EGFRvIII*-gliomas shows significant enrichment for pathways involved in the DNA damage response, as indicated. Each enriched pathway has an FDR q-value < 0.001.

Figure S20. Key genes from *PiggyBac* mutagenesis and whole-exome-sequencing cluster into oncogenic pathways. A. Oncogenic pathways driving *EGFR*-mutant glioma progression are displayed. Blue boxes depict the percentage of tumors containing a *PiggyBac* insertion for a particular CIS gene; red boxes show the percentage of tumors with a mutation or CNV detected by WES. All pathways have at least one gene targeted by transposons ($p < 0.0001$, Kernel convolution analysis). Analysis using *Panther* showed that key genes are grouped into oncogenic pathways. B. David gene ontology (GO) analysis of all 281 glioma CIS genes shows significant enrichment for pathways including neurogenesis and mesenchymal stem cell differentiation, suggesting these pathways are important in driving *EGFR*-mutant gliomagenesis (FDR = false discovery rate).

Figure S21. Network analysis of all interacting CIS transposon genes. An analysis, performed using STRING, to determine the functional connectivity between CIS genes demonstrates there are 253 interactions between their proteins, showing *PiggyBac* mutagenesis has identified mutations in functionally interacting proteins (Benjamini-Hochberg adjusted $p = 4.88 \times 10^{-13}$, Hypergeometric test). Color coding: colored nodes are proteins from CIS genes; connecting lines are known or predicted interactions between proteins; see https://string-db.org for further details.

Figure S22. Insertional pattern consistent with *Pdgfrα* gene activation in brain tumors. *PiggyBac* transposons from all *EGFRvIII*-PB gliomas are largely at the start of the gene in the forward orientation, with only two at the last exons of the gene (likely to be of lesser functional significance), suggesting the transposons are driving transcriptional activation.

Figure S23. *EGFRvIII*-PB gliomas display intratumor heterogeneity, and PB insertions identify their evolutionary routes. A. Overview of the experiment: two gliomas were sampled from three independent regions each, and their DNA was subjected to QI-seq to determine their insertions. Only insertions in CIS genes (determined to be significant across all 96 tumors) were included in this analysis. B. The insertional patterns from tumor A (a low grade glioma on histopathology) and tumor B (a glioblastoma) from all three regions are displayed on this oncoprint, with clonal PB insertions (found in all regions of the tumor) colored red and subclonal ones (found in some

regions of the tumor but not all) colored blue. C. Tumor A shows branching evolution, with truncal clonal insertions in genes including *Map7*, *Csmd3*, *Nav3* and *Exosc9*. *Subclones 1 and 3 have different *Cdkn2a* insertions, implying these arose later and independently in evolution. D. Tumor B similarly shows branching evolution, with distinct clonal and subclonal PB insertions. *Subclones 1 and 3 have the same *Pdgfra* insertion, but subclone 2 does not suggesting *Pdgfra* was likely a truncal insertion that subclone 2 later lost due to continued PB transposition.

Figure S24. Comparative genomic analysis confirms the presence of genetic alterations in human GBMs for the top CIS genes. Data were analyzed from the TCGA high-grade glioma dataset of 273 patients, confirming that for most of the top CIS genes there is a high frequency of alterations (particularly copy-number changes) in these patients.

Figure S25. Deletions in putative tumor suppressors are associated with reduced expression. A – D. Correlation of expression levels of *SOX6* in LGGs (A), *SPRED1* (B), *UST* (C) and *QKI* (D) in GBMs with their respective copy number levels using the entire TCGA human datasets (RNA-seq data available for n=282 LGGs and n=136 GBMs) in order to provide adequate sample sizes. Boxes span the third (Q3) quartile to the first (Q1) quartile (interquartile range, IQR), with the line at the median; whiskers extend to Q3 + 1.5 x IQR and Q1 – 1.5 x IQR. Outliers are plotted as individual points. Spearman's rank correlation was used to calculate correlation coefficients (ρ) and *P* values. These data imply deletions of these genes result in loss of their expression, supporting their roles as tumor suppressors.

Figure S26. *PiggyBac* mutagenesis identifies *EGFRvIII* cooperative genes in brain and spinal tumors. A. Oncoprint showing the top CIS genes for spinal tumors, ranked according to the total number of insertions. B. Oncoprint for the top CIS genes in brain tumors. Note that *Pten* ranks very highly in spinal tumors but ranks lower in brain tumors (not seen in this oncoprint), where in contrast there are some alternative drivers ranking highly such as *Sox6* and *Pik3r1*.

Figure S27. Treatment of gliomaspheres with afatinib in the presence of CRISPR-Cas9 induced mutations. A. *EGFRvIII*-mouse gliomaspheres were treated with afatinib at the concentrations shown. Wild-type cells (with a non-targeting sgRNA, NT sgRNA), as well as cells with *Nav3* and *Spred1* loss-of-function mutations were treated. No significant difference in cell viability was observed between these conditions. Significance testing was done with the two-sided t-test, and $p < 0.05$ was deemed significant. B. TIDE confirms on-target indels for *Nav3* created by CRISPR-cas9, and also for *Tead2* (C). The bar plots indicate the percentage of sequences with indels at the loci indicated around the sgRNA target site.

Table S1. Mouse Phenotypes. Outline of clinical and histological details of mice in this study.

Table 2. Significantly mutated genes. All recurrent significantly mutated genes are presented.

Table S3. Significant deletions. Focally deleted regions are presented.

Table S4. RNA-seq in brain tumors. Results of analysis of RNA-seq data from brain tumors of *EGFRvIII*-only mice.

Table S5. RNA-seq in spinal tumors. Results of analysis of RNA-seq data from spinal tumors of *EGFRvIII*-only mice.

Table S6. CIS genes. All common integration sites from piggyBac in both brain and spinal tumors.

Table S7. Fusion Transcripts. Results from RNA-seq analysis identifying fusion transcripts between *piggyBac* and endogenous genes.

Table S8. CanSAR analysis for druggable genes. All druggable genes from the glioma network are presented. Dark green = targets with clinically approved drugs available; light green = targets of investigational drugs; yellow = targets of drugs under chemical investigation; light red = targets that are predicted to be druggable by chemistry-based assessment.

Table S9. Glioma drug targets identified from CanSAR analysis of PB CIS driver genes and recurrently mutated genes. Proteins in bold typeface are those that have been targeted with drugs in human glioma cell lines with at least partial efficacy.

Table S10. GDSC analysis for sensitivity of human glioma cell lines to drugs targeting the glioma network.

Table S11. Sequences for CRISPR sgRNAs used in this study.
