# Supplementary material for: PiggyBac mutagenesis and exome sequencing identify genetic driver landscapes and potential therapeutic targets of EGFR-mutant gliomas
Source: Genome Biol. 2020 Jul 30;21:181. doi: 10.1186/s13059-020-02092-2 (PMC7392733; doi:10.1186/s13059-020-02092-2)
Supplement: Supplementary file 10 — Additional file 10: Table S9. Chemogenomic analysis. [file 13059_2020_2092_MOESM10_ESM.docx]

| **Targets of approved drug (n=14)**  **EGFR**, **ESR1**, GLP2R, GRIA3, GRIA4, HCRTR2, **HDAC9**, HTR1F, HTR2A, **MEK1**, **MEK2**, PDE4B, **PDGFRA**, TRPM8 |
| --- |
| **Targets of investigational drug (n=3)**  **AKT1**, **AURKC**, PRKCA |
| **Targets being investigated chemically (n=34)**  **APP**, CASK, CCNA2, CCNB3, CREBBP, CSNK1G3, EIF2AK3, ENPP2, ERAP1, ERK1, **ERK2**, FNTB, GRM3, HIPK2, HRAS, ITGA1, MAP2, MAP3K1, MAP3K4, MMP16, MUSK, MYT1, **NAMPT**, NEK7, **PIK3C3**, **PIK3R1**, PLA2G2D, PLK4, PTK2, SLC7A11, SLC8A1, **SMARCA2**, TP53, USP1 |
| **Predicted targets by structure-based method (n=96)**  ABCA13, ABHD2, ADIPOR1, AKAP13, ANKRD28, ANKRD40, ARAP1, ARHGEF28, ASAP1, ATP10A, ATP2A2, BAZ1A, BAZ1B, BCL10, BICC1, CBL, CDC14A, CDH11, CSMD1, CSMD3, CUL2, CXADR, DCC, DDAH1, DDX3Y, DECR1, DGKB, DMD, DYNC1LI2, EIF3E, ENC1, EPN2, ESYT2, FAT3, FSTL5, GPR85, IL1RAPL1, INVS, ITGAM, JDP2, KCNH8, KCNJ16, KDM1B, KRAS, LAMA3, LIMS2, LRRC8D, MAGI1, MAGI2, MYO10, MYO7B, NALCN, NAV3, NBEA, NCKAP5, NF1, NFS1, NRAS, NT5C2, PARP8, PCLO, PDZRN4, PKD1L1, PPP2R2B, PRCC, PRDM16, PRDM5, PSAT1, PTBP2, PTEN, PTP4A2, PTPRJ, PTPRO, RUVBL2, SAT1, SEMA3D, SND1, SNX29, SP3, SPAG17, TANK, TBC1D8B, TDRD3, TEAD2, TENM2, TENM3, TGS1, TNR, TOX, TTC28, TTC39B, USP9Y, VPS54, ZEB2, ZFHX4, ZFYVE26 |

**Table 1. Glioma drug targets identified from CanSAR analysis of PB CIS driver genes and recurrently mutated genes.** Proteins in bold typeface are those that have been targeted with drugs in human glioma cell lines with at least partial efficacy.
